# Transcriptome analysis of *Xanthomonas fragariae* in strawberry leaves

**DOI:** 10.1038/s41598-020-77612-y

**Published:** 2020-11-25

**Authors:** Joanna Puławska, Monika Kałużna, Wojciech Warabieda, Joël F. Pothier, Michael Gétaz, Jan M. van der Wolf

**Affiliations:** 1 Department of Phytopathology, Research Institute of Horticulture, 96-100 Skierniewice, Poland; 2 Environmental Genomics and Systems Biology Research Group, Institute for Natural Resource Sciences, Zurich University of Applied Sciences (ZHAW), Wädenswil, Switzerland; 3 Wageningen University & Research, Wageningen, The Netherlands

**Keywords:** Microbiology, Transcriptomics

## Abstract

*Xanthomonas fragariae* is a quarantine bacterial pathogen that causes angular leaf spot on strawberry. The aim of our study was to analyse the mechanism of interaction of this bacterium with its host plant at the transcriptome level. For this purpose, mRNAs of *X. fragariae* growing in Wilbrink’s medium and from infected strawberry cv. Elsanta plants were isolated and sequenced using the Illumina MiSeq platform. The expression profiles of the bacteria in Wilbrink’s medium and *in planta* were very diverse. Of the 3939 CDSs recorded, 1995 had significantly different expression *in planta* (966 and 1029 genes were down- and upregulated, respectively). Among the genes showing increased expression *in planta,* those with eggNOG/COG (evolutionary genealogy of genes: Non-supervised Orthologous Groups/Cluster of Orthologous Groups) categories associated with bacterial cell motility, signal transduction, transport and metabolism of inorganic ions and carbohydrates and transcription were overrepresented. Among the genes with the most increased expression *in planta,* genes primarily associated with flagella synthesis and chemotaxis were found. It is also interesting to note that out of the 31 genes localized on a plasmid, 16 were expressed differently *in planta*, which may indicate their potential role in plant–pathogen interactions. Many genes with differentiated expression that were localized on chromosome and plasmid encode proteins of unknown function.

## Introduction

*Xanthomonas fragariae* (*Xf*), the causal agent of angular leaf spot (ALS) of strawberries, is listed in Europe as a quarantine pathogen on the EPPO A2 list^1^. In the early phase of disease development, the symptoms of ALS are water-soaked angular spots visible on the abaxial leaf surface that enlarge over time, become reddish, necrotic and irregular and become visible on the adaxial leaf surface. When viewed with transmitted light, the spots are translucent. Under favourable conditions (high moisture and a temperature over 20 °C), a yellow bacterial exudate is produced on the leaf surface and calyces^2,3^.

The pathogen has a very narrow host range. It is found mostly on *Fragaria* × *ananassa* and incidentally on *F. virginiana*, *F. vesca* and two species of *Potentilla–P. fruticosa* and *P. glandulosa,* which show disease symptoms after artificial inoculation^1,2^*.* This limited host range in comparison to those of other plant pathogenic *Xanthomonas* species reflects a smaller genome (4.2 Mb vs. typical for this genus ~ 5 Mb) with a reduced repertoire of genes responsible for pathogenic abilities. On the other hand, the *Xf* genome reveals a high amount of DNA gained via horizontal gene transfer, which results in a set of uncommon genes possibly involved in pathogenicity^4^.

The pathogenic abilities of *Xanthomonas* bacteria are determined by several factors, and the major virulence-related gene regions are present in the *Xf* genome. *Xf* possesses a *hrp* gene cluster coding for structural elements of the type III secretion system (T3SS) and a distinct T3SS effector (T3E) set and an essential part of the *gum* cluster coding for xanthan—extracellular polysaccharide (EPS) synthesis^4^. It also contains a type IV secretion system (T4SS) and an *xps*-coded type II secretion system (T2SS) and, although in reduced form, TonB-dependent transporters, which do not play a direct role in pathogenesis but allow Gram-negative bacteria to take up scarce resources from nutrient-limiting environments by transporting molecules such as siderophores and hemes. *Xf* possesses the smallest repertoire of cell wall-degrading enzymes (CWDEs). In the *Xf* genome, there is a lack of xylan degradation and β-ketoadipate pathways commonly present in the majority of *Xanthomonas* pathogens. This can be related to the method and process of infection because *Xf* does not cause extensive tissue destruction as the necrotrophic pathogens with an abundance of CWDEs^5^.

Although *X. fragariae* is an important pathogen in the propagation of strawberry plant material^6,7^, there is limited knowledge of the molecular aspects of this plant pathogen. Generally, the genetic analysis of bacterial plant pathogen genes involved in pathogenic interactions with plants has been performed mostly through the production and screening of mutants, which usually allows the study of only a limited number of genes by “in vivo expression technology” (IVET)^8^. Other approaches, such as metabolomics, proteomics and DNA microarrays^9^, have several limitations. For instance, assigning an identity to the biomarkers can be problematic^10^, the detection limits may be high, and the possibility to simultaneously monitor bacterial activity may be limited^11^. By using next-generation sequencing, it is possible to characterize the whole transcriptome of pathogenic organisms, and this technique was successfully used for the study of both human and plant bacterial pathogens^12,13^.

The aim of our study was to determine the changes in gene expression of *X. fragariae* during infection of strawberry at the transcriptome level. For this purpose, we applied an RNA-seq technique to reveal the global changes in gene expression of *X. fragariae.* Until now, no detailed studies on the mechanism of action of this pathogen on hosts with different susceptibility levels have been carried out.

## Results

### Overview of RNA-seq results

**Figure 1:**
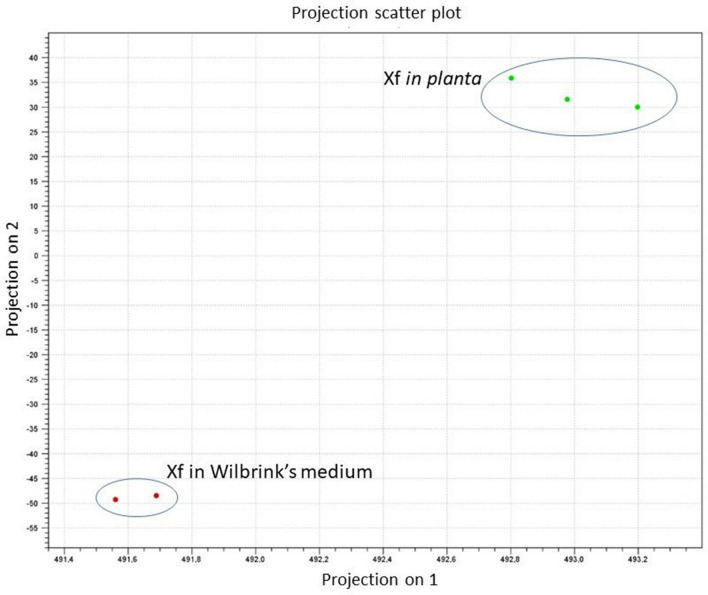
Principal component analysis (PCA) of the log_2_-transformed normalized expression values indicating the variation between *Xanthomonas fragariae* (*Xf*) grown overnight in liquid Wilbrink’s medium and *in planta* and between replicate samples at 15 days after inoculation.

For each biological replicate, a library was constructed and sequenced on a MiSeq sequencer (Illumina). For each sample, 4,770,496 to 6,554,258 reads were obtained, and 4,553,963 to 6,059,845 reads were mapped to the genome of *X. fragariae* IPO 3485 (Supplementary Table S1). The biological replicates showed a very high level of correlation (*r* ≥ 0.95). Principal component analysis (PCA) of the log_2_-transformed normalized expression values showed a large difference in values between the RNA expression of bacteria grown *in planta* and in liquid medium and small differences between replicate samples (Fig. 1).

**Figure 2:**
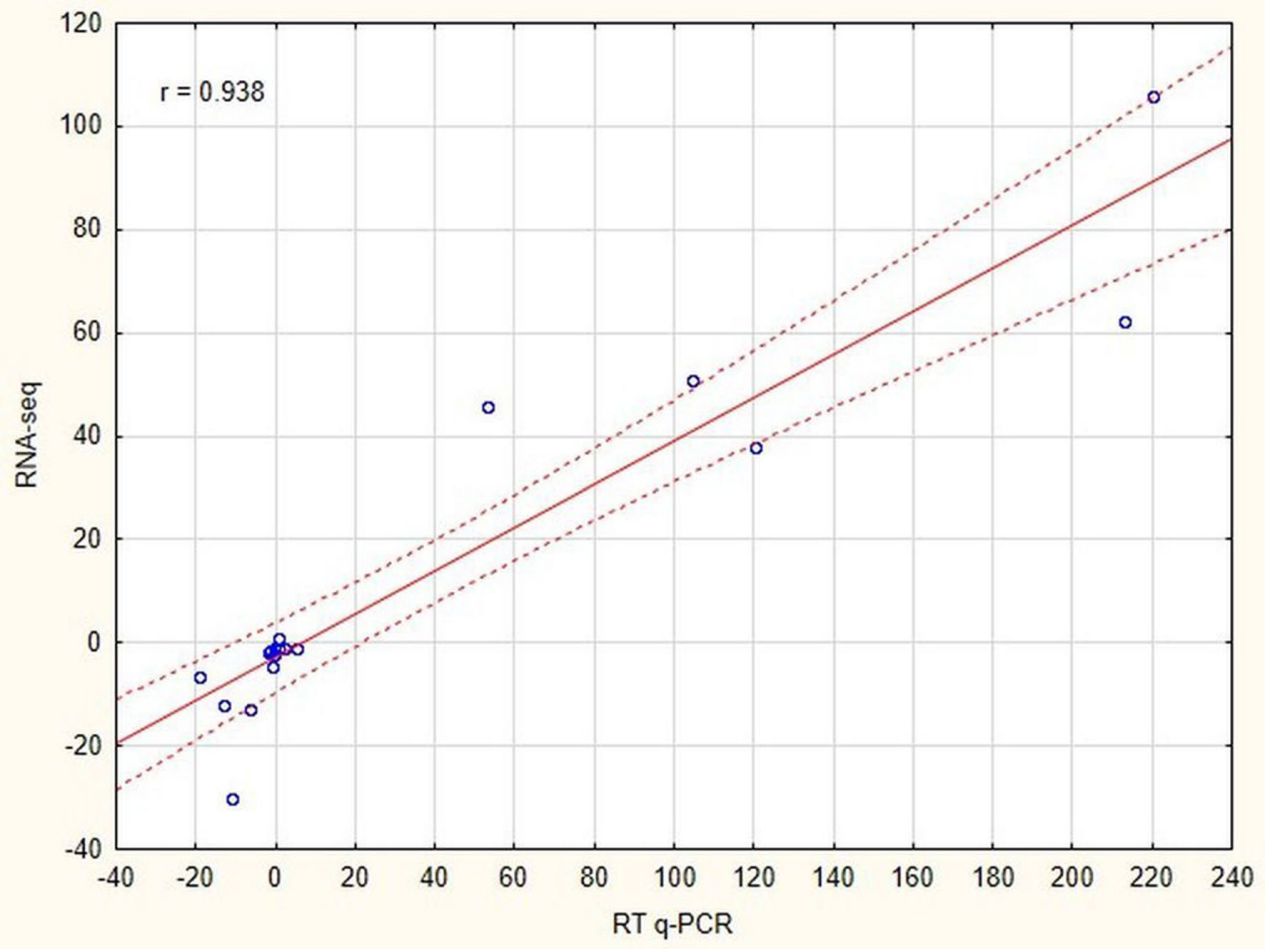
Validation of RNA-seq data using RT-qPCR. Fold changes in gene expression detected by RNA-seq are plotted against the RT-qPCR data. The reference line indicates a linear relationship between the results of RNA-seq and RT-qPCR. *r* Pearson correlation coefficient.

The accuracy of the RNA-seq data was verified by RT-qPCR (Reverse Transcribed quantitative Polymerase Chain Reaction). Fold changes in expression values under different environmental experimental conditions obtained with these two techniques were plotted on a scatter graph, with fold change values obtained from RT-qPCR on the *x*-axis and those obtained from RNA-seq on the *y*-axis (Fig. 2). The high value for the Pearson correlation coefficient (*r* = 0.938; *p* < 0.001;) indicated a positive correlation between the two techniques.

### Expression of *X. fragariae* IPO 3485 genes *in planta*

**Figure 3:**
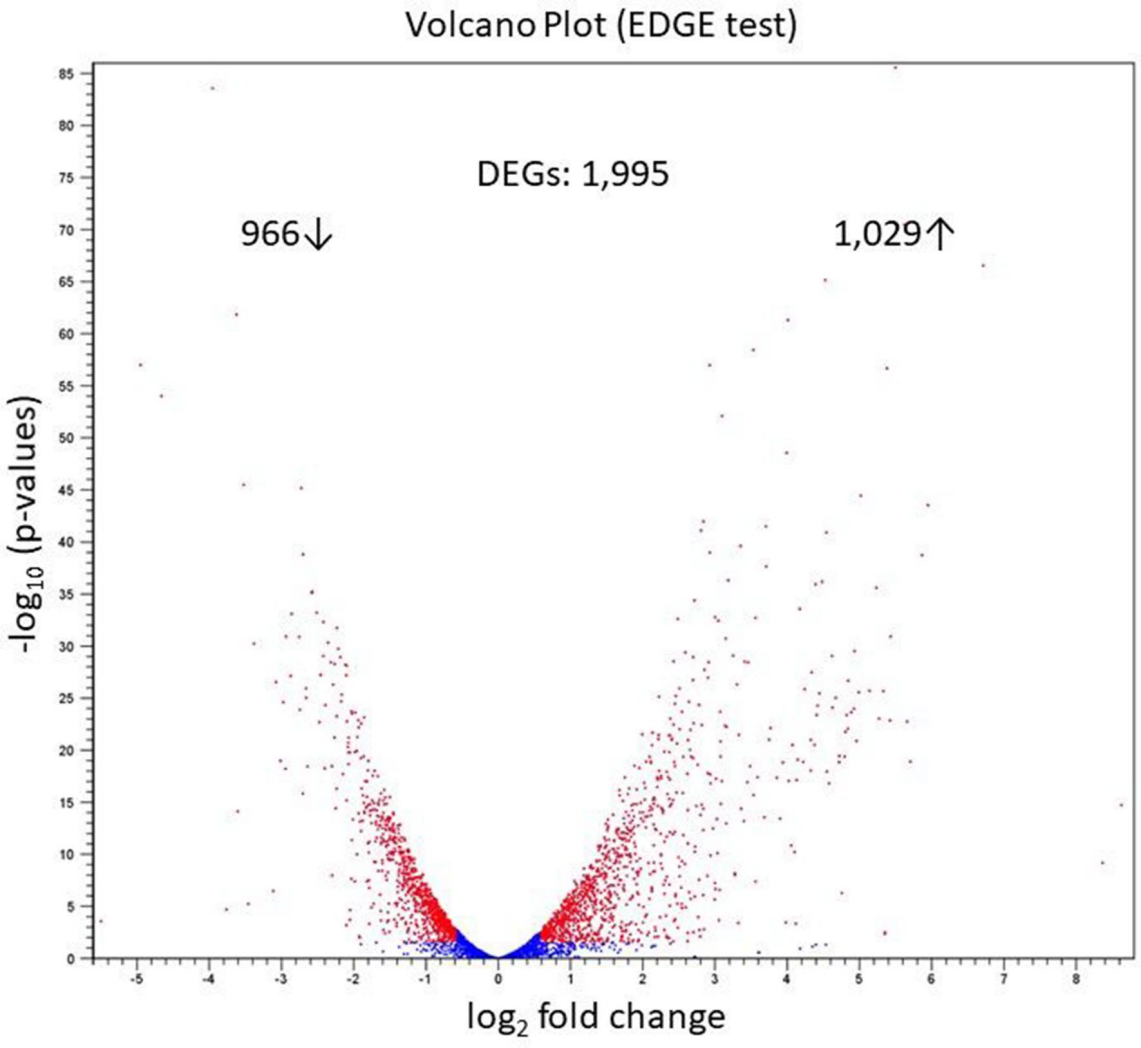
For every transcript, the fold change between bacteria growing in liquid Wilbrink’s medium and *in planta* 15 days after inoculation is plotted against the *p* value. Statistically significant differentially expressed genes (DEGs) with a log_2_ fold change ≥ 1.5 or ≤  − 1.5 are depicted as red dots, insignificant as blue dots. The numbers aside the arrow pointing up represent the number of upregulated genes and the numbers aside arrow pointing down represent the number of downregulated genes *in planta*.

Out of 3939 annotated CDSs on the genome of *X. fragariae* IPO 3485 strain, 1995 were differentially expressed *in planta* compared to bacteria in pure culture in liquid Wilbrink’s medium. Of all DEGs (Differentially Expressed Genes), 966 and 1029 were down- and upregulated *in planta*, respectively (Fig. 3, Supplementary Table S2).

The 1995 DEGs were classified into eggNOG/COG categories. In the case of downregulated genes, 7 of 25 eggNOG/COG categories were over-represented. The over-represented categories corresponding to the lowest *p* values in increasing order included energy production and conversion (C), lipid transport and metabolism (I), nucleotide transport and metabolism (F), amino acid transport and metabolism (E), translation (J), intracellular trafficking and secretion (U), and cell wall/membrane/envelope biogenesis (M). Among the 1029 upregulated genes, 7 eggNOG/COG categories were over-represented. The over-represented categories with the lowest *p* values in increasing order included the following: cell motility (N), signal transduction mechanisms (T), function unknown (S), posttranslational modification, protein turnover, chaperones (O), inorganic ion transport and metabolism (P), transcription (K), and carbohydrate transport and metabolism (G) (Supplementary Table S3).

The introduction of *X. fragariae* cells to strawberry leaf tissue also influenced the metabolic pathways—KEGG (Kyoto Encyclopedia of Genes and Genomes) pathways of the bacteria. Among the downregulated pathways, energy metabolism, nucleotide metabolism and translation were mostly over-represented. On the other hand, among the upregulated genes, over-representation of genes playing roles in the pathways of cell motility and signal transduction was observed (Supplementary Table S4).

Out of all *X. fragariae* genes located on the chromosome and a 21 kb plasmid, in all experimental combinations, the highest abundance of transcripts was observed for the gene NBC2815_00663, which was annotated to code the OmpA family outer membrane protein, followed by the genes NBC2815_00750, coding for OmpW family protein; *groL* (molecular chaperone GroEL); and NBC2815_02547, coding for a hypothetical protein. Proteins OmpA and OmpW are membrane proteins.

The most upregulated *X. fragariae* gene *in planta* was NBC2815_00636 (fold change (FCh) = 105.91), coding for the synthesis of the NADPH-sulfite reductase flavoprotein subunit. Among other highly upregulated genes, genes involved in flagella synthesis and chemotaxis and genes coding for hypothetical proteins were found (Supplementary Table S5). Of the downregulated genes, the gene NBC2815_03825, coding for the synthesis of phage-related capsid scaffold protein, was the most downregulated gene *in planta* (FCh =  − 44.97). Other highly downregulated genes included type IV secretion system genes, outer membrane adhesin protein XadA and a few genes coding for hypothetical proteins (Supplementary Table S6).

Among genes expected to be important for the pathogenicity of *X. fragariae,* different regulation of their expression *in planta* was observed^4^. The *gumBCDE* genes located in the *gum* cluster, encoding xanthan synthesis, were upregulated, while *gumKLMN* genes were downregulated. No changes in the expression of the nucleotide precursor of xanthan synthesis genes *xanA* and *xanB* (NBC2815_00824) were observed. Among the *rpf* genes known to be involved in the regulation of pathogenicity factors in other *Xanthomonas* species^14^, only *rpfA* was highly upregulated *in planta*, whereas *rpfF and rpfG* were downregulated and the rest of the *rpf* genes did not show any change in expression.

All genes involved in cell motility, including flagellar genes, were significantly upregulated. Out of 29 genes playing a role in chemotaxis, 26 revealed higher expression *in planta* than in pure culture*.* Among TonB-dependent transporters and receptors involved in the transport of e.g., siderophores, which are critical for successful niche settlement, different changes in expression were observed. Some of them, such as the gene *fhuA* or the genes NBC2815_01036 and NBC2815_02594, annotated as TonB-like proteins or receptors, were upregulated *in planta* (FCh = 3.99–6.97). On the other hand, the genes *fyuA* and *iroN_2*, coding for receptors, were significantly downregulated *in planta* (FCh <  − 8.48).

All but one (*hrpD6*) T3SS genes were upregulated *in planta*. The *hrpD6* gene also showed upregulation (FCh = 1.6), but the FDR (False Discovery Rate) was higher than 0.05. Among 40 genes annotated as T3SS or coding for avirulence proteins, 20 were upregulated, two (*xopAF* and NBC2815_00544) were downregulated, and the others showed no change in expression *in planta* (Supplementary Table S2)*.*

The genes of the *xps* cluster constituting a T2SS showed no change in expression or were downregulated *in planta*. The downregulated genes were *xpsD*, which codes for secretin (an outer membrane protein that, as a pentadecamer, provides a pore through the membrane), and the three genes *xpsIJK*, coding minor pseudopilins. Generally, T2SSs secrete toxins and degradative enzymes (such as xylanases, cellulases or proteases) that are transported across the inner bacterial membrane and often contribute to the virulence of plant pathogenic bacteria, e.g., by facilitating bacterial infection by degrading plant cell walls^15^. Although the role of T2SSs has not yet been studied in *Xf,* in the genome of *Xf,* several genes responsible for the synthesis of degradative enzymes, such as cellulases, proteases, polygalacturonases, lyases, amylases and lipases, were found. Many of them, such as all genes coding for amylases, polygalacturonases, and xylanase and the majority of genes coding for proteases, were upregulated *in planta* (Supplementary Table S7).

In the *X. fragariae* genome, genes coding for the type VI secretion system (T6SS) were found^4^. Of the T6SS genes coding for machinery proteins, increased expression of three genes, *impJ*, *tssJ* (NBC2815_01934) and *evpG* (NBC2815_01941), was observed. In the genome of *X. fragariae,* several copies of genes for T6SS effectors were found: 17 for VgrG, 15 for the Rhs protein family and one PAAR protein (proline–alanine–alanine–arginine repeat protein). Of them, 15 VgrG and 8 Rhs protein-coding genes were upregulated, while PAAR protein and 2 Rhs genes were downregulated.

*Xf* also possesses a whole cluster of a T4SS, which is responsible for intercellular DNA transfer, and almost all the genes were differentially expressed*.* Out of three *virD4* copies, only a *traG* copy located on the plasmid was upregulated, while two other copies on the chromosome were downregulated. Highly downregulated genes were involved in pilin synthesis: *pilW*, NBC2815_01578 and *pilE* (FCh > 12). A majority of *virB* genes were slightly upregulated. Type IV secretion systems have been shown to contribute to virulence, for instance, in *Xanthomonas campestris* pv. *campestris*^16^; however, its role in *Xf* pathogenicity is not known.

Only a limited change in the expression of multidrug efflux system genes was observed. Only three genes, NBC2815_00396, NBC2815_01613 and NBC2815_01676, were upregulated, and four, *norM*, NBC2815_00639, NBC2815_01397 and NBC2815_02129, were downregulated. Increased expression in strawberry plants was observed for three genes (NBC2815_01235, NBC2815_01669, NBC2815_02506) related to heat shock, which are primarily involved in defence against stress. Simultaneously, decreased expression was observed for two genes related to heat shock (NBC2815_01461, NBC2815_02622) and two related to cold shock (*scoF,* NBC2815_01420).

Differences in the expression of some genes coding for membrane proteins were detected between bacteria grown on medium and *in planta*. Membrane proteins create a selective barrier and protect bacteria from the environment by preventing entry of many toxic molecules into the cell; additionally, they are members of transport systems. Out of 126 genes annotated as coding for membrane proteins, differences in expression were observed for 58: 23 were upregulated, and 35 were downregulated in strawberry tissue (Supplementary Table S2). Among membrane protein genes with increased expression *in planta*, *tsr6* (coding for a protein chemotaxis sensory transducer) and *phuR_2* (coding for a hemin receptor) were the most upregulated, while NBC2815_03739 (coding for the adhesion protein XadA) and genes of proteins involved in LPS biosynthesis, WxcD, WxcE and WxcO, were the most downregulated *in planta*. The downregulation of LPS synthesis can be involved in the pathogen strategy to avoid inducing the immune response of plants because LPSs are recognized as pathogen-associated molecular patterns (PAMPs)^17^.

In addition to chromosomal DNA, strain IPO 3485 (NBC2815) possesses one 21.045 kb plasmid (pNBC2815-21). A total of 31 annotated genes are localized on this plasmid, and almost half of them have unknown functions. Of all plasmid genes, 17 were differentially expressed *in planta*: three were downregulated, and 14 were upregulated. The upregulated genes included *traG* (a member of the T4SS) and genes coding for recombinases and partitioning proteins. On the plasmid, genes potentially involved in pathogenicity, such as *xopT* (T3E) and NBC2815_04017, encode avirulent proteins, but their expression was not changed *in planta.*

Cyclic-di-GMP (c-di-GMP), a universal bacterial secondary messenger signalling compound, regulates different aspects of cell life, including processes involved in effective pathogenicity and responses to a variety of environmental stimuli, including stress. The effects of c-di-GMP are dose dependent, and their level is controlled by the opposing actions of diguanylate cyclases (DGC), including GGDEF domain proteins, and phosphodiesterases (PDEs), possessing EAL or HD-GYP domains. These two groups of enzymes control the synthesis and degradation of c-di-GMP, respectively^18^. In the genome of *Xf* IPO 3485, 10 genes possessing these characteristic domains were annotated, and eight of them (NBC2815_01280, NBC2815_01823, NBC2815_02702, NBC2815_03946, NBC2815_01824, NBC2815_01207, NBC2815_00405, NBC2815_00406) were upregulated *in planta*.

## Discussion

*X. fragariae* is a rather weak pathogen with a relatively slow growth rate, a limited possibility of systematically colonizing plants, and a restricted capacity to survive outside plant tissue^19^. Moreover, the pathogen survives poorly on leaf surfaces, and a significant decrease in the population density in the first 3 days after inoculation was found^6^. This drop in the population can indicate that this pathogen uses a strategy of avoidance^20^ to escape environmental stresses and enters the plant leaves to survive. The population gradually rebuilds during later phases of infection, reaching the preparatory stage of the bacteria before the exudation phase at ca. 14 days post inoculation^6^. During disease development, symptoms start from single small spots of water-soaked lesions bounded by leaf vines. With time, the number of angular spots increases, and as observed by electron microscopy techniques^3^, bacterial cells suspended in copious amounts of EPS colonize mesophyll air spaces. In the next steps, plasmolysis of mesophyll cells and disruption of organelles occur, and bacteria ooze from stomates. In our study, we analysed *Xf* mRNA *in planta* with RNA-seq 15 days after inoculation, when well-developed water soaking symptoms were observed but strawberry tissue was not necrotized yet. We could assume that the bacterial cell stages and gene expression of the cells that were located in the water-soaked leaf parts and on the border of healthy and already diseased plant tissue were different. The bacterial population may be localized in the intercellular spaces and partially move into the vascular tissue; however, a large part is in degraded tissue. The obtained results of transcriptome analysis are thus an average expression of cells in different steps of interaction with plant tissue.

In all experimental combinations, the highest abundance of transcripts was observed for the genes coding membrane proteins OmpA and OmpW, *groL* (encoding molecular chaperone GroEL), and one gene of a hypothetical protein. The high number of transcripts of the gene coding OmpA can be caused by the fact that it is the most abundant outer membrane (OM) protein in many bacterial species, e.g., OmpA in *Escherichia coli* is present at 100,000 copies per cell^21^. Although this protein is also found to play a role in the pathogenesis of, e.g., *Xanthomonas albilineans*^22^*,* its expression was lower *in planta* than in Wilbrink’s medium, similar to the *groL* gene behaviour. Chaperone GroEL is involved in protein folding and cell proliferation/survival^23^. OmpW is involved in the transport of small hydrophilic molecules across the bacterial outer membrane^24^. Studies in *Salmonella typhimurium* suggest that this porin may have a role in the protection of bacteria against various forms of environmental stress by operating as an efflux channel for toxic compounds^25^, and in the case of *Xanthomonas axonopodis* pv. *citri*, OmpW is suspected to be involved in protecting biofilms^26^.

The T6SS system is widespread in Gram-negative bacteria and can deliver toxic effector proteins into eukaryotic cells or competing bacteria; it can also participate in uptake of metal ions, such as iron, manganese, and zinc. T6SS effector translocation apparatuses are very similar to an inverted bacteriophage-puncturing device composed of at least 13 proteins identified as core components^27,28^. In our study, three genes coding for the elements of this translocation apparatus and many coding for T6SS effectors were upregulated *in planta*, which suggests their importance in relation to host plants; however, the potential role of this system in the pathogenic relation of *Xf* is unknown.

Among the upregulated genes *in planta,* seven out of 25 eggNOG/COG categories were over-represented. Although the *in planta Xf* gene expression was analysed 15 days after strawberry leaf inoculation, the groups of upregulated *Xf* genes are congruent with those of *Xanthomonas oryzae* pv*. oryzae* (*Xoo*) in an in vitro system with rice leaf extract during the first 60 min after contact with the medium^29^. However, taking into account the low virulence of *Xf* and problems with maintaining a high population in early contact with plants, it is difficult to compare what time since inoculation can be equivalent to the first minutes of contact in the case of *Xoo.* In *Xoo,* the most over-represented categories among the differentially expressed genes were inorganic ion transport and metabolism (P) and cell motility (N). Uptake of inorganic ions, such as iron, seems to be crucial for plant pathogenic bacteria that need to compete with their host to obtain nutrients. The key regulator HrpG is a repressor of chemotaxis and flagella biosynthesis-related genes and consequent bacterial motility^30^, which was confirmed in a study on *Xoo*^29^*.* In the case of our study, motility, chemotaxis and flagella synthesis genes were all significantly upregulated together with high upregulation of *hrpG* and other hrp genes, which again can represent the average gene expression of *Xf* cells in the leaves.

Pili are involved in twitching motility and may be involved in the adhesion of *Xanthomonas* species^31^. The genes coding for the synthesis of pili are downregulated in *Xf* present in water-soaked lesions, possibly because the process of attachment to the host had already passed. Conversely, the genes for flagella synthesis were upregulated. For *X. axonopodis* pv. *citri,* it has been demonstrated that flagella and flagella-dependent motility are important not only for adherence to biotic and abiotic surfaces but also for maturing of the biofilm, symptom development and extension and dispersion of the biofilms^32^, indicating that maturation and extension of the biofilm in the lesions still takes place. The upregulation of chemotaxis and flagella genes may also indicate a preparation for a release and dispersal of the pathogen via moisture from the lesions^33^.

Looking at the expression of genes known as important for pathogenicity, out of 12 genes of xanthan synthesis, only four *gumBCDE* were upregulated, the next four genes in the operon showed no change in expression, and the last four genes were downregulated. In xanthan biosynthesis, the upregulated *gumBCE* genes are responsible for polymerization and/or export of the polymer, while the others are mostly responsible for the synthesis^34^, so it seems that at this stage of plant infection, synthesis of xanthan was not relatively intensive. Out of the *rpf* gene cluster, which is known to be involved in the regulation of pathogenicity factors, an increase in expression was observed only for the *rpfA* gene. Mutation of this gene resulted in reduced levels of extracellular enzymes and EPS in *Xanthomonas campestris* pv. *campestris* (Xcc). Additionally, a possible connection between *rpfA* and iron metabolism was suggested^35^. Simultaneously, no change or decrease in expression was recorded for two other genes, *rpfB* and *rpfF,* which are essential for the synthesis of a small diffusible signal molecule that is involved in the regulation of virulence factor synthesis in response to physiological or environmental changes^36^.

*Xf* has a distinct T3E repertoire comprising multiple rare effectors and several putative new effectors^4^. Of genes annotated T3E or coding for avirulence proteins, only some were upregulated *in planta*. Among the five genes annotated *xopP*, only one *xopP_1* had higher expression *in planta* than in pure culture*,* while four other copies showed no change in expression. XopP was found to be a crucial T3E in *X. campestris*^37^, conserved among different pathovars of this species and other *Xanthomonas* species^38^. Its function was recently described as blocking peptidoglycan- and chitin-triggered immunity in rice by inhibiting the U-box ubiquitin ligase OsPUB44^39^. All five copies are located in one region of the genome next to each other. Interestingly, several putative new effectors found by Vandroemme et al*.*^4^ in the *Xf* genome were differentially expressed in strawberry plants. Effectors of foreign origin (such as XopC1) with the highest homology to proteins in *Ralstonia* (e.g., XopAD_1, which shows the highest similarity to an unidentified protein in *Mesorhizobium*) were upregulated *in planta*, similar to the majority of genes coding putative XopD and showing distant homology to a T3E of *Pseudomonas.* These results suggest that the foreign origin genes coding for T3E can be functional in *Xf* during the pathogenesis process.

The role of T2SS, used for secretion of degradative enzymes, has not yet been studied in *X. fragariae.* In other *Xanthomonas* species, such as *X. campestris* pv. *vesicatoria*^40^ and *X. axonopodis* pv. *citri*^41^, the xps cluster constituting the T2SS has been identified. It is likely that this secretion system also plays a role in *Xf*, as several genes responsible for the synthesis of degradative enzymes, such as cellulases, proteases, polygalacturonases, lyases, amylases and lipases, were upregulated *in planta.*

During infection of plants, bacteria are exposed to a variety of antimicrobial compounds produced by the host. One of the mechanisms underlying plant resistance to diseases can be related to increased concentrations of toxic pathogen compounds, as is found in apple cultivars resistant to *Erwinia amylovora*^13^*.* Multidrug efflux pumps and permeases recognize and efficiently expel a wide range of structurally diverse compounds from the bacterial cell and play a very important role in the success of pathogens^42^. This observation could suggest that the compounds produced by strawberry leaf tissue are not toxic for *Xf* although they have antimicrobial properties^43^, and therefore, the expression of genes coding for proteins involved in the detoxification of bacterial cells is not increased *in planta*.

Plasmids are the extrachromosomal elements widely spread in plant pathogenic bacteria. The range of plasmid-coded features, including virulence, toxin and hormone production and resistance to bactericides, has been reported in many phytopathogenic bacteria. In the case of *Xanthomonas,* plasmid biology is still not well understood^44^; however, there are some reports on organisms such as *X. campestris* pv. *malvacearum* showing that in cotton, a number of plasmid-borne *avr* genes together encode the ability to cause watersoaking in the host plant^45^. The role of plasmids in *X. fragariae* has not been studied at all; however, the presence of a gene coding for T3E on the plasmid and the change in expression of almost half of the plasmid genes *in planta* suggest its potential importance in the interaction with plants.

## Conclusions

The presented RNA-seq analysis describes the transcriptional response of *X. fragariae* in strawberry cv. Elsanta leaves 15 days after inoculation. The majority of genes known to be important for pathogenicity had increased expression *in planta.* Among genes with the greatest fold change in expression between experimental conditions or with the highest transcript abundance, there are many genes not assigned functions that have never been tested for their role in pathogenicity. Thus, although their role is unknown, their function in interacting with the host plant may be important. This study provides the first transcriptional profile by RNA-seq of *X. fragariae* during infection of a host plant.

## Methods

### Sample collection and RNA isolation

Strain *X. fragariae* IPO (Instituut voor Plantenziektenkundig Onderzoek) 3485, isolated from an infected strawberry plant in 2011 in the Netherlands, was used in this study. For RNA isolation, young leaves of strawberry cv. Elsanta grown in a quarantine greenhouse (25–30 °C; relative humidity level of 70–90%, natural light conditions) were inoculated with a water bacterial suspension of 10^6^ cfu mL^−1^ grown for 20 h in liquid Wilbrink’s medium^46^ by high-pressure spraying of the abaxial and adaxial surfaces of leaves as previously described^47^.

Fifteen days after inoculation, samples were processed, and total RNA was isolated using a Total RNA Purification Kit (Norgen Biotek) after the Genomic Mini AX SOIL Spin kit (A&A Biotechnology, Gdynia, Poland) as described by Kałużna et al.^47^. At this time point, RNA was isolated separately from three samples of 1.5 to 2.5 g of water-soaked parts of strawberry leaves. Before isolation, the surface of the leaves was sterilized^6^. Additionally, RNA was isolated from a pure culture of *X. fragariae* IPO 3485 grown at 26 °C with 150 rpm shaking for 20 h in liquid Wilbrink’s medium. DNA was removed from samples by DNase treatment (Deoxyribonuclease I, ThermoScientific, Lithuania). The efficiency of DNA removal was tested by PCR with the primers 245A and 245B^48^, which were designed for the detection and identification of *X. fragariae.* Determination of the quality and concentration of obtained DNA-free RNA was tested on an Agilent 2100 Bioanalyzer using the Agilent RNA 6000 Nano Kit according to the manufacturer’s instructions. Two samples of pure bacterial culture and three samples of RNA isolated from infected strawberry leaves of the best quality (RIN) were subjected to rRNA depletion using a Ribo-Zero Magnetic Kit (Gram-Negative Bacteria, Illumina); they constituted biological replicates for each experimental combination.

### Library preparation and sequencing

The rRNA-depleted sample concentration was measured using a 2100 Bioanalyzer (Agilent) and an RNA 6000 Pico Kit (Agilent, 5067-1513). The maximum allowable volume (6 µl) of mRNA was used for library construction using the NEBNext Ultra Directional RNA Library Preparation Kit for Illumina (New England Biolabs, E7420S). The libraries were sequenced on a MiSeq (Illumina) using the MiSeq Reagent Kit v2 (500 cycles) (Illumina, MS-102-2003) in the PE250 read mode. The resulting reads were additionally trimmed with Cutadapt v. 1.12 at default parameters^49^ These sequence data have been submitted to the ArrayExpress (EMBL) databases under accession number E-MTAB-8189.

### Bioinformatic analysis

Low-quality sequence ends (ambiguous base limit: 2, quality limit: 0.05) were trimmed using the CLC Genomics Workbench v. 8.1 (Qiagen) Trim Sequences tool. High-quality sequences were aligned to the *X. fragariae* strain IPO 3485 (= NBC 2815) genome (LT853880, LT853881)^50^ using the CLC RNA-seq reference mapping algorithm with settings appropriate for prokaryotic genomes (mapping to gene regions only). A quality control to check whether the overall variability of the samples reflected their grouping and the reproducibility between repetitions was performed with PCA. For DEGs analysis, expression values were normalized using the Trimmed Mean of M values (TMM)^51^, and DEGs were analysed using the empirical analysis of DGE tool based on Exact Test incorporated in the EdgeR Bioconductor package and implemented in CLC Genomics Workbench. A gene was considered to be differentially regulated between two conditions when the gene showed a total read number larger than 5, a > 1.5-fold absolute fold-change ratio and an FDR-adjusted *p* value < 0.05.

To summarize the pathway information protein sequence, fasta files were submitted to KAAS (KEGG automatic annotation server)^52,53^, and KEGG orthology assignments were obtained (Supplementary Table S8). The eggNOG 4.5 database^53^ was used to annotate genes with common denominators or functional categories (i.e., derived from the original COG categories). Enrichment of COG and KEGG terms was evaluated by a hypergeometric distribution at FDR < 0.05.

### RT-qPCR validation

The transcription expression reported in the present study was validated through RT-qPCR using 18 candidate genes selected from highly and lowly expressed bacterial genes with newly designed primers (Supplementary Table S2). Herein, three biological replicates were used to evaluate the transcriptional expression of the *X. fragariae* strain IPO 3485 in each biological replicate. For gene amplification, total RNA was isolated, reverse transcribed and amplified with real-time PCR, as described by Kałużna et al.^47^. The qPCR runs were performed on a Bio-Rad CFX96 thermocycler with SsoAdvanced SYBR Green Supermix (Bio-Rad, Hercules, CA) under the conditions described^47^ using the comparative 2 − ΔΔC_T_ method.

The genes with the most stable expression based on previous analysis^47^ were used for normalization of RT-qPCR expression analysis of tested genes: *gyrB* (DNA gyrase B)*, ffh* (signal recognition particle protein)*,* and *pykA* (pyruvate kinase). For assessment of the association between RNA-seq and RT-qPCR, Pearson's correlation method was used.

### Ethics approval and consent to participate

Plant material was bought from the commercial nursery. No field permissions were necessary to collect the plant samples. No specimens have been deposited as vouchers. Research Institute of Horticulture possess a permission of Main Inspectorate of Plant Health and Seed Inspection, Poland for work with quarantine pathogen *Xanthomonas fragariae* (Decision No. WF-411d-6/14).

## Supplementary information

Supplementary Information 1.
