# Supplementary material for: Transcriptome analysis of Xanthomonas fragariae in strawberry leaves
Source: Sci Rep. 2020 Nov 25;10:20582. doi: 10.1038/s41598-020-77612-y (PMC7688646; doi:10.1038/s41598-020-77612-y)
Supplement: Supplementary file 1 — Supplementary Information 1. [file 41598_2020_77612_MOESM1_ESM.pdf]

Supplementary Information

Transcriptome analysis of *Xanthomonas fragariae* in strawberry leaves

Joanna Puławska[1,*], **Monika Kałużna**[1], **Wojciech Warabieda**[1], **Joël F. Pothier** [2], **Michael Gétaz** [2], **Jan M. van der Wolf** [3]

[1] Research Institute of Horticulture, Department of Phytopathology, 96-100 Skierniewice, Poland

[2] Environmental Genomics and Systems Biology Research Group, Institute for Natural Resource Sciences, Zurich University of Applied Sciences (ZHAW), Wädenswil, Switzerland

[3] Wageningen University & Research, Wageningen, The Netherlands

*Corresponding author: joanna.pulawska@inhort.pl

Table S1. Metrics of RNA-seq data.

Sample	Total reads	Number of mapped reads to *Xf* genome[1]	% of mapped reads to *Xf* genome	Unmapped reads to *Xf* genome	% of reads aligned to rRNA operon	Mapping to genes[2]	
						Mean read length (bp)	Total read length (bp)
Xf-bact-1	4,770,496	4,553,963	95.46	216,533	0.53	162.19	738,586,546
Xf-bact-2	6,399,558	6,059,845	94.69	339,713	6.37	161.69	989,760,152
Xf-*in planta*-1	5,625,304	5,091,623	90.51	800,879	0.32	165.28	854,782,067
Xf-*in planta*-2	6,121,370	5,500,747	89.86	719,953	0.40	152.42	848,151,476
Xf-*in planta*-3	6,554,258	5,835,096	89.03	719,162	0.31	177.34	1,049,304,221

[1] Genbank accession numbers LT853880 and LT853881

[2] Used in RNA-seq analysis.

Table S2. Differentially expressed genes of *X. fragariae* IPO 3485 genes between Wilbrink's medium and 15 days after inoculation of strawberry cv. Elsanta leaves

Gene	Locus tag in IPO 3485 genome	Fold change*	FDR *p* -value correction	Product
NBC2815_00002	NBC2815_00002	-1.55	1.07E-02	DNA polymerase III subunit beta /EC_number="2.7.7.7
NBC2815_00003	NBC2815_00003	-2.58	6.25E-08	transposase
NBC2815_00004	NBC2815_00004	-1.89	2.84E-02	transposase-like protein
NBC2815_00010	NBC2815_00010	-1.72	4.30E-03	TonB protein
exbB	NBC2815_00011	-2.18	1.90E-05	biopolymer transport protein
NBC2815_00012	NBC2815_00012	-2.01	2.59E-04	biopolymer transport ExbD1 protein
NBC2815_00013	NBC2815_00013	-1.97	2.08E-04	biopolymer transport ExbD protein
NBC2815_00014	NBC2815_00014	2.27	3.26E-05	hypothetical protein
NBC2815_00016	NBC2815_00016	3.88	8.51E-11	radical SAM domain-containing protein
NBC2815_00017	NBC2815_00017	2.14	1.15E-06	cardiolipin synthetase
NBC2815_00018	NBC2815_00018	-1.92	5.06E-04	Hypothetical Protein
NBC2815_00021	NBC2815_00021	-2.85	2.11E-09	outer membrane protein
NBC2815_00023	NBC2815_00023	-2.77	3.58E-09	hypothetical protein
NBC2815_00024	NBC2815_00024	-1.61	6.88E-03	D-3-phosphoglycerate dehydrogenase
ctp	NBC2815_00025	-1.85	4.49E-04	carboxyl-terminal protease
NBC2815_00029	NBC2815_00029	3.82	2.12E-09	peptidase propeptide and ypeb domain-containing protein
NBC2815_00031	NBC2815_00031	2.06	5.70E-06	glycolate oxidase /EC_number="1.1.1.28
trn2	NBC2815_00033	-1.55	4.16E-03	tropinone reductase
NBC2815_00034	NBC2815_00034	-2.44	9.08E-07	Hypothetical Protein
sppA	NBC2815_00035	-1.74	1.31E-03	endopeptidase IV
norM	NBC2815_00036	-1.51	1.01E-02	multidrug efflux protein
priA	NBC2815_00039	2.14	8.33E-07	primosome assembly protein PriA
NBC2815_00040	NBC2815_00040	4.49	2.78E-09	hypothetical protein
rpoH	NBC2815_00041	2.28	5.57E-06	RNA polymerase factor sigma-32
NBC2815_00042	NBC2815_00042	-2.50	3.20E-09	uracil-DNA glycosylase /EC_number="3.2.2.-
NBC2815_00043	NBC2815_00043	-1.57	1.36E-03	response regulator protein
NBC2815_00048	NBC2815_00048	-1.55	1.25E-02	hypothetical protein
NBC2815_00051	NBC2815_00051	2.29	2.66E-06	swim zinc finger domain protein
NBC2815_00052	NBC2815_00052	1.67	3.40E-02	hypothetical protein
NBC2815_00053	NBC2815_00053	1.73	1.21E-02	Hypothetical Protein
queF	NBC2815_00054	2.07	1.99E-06	7-cyano-7-deazaguanine reductase
NBC2815_00059	NBC2815_00059	-1.85	4.30E-03	hypothetical protein
NBC2815_00061	NBC2815_00061	-3.21	9.86E-17	hypothetical protein
NBC2815_00065	NBC2815_00065	-2.06	7.92E-06	periplasmic glucan biosynthesis protein
NBC2815_00068	NBC2815_00068	1.69	2.86E-03	hypothetical protein
NBC2815_00069	NBC2815_00069	-1.83	1.11E-04	Hypothetical Protein
NBC2815_00070	NBC2815_00070	-2.03	1.01E-05	acid phosphatase
NBC2815_00075	NBC2815_00075	-1.69	1.69E-03	Hypothetical Protein
plsB	NBC2815_00076	-1.58	6.52E-03	glycerol-3-phosphate acyltransferase
NBC2815_00077	NBC2815_00077	3.15	2.91E-13	nuclear receptor-binding factor-like protein
NBC2815_00078	NBC2815_00078	-2.09	6.38E-07	Hypothetical Protein
NBC2815_00080	NBC2815_00080	-1.69	1.03E-03	prophage Lp2 protein 6
NBC2815_00082	NBC2815_00082	-1.72	1.96E-03	IS1478 transposase
NBC2815_00084	NBC2815_00084	10.10	1.34E-03	glucan 1,4-beta-glucosidase
aguA_1	NBC2815_00085	3.80	2.78E-03	alpha-glucuronidase
aguA_2	NBC2815_00086	2.65	1.65E-02	alpha-glucuronidase
NBC2815_00087	NBC2815_00087	1.92	2.84E-05	sal operon transcriptional repressor
rdgC	NBC2815_00089	-1.87	3.99E-05	recombination associated protein
hemH	NBC2815_00092	-1.81	3.22E-05	ferrochelatase
NBC2815_00097	NBC2815_00097	2.16	1.14E-04	membrane protein
guaA_1	NBC2815_00098	4.70	9.52E-21	glutamine amidotransferase
NBC2815_00099	NBC2815_00099	1.98	3.06E-05	type II secretory pathway ATPase
glyQ	NBC2815_00100	-2.38	5.95E-10	glycyl-tRNA synthetase subunit alpha
glyS	NBC2815_00101	-2.47	7.53E-10	glycyl-tRNA synthetase subunit beta
NBC2815_00102	NBC2815_00102	-1.61	8.67E-04	Hypothetical Protein
gltB	NBC2815_00103	2.35	1.20E-06	glutamate synthase subunit alpha
NBC2815_00104	NBC2815_00104	2.65	1.60E-08	glutamate synthase
NBC2815_00105	NBC2815_00105	1.60	1.81E-02	alcohol dehydrogenase
NBC2815_00106	NBC2815_00106	1.52	8.85E-03	cellulase
NBC2815_00107	NBC2815_00107	5.19	1.05E-20	cellulase
NBC2815_00109	NBC2815_00109	1.81	1.45E-03	tis1421-transposase b
NBC2815_00110	NBC2815_00110	1.94	1.49E-02	IS1481 transposase
xopP_1	NBC2815_00112	1.64	7.77E-04	type III effector protein XopP
NBC2815_00117	NBC2815_00117	1.83	9.61E-05	hypothetical protein
NBC2815_00119	NBC2815_00119	1.82	2.53E-03	alpha-amylase
glgB1	NBC2815_00121	1.64	1.06E-02	glycogen branching protein
NBC2815_00122	NBC2815_00122	2.32	6.17E-07	oxidoreductase
NBC2815_00123	NBC2815_00123	3.12	7.34E-09	carboxylesterase type B
NBC2815_00124	NBC2815_00124	1.83	2.20E-02	putative secreted esterase/lipase precursor
NBC2815_00126	NBC2815_00126	5.51	1.51E-16	C4-dicarboxylate transport system
NBC2815_00127	NBC2815_00127	3.08	4.41E-07	C4-dicarboxylate transport protein
xylB1	NBC2815_00128	3.86	1.98E-07	xylosidase precursor
NBC2815_00129	NBC2815_00129	-1.53	1.49E-02	LacI family transcriptional regulator
NBC2815_00132	NBC2815_00132	2.94	5.05E-06	5-keto-4-deoxyuronate isomerase /EC_number="5.3.1.17
phhA	NBC2815_00135	-3.73	1.35E-12	phenylalanine 4-monooxygenase
NBC2815_00137	NBC2815_00137	-2.21	2.92E-06	ABC transporter ATP-binding protein
NBC2815_00138	NBC2815_00138	2.86	3.27E-04	EF hand domain-containing protein
NBC2815_00139	NBC2815_00139	4.24	4.40E-13	ABC transporter ATP-binding protein
NBC2815_00140	NBC2815_00140	3.56	5.75E-12	ABC transporter amino acid permease
NBC2815_00141	NBC2815_00141	2.07	9.08E-05	class V aminotransferase
NBC2815_00145	NBC2815_00145	1.54	1.54E-02	indolepyruvate ferredoxin oxidoreductase
NBC2815_00147	NBC2815_00147	1.60	5.10E-03	permease
parA	NBC2815_00148	2.60	2.18E-08	partition protein
NBC2815_00149	NBC2815_00149	2.60	2.68E-09	putative phosphoglycerate mutase family protein
NBC2815_00152	NBC2815_00152	-1.66	3.13E-03	esterase YdiI
NBC2815_00157	NBC2815_00157	4.13	7.63E-19	tis1421-transposase b
NBC2815_00159	NBC2815_00159	1.64	1.76E-02	superoxide dismutase-like protein
NBC2815_00161	NBC2815_00161	1.56	4.80E-03	two-component system sensor protein
uppP	NBC2815_00165	3.51	6.93E-12	UDP pyrophosphate phosphatase
NBC2815_00166	NBC2815_00166	5.60	6.76E-15	Hypothetical Protein

NBC2815_00172	NBC2815_00172	1.96	1.02E-05	Hypothetical Protein
hemB	NBC2815_00174	-1.58	1.96E-03	delta-aminolevulinic acid dehydratase
aroE	NBC2815_00177	-2.32	1.53E-09	shikimate 5-dehydrogenase
NBC2815_00180	NBC2815_00180	30.92	4.57E-29	catalase
NBC2815_00181	NBC2815_00181	42.12	1.39E-56	ankyrin-like protein
NBC2815_00182	NBC2815_00182	1.94	2.56E-03	IS1478 transposase
NBC2815_00185	NBC2815_00185	3.06	5.23E-03	ABC transporter ATP-binding protein
NBC2815_00186	NBC2815_00186	4.32	1.86E-05	ABC transporter permease
NBC2815_00187	NBC2815_00187	1.95	5.93E-04	hydrolase
trpS	NBC2815_00192	-1.50	6.82E-03	tryptophanyl-tRNA synthetase
argI	NBC2815_00196	-1.96	7.00E-04	arginase
NBC2815_00202	NBC2815_00202	1.65	1.48E-03	cytochrome B561
NBC2815_00203	NBC2815_00203	1.87	3.75E-04	catalase
NBC2815_00207	NBC2815_00207	2.65	3.22E-07	hypothetical protein
NBC2815_00208	NBC2815_00208	3.09	2.82E-10	Hypothetical Protein
NBC2815_00209	NBC2815_00209	2.32	1.58E-06	Hypothetical Protein
NBC2815_00210	NBC2815_00210	1.59	1.01E-02	Hypothetical Protein
NBC2815_00211	NBC2815_00211	1.97	4.79E-04	protease Do
bpH1	NBC2815_00212	-1.50	2.98E-02	histone H1-like protein
NBC2815_00213	NBC2815_00213	4.51	5.78E-09	secreted protein
NBC2815_00214	NBC2815_00214	26.67	2.67E-17	Hypothetical Protein
ygiY	NBC2815_00215	2.24	6.20E-06	two-component system sensor protein
NBC2815_00216	NBC2815_00216	2.16	2.02E-05	two-component system regulatory protein
NBC2815_00219	NBC2815_00219	2.80	1.16E-11	cell division inhibitor
NBC2815_00220	NBC2815_00220	3.07	2.53E-12	Hypothetical Protein
NBC2815_00221	NBC2815_00221	4.18	6.94E-19	Hypothetical Protein
NBC2815_00222	NBC2815_00222	3.19	6.46E-12	Hypothetical Protein
NBC2815_00223	NBC2815_00223	3.19	1.10E-09	membrane protein
NBC2815_00224	NBC2815_00224	3.25	3.14E-03	hydrolase or peptidase
NBC2815_00225	NBC2815_00225	1.93	5.23E-04	Hypothetical Protein
NBC2815_00226	NBC2815_00226	1.93	1.48E-05	IS1480 transposase
NBC2815_00227	NBC2815_00227	2.04	2.57E-05	Hypothetical Protein
NBC2815_00228	NBC2815_00228	1.55	1.09E-02	Hypothetical Protein
NBC2815_00231	NBC2815_00231	-1.86	6.36E-04	Fatty acid desaturase
NBC2815_00232	NBC2815_00232	1.87	3.86E-05	signal transduction protein
NBC2815_00233	NBC2815_00233	3.93	5.33E-14	Hypothetical Protein
NBC2815_00234	NBC2815_00234	2.77	5.47E-10	outer membrane lipoprotein Blc
NBC2815_00236	NBC2815_00236	-2.74	8.39E-10	Hypothetical Protein
NBC2815_00237	NBC2815_00237	-2.29	6.28E-04	nitrogen regulatory protein P-II
NBC2815_00240	NBC2815_00240	-1.90	2.33E-03	Hypothetical Protein
NBC2815_00243	NBC2815_00243	-1.81	1.03E-05	arginine decarboxylase /EC_number="4.1.1.19
NBC2815_00244	NBC2815_00244	-3.51	8.90E-08	ISXo1 transposase, IS5 family
NBC2815_00248	NBC2815_00248	-1.74	6.10E-05	DNA polymerase I
NBC2815_00249	NBC2815_00249	-1.50	1.20E-02	coproporphyrinogen III oxidase /EC_number="1.3.3.3
NBC2815_00252	NBC2815_00252	2.20	5.46E-05	Hypothetical Protein
NBC2815_00253	NBC2815_00253	-2.15	2.93E-06	aminopeptidase /EC_number="3.4.-.-
NBC2815_00254	NBC2815_00254	-1.53	9.29E-03	AMP-ligase
NBC2815_00255	NBC2815_00255	-1.65	1.07E-03	pteridine-dependent deoxygenase like protein
fixC	NBC2815_00256	-1.66	1.99E-04	Dehydrogenase (flavoproteins)
NBC2815_00264	NBC2815_00264	-1.79	1.06E-02	xanthomonadin biosynthesis phosphotransferase/dehydratase
NBC2815_00265	NBC2815_00265	8.93	9.09E-03	IS1480 transposase
NBC2815_00268	NBC2815_00268	1.95	2.47E-05	dolichyl-phosphate mannose synthase-like protein
NBC2815_00269	NBC2815_00269	1.75	2.67E-04	Hypothetical Protein
fabF_1	NBC2815_00270	1.99	3.42E-05	3-oxoacyl-ACP synthase
NBC2815_00271	NBC2815_00271	1.56	5.65E-03	Hypothetical Protein
NBC2815_00273	NBC2815_00273	2.42	2.65E-03	phosphatase
NBC2815_00274	NBC2815_00274	4.81	2.89E-17	glycosyl hydrolase family protein
NBC2815_00275	NBC2815_00275	3.61	3.41E-12	glucose-6-phosphate 1-dehydrogenase
kefC_1	NBC2815_00276	8.36	4.82E-30	glutathione-regulated potassium-efflux system protein
kefC_2	NBC2815_00277	3.14	3.21E-02	glutathione-regulated potassium-efflux system protein
NBC2815_00279	NBC2815_00279	1.54	1.81E-02	Mg++ transporter
NBC2815_00280	NBC2815_00280	1.97	2.05E-05	acyl-CoA synthetase
NBC2815_00281	NBC2815_00281	-1.81	8.70E-05	integral membrane protein
nrdF	NBC2815_00283	-1.51	1.70E-02	ribonucleotide-diphosphate reductase subunit beta
NBC2815_00284	NBC2815_00284	-1.70	7.89E-04	flavodoxin
NBC2815_00290	NBC2815_00290	1.57	4.02E-03	avirulence protein
NBC2815_00297	NBC2815_00297	2.25	3.65E-02	acriflavin resistance protein
cnrB	NBC2815_00298	6.05	6.65E-06	Nickel and cobalt resistance protein CnrB
NBC2815_00299	NBC2815_00299	1.98	4.83E-03	phospholipid N-methyltransferase
NBC2815_00302	NBC2815_00302	-2.84	8.21E-09	hypothetical protein
NBC2815_00303	NBC2815_00303	-3.26	8.15E-09	hypothetical protein
NBC2815_00304	NBC2815_00304	-1.53	2.02E-02	hypothetical protein
NBC2815_00305	NBC2815_00305	-1.85	2.04E-04	Hypothetical Protein
NBC2815_00308	NBC2815_00308	-6.65	1.72E-23	putative reductase
NBC2815_00312	NBC2815_00312	-1.61	1.93E-03	ISXo1 transposase
NBC2815_00317	NBC2815_00317	3.24	1.16E-15	Hypothetical Protein
cbbFC	NBC2815_00319	-3.26	0.00E+00	fructose-1,6-bisphosphatase
NBC2815_00321	NBC2815_00321	1.58	3.09E-03	Hypothetical Protein
NBC2815_00322	NBC2815_00322	2.34	2.82E-06	LasA protein
NBC2815_00323	NBC2815_00323	2.70	1.39E-09	hypothetical protein
NBC2815_00324	NBC2815_00324	2.01	6.99E-03	AtsE protein
aes	NBC2815_00325	2.44	2.99E-03	acetyl esterase
NBC2815_00330	NBC2815_00330	9.72	1.61E-03	pyrroline-5-carboxylate reductase
NBC2815_00333	NBC2815_00333	4.26	1.66E-02	tis1421-transposase b
NBC2815_00334	NBC2815_00334	1.68	6.70E-04	Hypothetical Protein
NBC2815_00336	NBC2815_00336	3.71	8.70E-13	acetyl-CoA acetyltransferase /EC_number="2.3.1.9
NBC2815_00337	NBC2815_00337	6.39	1.34E-21	porphyrin biosynthesis protein
NBC2815_00338	NBC2815_00338	7.32	6.53E-27	heme biosynthesis enzyme
NBC2815_00342	NBC2815_00342	-1.64	7.80E-04	membrane-anchored phosphatase / sulfurtransferase
NBC2815_00346	NBC2815_00346	3.24	6.32E-10	pyruvate dehydrogenase
NBC2815_00348	NBC2815_00348	2.50	4.81E-06	two-component system sensor protein
NBC2815_00353	NBC2815_00353	-1.71	4.71E-03	Fic protein family protein
csy_1	NBC2815_00356	-1.83	1.11E-02	crispr-associated protein, Csy4 family
csy_2	NBC2815_00357	-1.85	1.50E-04	crispr-associated protein, Csy3 family

csy_4	NBC2815_00359	-1.59	1.24E-02	crispr-associated protein, Csy1 family
NBC2815_00360	NBC2815_00360	-1.87	4.15E-04	crispr-associated helicase Cas3
cas	NBC2815_00361	-3.28	8.24E-05	crispr-associated protein Cas1
NBC2815_00362	NBC2815_00362	-2.78	1.01E-04	hypothetical protein
NBC2815_00363	NBC2815_00363	-3.34	6.77E-14	biotin carboxylase
NBC2815_00364	NBC2815_00364	-3.39	2.83E-14	acyl-CoA carboxyltransferase subunit beta
NBC2815_00365	NBC2815_00365	-2.48	1.56E-06	isovaleryl-CoA dehydrogenase
NBC2815_00366	NBC2815_00366	-1.58	7.98E-03	AcrR family transcriptional regulator
NBC2815_00369	NBC2815_00369	-2.19	3.00E-06	cytochrome C5
NBC2815_00371	NBC2815_00371	2.46	3.08E-05	Hypothetical Protein
NBC2815_00372	NBC2815_00372	4.13	3.76E-12	nitrile hydratase activator
NBC2815_00373	NBC2815_00373	4.91	3.32E-13	nitrile hydratase activator
xopR	NBC2815_00374	2.40	3.74E-05	type III effector protein XopR
NBC2815_00375	NBC2815_00375	-2.20	2.10E-08	transmembrane protein
ohr	NBC2815_00377	-3.34	1.47E-12	organic hydroperoxide resistance protein
NBC2815_00378	NBC2815_00378	4.73	2.90E-24	Hypothetical Protein
NBC2815_00380	NBC2815_00380	1.54	8.46E-03	quinone oxidoreductase
NBC2815_00382	NBC2815_00382	-3.11	7.00E-12	methyltransferase
NBC2815_00383	NBC2815_00383	-2.71	1.08E-04	pseudouridylate synthase
NBC2815_00386	NBC2815_00386	2.52	4.72E-07	Hypothetical Protein
hrpB	NBC2815_00387	3.66	6.67E-14	ATP-dependent helicase HrpB
NBC2815_00391	NBC2815_00391	-1.77	5.43E-04	type V secretory pathway protein
NBC2815_00394	NBC2815_00394	2.95	1.60E-08	NADH flavin oxidoreductase
purU	NBC2815_00395	-1.67	4.46E-04	formyltetrahydrofolate deformylase
NBC2815_00397	NBC2815_00397	4.02	1.33E-04	multidrug efflux transporter
NBC2815_00401	NBC2815_00401	1.67	3.99E-02	ISXac3 transposase, IS3 family
NBC2815_00405	NBC2815_00405	3.40	9.55E-03	putative signal protein with HD-GYP domain protein
NBC2815_00406	NBC2815_00406	1.63	1.48E-02	putative signal protein with HD-GYP domain protein
NBC2815_00411	NBC2815_00411	3.57	2.30E-05	oxidoreductase
metE	NBC2815_00413	2.11	8.52E-05	5-methyltetrahydropteroyltriglutamate-- homocysteine methyltransferase
NBC2815_00420	NBC2815_00420	2.46	2.02E-04	ISXac3 transposase, IS3 family
glpK	NBC2815_00423	-2.41	4.88E-08	glycerol kinase
NBC2815_00425	NBC2815_00425	1.66	1.13E-02	glycerol-3-phosphate dehydrogenase
NBC2815_00428	NBC2815_00428	1.61	2.05E-03	serine/threonine protein kinase
NBC2815_00431	NBC2815_00431	2.36	1.04E-06	lipoprotein
NBC2815_00435	NBC2815_00435	1.66	2.34E-03	aspartyl-asparaginyl beta-hydroxylase
NBC2815_00438	NBC2815_00438	2.71	2.10E-06	oxidoreductase
bioF	NBC2815_00441	-2.03	3.24E-07	8-amino-7-oxononanoate synthase
bioB	NBC2815_00442	-3.17	1.09E-13	Biotin synthase
NBC2815_00448	NBC2815_00448	2.89	5.51E-10	putative protein kinase
NBC2815_00449	NBC2815_00449	2.62	8.00E-09	inner membrane protein YedI
NBC2815_00450	NBC2815_00450	2.35	4.14E-08	YngK protein
NBC2815_00451	NBC2815_00451	3.48	5.57E-08	signal transduction protein
glgA	NBC2815_00452	1.92	2.26E-04	glycogen synthase
NBC2815_00454	NBC2815_00454	-1.78	9.56E-05	malto-oligosyltrehalose trehalohydrolase
glgY	NBC2815_00456	-1.51	1.17E-02	putative maltooligosyl trehalose synthase
NBC2815_00461	NBC2815_00461	-1.61	1.64E-03	ISXac3 transposase, IS3 family
RhsA	NBC2815_00464	-3.32	3.12E-07	truncated rhs family protein
NBC2815_00465	NBC2815_00465	-1.96	7.77E-03	IS1478 transposase
dsbD	NBC2815_00469	3.42	6.81E-12	C-type cytochrome biogenesis protein (copper tolerance)
cutA	NBC2815_00470	2.90	1.44E-12	divalent cation tolerance protein
NBC2815_00471	NBC2815_00471	2.69	8.34E-03	hypothetical protein
NBC2815_00472	NBC2815_00472	2.86	6.46E-11	ribonuclease
NBC2815_00477	NBC2815_00477	4.08	8.81E-12	TonB-dependent outer membrane receptor
aroG	NBC2815_00478	2.80	7.52E-12	phospho-2-dehydro-3-deoxyheptonate aldolase
NBC2815_00480	NBC2815_00480	2.89	2.03E-13	Hypothetical Protein
NBC2815_00484	NBC2815_00484	-2.66	1.60E-08	transmembrane protein
NBC2815_00485	NBC2815_00485	2.18	1.97E-04	nucleoside-diphosphate-sugar epimerase
NBC2815_00493	NBC2815_00493	-1.85	1.19E-04	hypothetical protein
NBC2815_00494	NBC2815_00494	-1.70	4.58E-04	hypothetical protein
NBC2815_00499	NBC2815_00499	-1.89	7.30E-06	malonate decarboxylase subunit alpha
NBC2815_00500	NBC2815_00500	-2.14	5.20E-03	malonate decarboxylase subunit delta
NBC2815_00501	NBC2815_00501	-4.53	1.25E-04	hypothetical protein
NBC2815_00502	NBC2815_00502	-2.09	1.76E-05	malonate decarboxylase subunit beta
NBC2815_00503	NBC2815_00503	-1.71	3.26E-03	malonate decarboxylase subunit gamma
NBC2815_00506	NBC2815_00506	-2.14	7.78E-03	hypothetical protein
NBC2815_00507	NBC2815_00507	2.20	2.68E-05	ACP S-malonyltransferase
NBC2815_00508	NBC2815_00508	2.57	6.26E-07	dicarboxylate carrier protein
NBC2815_00510	NBC2815_00510	1.57	3.01E-03	anti-sigma F factor antagonist
NBC2815_00513	NBC2815_00513	-1.78	2.21E-03	putative secreted protein
NBC2815_00514	NBC2815_00514	-1.63	4.32E-03	lipase
NBC2815_00515	NBC2815_00515	2.48	8.13E-04	lipase
aceE	NBC2815_00516	-2.02	2.81E-05	pyruvate dehydrogenase
NBC2815_00517	NBC2815_00517	-2.73	2.32E-02	hypothetical protein
NBC2815_00518	NBC2815_00518	-3.13	0.00E+00	hypothetical protein
NBC2815_00519	NBC2815_00519	-2.96	8.66E-12	hypothetical protein
NBC2815_00523	NBC2815_00523	-5.59	2.49E-31	putative peptidoglycan binding domain-containing protein
NBC2815_00524	NBC2815_00524	-5.55	2.56E-29	hypothetical protein
NBC2815_00525	NBC2815_00525	2.81	8.89E-04	Transposase IS200 like protein
clcB_1	NBC2815_00526	3.36	3.72E-13	chloride channel
clcB_2	NBC2815_00527	4.91	5.28E-11	chloride channel
NBC2815_00529	NBC2815_00529	3.37	1.96E-05	sulfate permease
NBC2815_00531	NBC2815_00531	2.31	3.85E-07	Hypothetical Protein
NBC2815_00532	NBC2815_00532	2.34	2.98E-02	carboxypeptidase
NBC2815_00541	NBC2815_00541	3.74	1.82E-18	hypothetical protein
NBC2815_00542	NBC2815_00542	2.33	1.17E-04	Hypothetical Protein
NBC2815_00543	NBC2815_00543	2.34	2.98E-08	Hypothetical Protein
NBC2815_00544	NBC2815_00544	-2.16	1.75E-05	hypothetical protein
msbA	NBC2815_00550	2.46	1.04E-08	ATP-binding transporter 1
NBC2815_00551	NBC2815_00551	1.80	3.07E-04	Hypothetical Protein
NBC2815_00552	NBC2815_00552	1.63	1.35E-02	UDP-galactopyranose mutase
NBC2815_00553	NBC2815_00553	4.05	8.64E-14	glycosyltransferase
galE	NBC2815_00554	5.00	7.50E-20	UDP-glucose 4-epimerase
NBC2815_00555	NBC2815_00555	3.48	1.75E-10	oxidoreductase

NBC2815_00558	NBC2815_00558	1.52	2.03E-02	sigma-54 dependent response regulator
NBC2815_00562	NBC2815_00562	-1.69	2.77E-03	Hypothetical Protein
NBC2815_00565	NBC2815_00565	1.60	3.55E-02	protein YciE
ybeC	NBC2815_00566	-2.83	1.45E-12	amino acid transporter
NBC2815_00567	NBC2815_00567	-1.50	2.10E-02	Hypothetical Protein
NBC2815_00568	NBC2815_00568	1.68	2.34E-03	Hypothetical Protein
NBC2815_00572	NBC2815_00572	2.00	2.99E-06	DNA-binding protein with the Helix-hairpin-helix motif protein
NBC2815_00574	NBC2815_00574	-2.29	1.01E-06	beta-Ig-H3/fasciclin repeat containing protein
NBC2815_00575	NBC2815_00575	1.83	6.14E-05	tis1421-transposase b
NBC2815_00576	NBC2815_00576	3.37	6.33E-04	transposase
NBC2815_00577	NBC2815_00577	1.90	9.09E-03	hypothetical protein
NBC2815_00578	NBC2815_00578	1.72	1.62E-04	Na+:H+ antiporter
NBC2815_00579	NBC2815_00579	2.94	8.64E-07	Hypothetical Protein
NBC2815_00582	NBC2815_00582	1.83	2.85E-04	hypothetical protein
NBC2815_00587	NBC2815_00587	2.11	2.77E-05	hypothetical protein
NBC2815_00588	NBC2815_00588	1.74	2.18E-03	methanol dehydrogenase regulatory protein
NBC2815_00589	NBC2815_00589	1.86	6.93E-04	Hypothetical Protein
NBC2815_00590	NBC2815_00590	2.44	1.09E-08	Hypothetical Protein
NBC2815_00591	NBC2815_00591	2.34	2.77E-07	von Willebrand factor A
NBC2815_00593	NBC2815_00593	1.66	4.36E-03	Hypothetical Protein
gltP	NBC2815_00594	-2.15	2.11E-07	proton glutamate symport protein
NBC2815_00595	NBC2815_00595	-1.89	1.29E-04	transketolase /EC_number="2.2.1.1
fhuE	NBC2815_00596	3.40	7.88E-17	ferric iron uptake outer membrane protein
NBC2815_00597	NBC2815_00597	1.86	1.06E-03	metalloendopeptidase
NBC2815_00598	NBC2815_00598	1.94	2.37E-04	putative secreted protein
NBC2815_00601	NBC2815_00601	1.79	5.42E-04	putative TonB-like protein
NBC2815_00606	NBC2815_00606	-1.55	1.27E-02	beta-lactamase
NBC2815_00607	NBC2815_00607	-1.98	1.18E-05	endonuclease
NBC2815_00608	NBC2815_00608	-2.34	4.04E-05	outer membrane protein
gapA	NBC2815_00609	-4.84	0.00E+00	glyceraldehyde-3-phosphate dehydrogenase
NBC2815_00610	NBC2815_00610	-3.11	1.16E-13	Hypothetical Protein
NBC2815_00616	NBC2815_00616	-2.19	1.00E-06	Histidine kinase/response regulator hybrid protein
NBC2815_00617	NBC2815_00617	-2.33	2.08E-07	hypothetical protein
NBC2815_00619	NBC2815_00619	2.34	1.14E-03	lipoprotein
pgk	NBC2815_00622	-2.06	6.72E-07	phosphoglycerate kinase
NBC2815_00623	NBC2815_00623	-1.69	7.11E-04	indigoidine synthesis-like protein
NBC2815_00625	NBC2815_00625	-1.61	8.81E-03	fructose-bisphosphate aldolase
NBC2815_00626	NBC2815_00626	4.65	1.02E-14	RNase III inhibitor
NBC2815_00627	NBC2815_00627	4.44	1.39E-21	cysteine synthase
cysG	NBC2815_00628	11.50	1.17E-54	siroheme synthase
NBC2815_00629	NBC2815_00629	1.53	2.77E-02	transcriptional regulator CysB-like protein
NBC2815_00634	NBC2815_00634	2.13	7.77E-04	iron-regulated membrane protein
NBC2815_00635	NBC2815_00635	16.09	2.00E-48	phosphoadenosine phosphosulfate reductase /EC_number="1.8.4.8
cysI	NBC2815_00636	50.84	3.40E-71	sulfite reductase (NADPH) hemoprotein subunit beta
NBC2815_00637	NBC2815_00637	105.92	6.89E-65	NADPH-sulfite reductase flavoprotein subunit
cysD	NBC2815_00638	23.27	7.06E-63	sulfate adenylyltransferase subunit 2
nodQ	NBC2815_00639	9.61	2.42E-33	bifunctional sulfate adenylyltransferase subunit 1/adenylylsulfate kinase
NBC2815_00640	NBC2815_00640	-2.96	2.54E-13	RND efflux membrane fusion protein
NBC2815_00641	NBC2815_00641	-2.28	5.26E-08	acriflavin resistance protein
NBC2815_00650	NBC2815_00650	-1.67	6.62E-04	3-deoxy-7-phosphoheptulonate synthase /EC_number="2.5.1.54
NBC2815_00652	NBC2815_00652	1.63	1.33E-02	Hypothetical Protein
NBC2815_00654	NBC2815_00654	-1.67	1.49E-03	peptidyl-prolyl cis-trans isomerase
xopAD_1	NBC2815_00657	1.58	9.44E-03	Xanthomonas outer protein AD, type III effector AD
NBC2815_00658	NBC2815_00658	8.54	3.19E-10	glutathione S-transferase
NBC2815_00659	NBC2815_00659	9.98	1.85E-21	cell wall hydrolase superfamily protein
NBC2815_00660	NBC2815_00660	-1.57	4.35E-03	ISXo1 transposase, IS5 family
NBC2815_00662	NBC2815_00662	2.24	6.42E-07	2,4-dienoyl-CoA reductase
NBC2815_00663	NBC2815_00663	4.37	1.88E-18	oxidoreductase
NBC2815_00665	NBC2815_00665	-3.05	3.42E-15	Hypothetical Protein
NBC2815_00666	NBC2815_00666	-1.92	6.83E-05	pseudouridylate synthase
NBC2815_00669	NBC2815_00669	1.69	2.51E-03	ABC transporter sulfate binding protein
NBC2815_00670	NBC2815_00670	2.41	1.58E-07	sulfate ABC transporter permease
NBC2815_00671	NBC2815_00671	1.86	2.25E-04	sulfate ABC transporter permease
cysA	NBC2815_00672	1.50	1.56E-02	sulfate ABC transporter ATP-binding protein
tdh	NBC2815_00674	-2.06	1.41E-06	L-threonine 3-dehydrogenase
pgmA	NBC2815_00677	-2.08	3.98E-07	phosphoglycerate mutase
xrvA	NBC2815_00678	-2.44	1.13E-07	virulence regulator
glgX2	NBC2815_00679	1.90	3.68E-04	isoamylase
NBC2815_00681	NBC2815_00681	-1.64	5.09E-03	Hypothetical Protein
NBC2815_00686	NBC2815_00686	2.40	3.11E-03	Hypothetical Protein
NBC2815_00692	NBC2815_00692	2.63	3.95E-06	Hypothetical Protein
NBC2815_00695	NBC2815_00695	-1.81	2.07E-05	glycosyltransferase
ompR	NBC2815_00696	-1.58	6.05E-03	DNA-binding response regulator in two-component regulatory system with EnvZ
NBC2815_00697	NBC2815_00697	-1.76	4.07E-02	two-component system sensor protein
NBC2815_00701	NBC2815_00701	-2.42	1.69E-04	disulfide oxidoreductase
dsbA	NBC2815_00702	-4.27	0.00E+00	thiol:disulfide interchange protein
NBC2815_00703	NBC2815_00703	-1.82	2.79E-05	endonuclease-exonuclease-phosphatase
NBC2815_00704	NBC2815_00704	-1.89	1.03E-05	alcohol dehydrogenase
xopAD_2	NBC2815_00705	3.11	1.15E-11	type III effector protein XopAD
NBC2815_00707	NBC2815_00707	1.80	4.12E-04	multifunctional CCA protein
NBC2815_00715	NBC2815_00715	-1.69	4.02E-02	Hypothetical Protein
NBC2815_00716	NBC2815_00716	-3.84	2.26E-09	Hypothetical Protein
NBC2815_00718	NBC2815_00718	-2.84	1.68E-12	Hypothetical protein
motB	NBC2815_00719	7.23	8.06E-12	flagellar motor protein MotB
motA2	NBC2815_00720	15.50	6.21E-18	flagellar motor protein
NBC2815_00721	NBC2815_00721	4.82	2.06E-09	methionine sulfoxide reductase B /EC_number="1.8.4.12
NBC2815_00722	NBC2815_00722	5.25	1.30E-20	Hypothetical Protein
NBC2815_00723	NBC2815_00723	5.43	2.78E-21	leucine responsive regulatory protein
NBC2815_00724	NBC2815_00724	1.59	4.36E-03	D-amino acid dehydrogenase small subunit /EC_number="1.4.99.6
NBC2815_00727	NBC2815_00727	6.54	1.70E-24	Hypothetical Protein
NBC2815_00728	NBC2815_00728	7.13	1.14E-21	Hypothetical Protein
NBC2815_00729	NBC2815_00729	3.71	1.41E-14	Hypothetical Protein
NBC2815_00730	NBC2815_00730	4.36	1.79E-18	Hypothetical Protein
NBC2815_00731	NBC2815_00731	3.04	3.28E-10	putative sensory/regulatory hybrid protein of a two component system

NBC2815_00732	NBC2815_00732	2.41	8.63E-08	Hypothetical Protein
NBC2815_00733	NBC2815_00733	1.70	7.91E-03	L-sorbosone dehydrogenase
NBC2815_00736	NBC2815_00736	-1.76	4.58E-03	ribosomal small subunit pseudouridylate synthase
NBC2815_00738	NBC2815_00738	2.42	7.40E-08	transposase
NBC2815_00739	NBC2815_00739	1.84	5.88E-03	tis1421-transposase b
NBC2815_00741	NBC2815_00741	1.82	1.10E-03	Hypothetical Protein
NBC2815_00742	NBC2815_00742	8.22	1.07E-06	two-component system sensor histidine kinase-response regulator hybrid protein
NBC2815_00746	NBC2815_00746	3.87	2.94E-04	hypothetical protein
NBC2815_00751	NBC2815_00751	2.02	5.01E-04	OmpW family protein
aceF	NBC2815_00753	-1.58	6.52E-03	dihydrolipoamide acetyltransferase
NBC2815_00754	NBC2815_00754	-2.30	9.07E-06	Hypothetical Protein
NBC2815_00756	NBC2815_00756	3.26	1.79E-11	Hypothetical Protein
NBC2815_00757	NBC2815_00757	2.47	4.93E-07	Hypothetical Protein
NBC2815_00759	NBC2815_00759	-1.82	2.00E-04	F0F1 ATP synthase subunit A /EC_number="3.6.3.14
NBC2815_00760	NBC2815_00760	-1.81	3.22E-04	F0F1 ATP synthase subunit C /EC_number="3.6.3.14
NBC2815_00761	NBC2815_00761	-3.17	2.79E-11	F0F1 ATP synthase subunit B /EC_number="3.6.3.14
NBC2815_00762	NBC2815_00762	-1.69	1.50E-03	F0F1 ATP synthase subunit delta /EC_number="3.6.3.14
ATPA	NBC2815_00763	-2.17	3.08E-06	ATP synthase subunit alpha
NBC2815_00764	NBC2815_00764	-3.57	1.75E-13	F0F1 ATP synthase subunit gamma /EC_number="3.6.3.14
ATPD	NBC2815_00765	-2.77	2.62E-09	ATP synthase subunit beta
atpC	NBC2815_00766	-2.47	4.04E-09	F0F1 ATP synthase subunit epsilon
glmU	NBC2815_00769	-1.55	2.83E-03	UDP-N-acetylglucosamine pyrophosphorylase
NBC2815_00770	NBC2815_00770	1.58	1.10E-02	hypothetical protein
NBC2815_00771	NBC2815_00771	1.93	5.56E-05	two-component system sensor histidine kinase
NBC2815_00772	NBC2815_00772	2.49	1.53E-06	two-component system sensor histidine kinase
NBC2815_00774	NBC2815_00774	6.17	7.81E-17	Hypothetical Protein
NBC2815_00775	NBC2815_00775	2.41	1.61E-06	Hypothetical Protein
copB	NBC2815_00777	-2.48	1.31E-06	copper resistance protein B
copA	NBC2815_00778	-1.96	4.22E-06	copper resistance protein A
cysM	NBC2815_00779	-2.07	1.50E-06	cysteine synthase
NBC2815_00781	NBC2815_00781	-1.76	3.84E-02	hypothetical protein
NBC2815_00782	NBC2815_00782	-4.44	1.30E-23	beta-ketoacyl-[ACP] synthase I
fabA	NBC2815_00783	-4.05	5.25E-21	3-hydroxydecanoyl-ACP dehydratase
dinP	NBC2815_00784	-1.75	1.35E-04	DNA polymerase IV
NBC2815_00786	NBC2815_00786	-1.79	4.14E-02	ISXo1 transposase, IS5 family
NBC2815_00793	NBC2815_00793	21.19	5.65E-35	Hypothetical Protein
NBC2815_00794	NBC2815_00794	3.92	2.46E-17	6-pyruvoyl tetrahydrobiopterin synthase
rhlE	NBC2815_00795	6.94	4.23E-24	ATP-dependent RNA helicase
mhpD	NBC2815_00796	-3.58	3.31E-22	2-keto-4-pentenoate hydratase
NBC2815_00797	NBC2815_00797	-2.46	1.23E-08	maleylacetoacetate isomerase
NBC2815_00799	NBC2815_00799	-1.60	1.31E-03	outer membrane protein
NBC2815_00801	NBC2815_00801	2.24	3.07E-04	UptF protein
cysB	NBC2815_00802	-1.51	1.29E-02	cystathionine beta-synthase
NBC2815_00803	NBC2815_00803	-2.02	7.46E-06	cystathionine gamma-synthase
NBC2815_00804	NBC2815_00804	-2.82	5.22E-13	glycosyltransferase
wzm	NBC2815_00805	-3.15	1.27E-09	permease of an ABC-type polysaccharide exporter
NBC2815_00806	NBC2815_00806	-4.76	1.22E-20	ABC transporter ATP binding protein
wxcB	NBC2815_00807	-3.03	3.41E-12	protein kinase
NBC2815_00808	NBC2815_00808	-4.69	9.45E-29	glycosyltransferase
NBC2815_00809	NBC2815_00809	-4.55	0.00E+00	membrane protein WxcD
NBC2815_00810	NBC2815_00810	-5.47	0.00E+00	membrane protein WxcE
gmd	NBC2815_00811	-6.03	0.00E+00	GDP-mannose 4,6-dehydratase
NBC2815_00812	NBC2815_00812	-5.22	0.00E+00	UDP-glucose 4-epimerase
NBC2815_00813	NBC2815_00813	-5.13	8.14E-32	integral membrane protein WxcO
NBC2815_00814	NBC2815_00814	-3.63	3.12E-15	hypothetical protein
NBC2815_00815	NBC2815_00815	-1.64	8.27E-03	GtrA-like protein
NBC2815_00816	NBC2815_00816	-1.98	1.25E-06	putative glycosyltransferase
NBC2815_00817	NBC2815_00817	-2.12	8.66E-07	hypothetical protein
NBC2815_00818	NBC2815_00818	-2.49	1.71E-09	electron transfer flavoprotein alpha subunit
etfB	NBC2815_00819	-2.74	4.59E-11	electron transfer flavoprotein beta subunit
rmlB	NBC2815_00820	-2.51	3.06E-11	dTDP-glucose 4,6-dehydratase
rmlA	NBC2815_00821	-2.59	6.17E-07	glucose-1-phosphate thymidylyltransferase
NBC2815_00822	NBC2815_00822	-2.47	9.88E-07	dTDP-4-dehydrorhamnose 3,5-epimerase
ipsI	NBC2815_00826	-1.63	1.92E-03	IpsJ protein
NBC2815_00827	NBC2815_00827	-1.73	9.71E-04	IpsJ protein
NBC2815_00828	NBC2815_00828	-2.08	4.72E-06	Lipopolysaccharide biosynthesis protein
NBC2815_00830	NBC2815_00830	1.57	2.62E-02	DNA repair system specific for alkylated DNA
NBC2815_00831	NBC2815_00831	1.54	5.40E-03	lipoprotein
NBC2815_00833	NBC2815_00833	1.93	5.69E-06	ABC transporter ATP-binding protein
NBC2815_00834	NBC2815_00834	2.00	1.04E-04	ABC transporter permease
NBC2815_00836	NBC2815_00836	-2.05	3.99E-05	DNA-binding protein
proS	NBC2815_00837	-2.87	2.11E-12	putative prolyl-tRNA synthetase
NBC2815_00838	NBC2815_00838	-2.54	1.69E-09	Putative secreted protein
NBC2815_00841	NBC2815_00841	-1.59	3.03E-02	ribosomal-protein-alanine acetyltransferase
NBC2815_00851	NBC2815_00851	-1.91	3.77E-05	DNA-damage-inducible protein d
NBC2815_00852	NBC2815_00852	-1.96	7.89E-04	type I restriction enzyme M protein
xopC_1	NBC2815_00855	2.81	9.14E-07	outer protein C
xopAF	NBC2815_00856	-1.74	8.32E-05	type III effector protein XopAF
NBC2815_00858	NBC2815_00858	2.17	2.54E-06	Hypothetical Protein
bga2	NBC2815_00859	-1.71	1.21E-04	beta-galactosidase
NBC2815_00869	NBC2815_00869	2.10	1.86E-07	Hypothetical Protein
NBC2815_00870	NBC2815_00870	6.19	7.74E-23	peptidyl-Asp metalloendopeptidase
lpxH	NBC2815_00872	-1.57	6.82E-03	UDP-2,3-diacylglucosamine hydrolase
NBC2815_00874	NBC2815_00874	-1.68	4.83E-02	glycosyl transferase
NBC2815_00879	NBC2815_00879	2.00	2.59E-05	Zn-dependent protease
grxC	NBC2815_00880	1.66	9.59E-03	glutaredoxin
NBC2815_00881	NBC2815_00881	1.54	2.36E-02	carboxymuconolactone decarboxylase
NBC2815_00884	NBC2815_00884	-1.51	1.44E-02	membrane protein
NBC2815_00889	NBC2815_00889	1.51	6.76E-03	hypothetical protein
NBC2815_00890	NBC2815_00890	4.74	2.86E-18	IS1478 transposase
NBC2815_00897	NBC2815_00897	2.72	1.80E-08	VGR-like protein
NBC2815_00900	NBC2815_00900	-2.28	1.98E-05	ISXo2 transposase
NBC2815_00901	NBC2815_00901	-1.91	3.65E-04	ISXo2 transposase
NBC2815_00902	NBC2815_00902	2.03	7.92E-06	VGR-like protein

NBC2815_00903	NBC2815_00903	2.25	1.02E-03	hypothetical protein
NBC2815_00905	NBC2815_00905	-2.48	1.17E-04	ISXo2 transposase
NBC2815_00914	NBC2815_00914	1.56	2.35E-02	siderophore biosynthesis protein
NBC2815_00915	NBC2815_00915	2.29	1.80E-06	Biotin carboxylase
NBC2815_00916	NBC2815_00916	2.47	9.90E-07	citrate-dependent iron transporter
mphE	NBC2815_00917	3.31	7.97E-10	4-hydroxy-2-oxovalerate aldolase
NBC2815_00919	NBC2815_00919	-1.91	2.81E-05	IS1478 transposase
NBC2815_00920	NBC2815_00920	-3.00	1.96E-04	hypothetical protein
NBC2815_00923	NBC2815_00923	-1.77	5.88E-03	AraC/XylS family transcriptional regulator
gcvP	NBC2815_00926	-2.37	4.28E-07	glycine dehydrogenase
NBC2815_00930	NBC2815_00930	-2.05	8.43E-06	dipeptidyl carboxypeptidase
NBC2815_00934	NBC2815_00934	2.91	4.12E-02	dolichyl-phosphate-mannose-protein mannosyltransferase
NBC2815_00938	NBC2815_00938	-3.42	6.74E-06	Hypothetical Protein
minE	NBC2815_00939	-2.84	2.67E-12	cell division topological specificity factor MinE
minD	NBC2815_00940	-3.09	5.97E-13	septum site-determining protein
NBC2815_00943	NBC2815_00943	-1.50	4.08E-02	two-component system sensor protein
NBC2815_00944	NBC2815_00944	-3.12	4.88E-11	two-component system regulatory protein
NBC2815_00945	NBC2815_00945	-5.87	5.79E-07	hypothetical protein
NBC2815_00946	NBC2815_00946	-2.90	1.42E-10	Hypothetical Protein
NBC2815_00947	NBC2815_00947	-2.83	9.98E-05	Hypothetical Protein
NBC2815_00948	NBC2815_00948	-1.75	7.06E-03	DNA-3-methyladenine glycosylase
NBC2815_00949	NBC2815_00949	1.93	3.64E-04	D-alanyl-D-alanine carboxypeptidase
NBC2815_00952	NBC2815_00952	1.71	2.33E-02	Hypothetical Protein
NBC2815_00953	NBC2815_00953	2.55	2.28E-07	Hypothetical Protein
purT	NBC2815_00955	-1.55	3.54E-03	phosphoribosylglycinamide formyltransferase 2
NBC2815_00958	NBC2815_00958	-1.69	3.68E-02	hypothetical protein
NBC2815_00959	NBC2815_00959	1.90	1.33E-02	IS1478 transposase
NBC2815_00964	NBC2815_00964	2.96	1.01E-05	EF hand domain-containing protein
NBC2815_00965	NBC2815_00965	5.43	4.13E-09	hypothetical protein
NBC2815_00966	NBC2815_00966	-1.87	7.62E-04	pathogenicity-like protein
NBC2815_00969	NBC2815_00969	-3.44	4.96E-10	Hypothetical Protein
ileS	NBC2815_00978	-1.88	9.41E-05	isoleucyl-tRNA ligase
ispH	NBC2815_00980	-1.60	2.59E-03	4-hydroxy-3-methylbut-2-enyl diphosphate reductase
NBC2815_00982	NBC2815_00982	-2.50	3.24E-04	Cytochrome O ubiquinol oxidase subunit II /EC_number="1.10.3.-
cyoB	NBC2815_00983	-2.52	4.95E-04	cytochrome O ubiquinol oxidase subunit I
cyoC	NBC2815_00984	-1.71	3.85E-02	putative cytochrome o ubiquinol oxidase subunit III
NBC2815_00985	NBC2815_00985	-1.72	3.31E-02	cytochrome O ubiquinol oxidase subunit IV
NBC2815_00986	NBC2815_00986	-8.34	6.18E-26	hypothetical protein
NBC2815_00987	NBC2815_00987	-2.13	3.32E-05	hypothetical protein
NBC2815_00988	NBC2815_00988	3.98	2.02E-06	Hypothetical Protein
hrpG	NBC2815_00989	8.55	3.09E-20	HrpG protein
hrpX	NBC2815_00990	4.40	7.86E-15	HrpX
NBC2815_00994	NBC2815_00994	4.24	1.26E-02	TonB-dependent receptor
NBC2815_00995	NBC2815_00995	3.61	8.11E-03	two-component system sensor protein
NBC2815_00997	NBC2815_00997	-1.69	1.68E-03	two-component system sensor protein
NBC2815_01002	NBC2815_01002	-2.88	1.22E-08	ISXo1 transposase, IS5 family
hemE	NBC2815_01005	-1.58	1.13E-03	uroporphyrinogen decarboxylase
NBC2815_01006	NBC2815_01006	-2.19	2.37E-04	Hypothetical Protein
aroK	NBC2815_01008	1.50	4.39E-02	shikimate kinase I
NBC2815_01009	NBC2815_01009	-1.56	7.98E-03	pyridoxamine 5'-phosphate oxidase /EC_number="1.4.3.5
NBC2815_01010	NBC2815_01010	-2.27	3.46E-09	Hypothetical Protein
NBC2815_01011	NBC2815_01011	-1.71	1.10E-03	PspA/IM30 family protein
NBC2815_01012	NBC2815_01012	-1.65	2.02E-03	Hypothetical Protein
NBC2815_01013	NBC2815_01013	-1.82	4.44E-04	membrane protein
NBC2815_01014	NBC2815_01014	-1.94	1.95E-05	lipoprotein
NBC2815_01015	NBC2815_01015	-2.80	1.67E-12	synthetase/amidase
NBC2815_01017	NBC2815_01017	2.14	1.65E-02	peptidase
NBC2815_01018	NBC2815_01018	3.43	2.50E-06	IS1480 transposase
NBC2815_01019	NBC2815_01019	3.39	1.48E-04	IS1480 transposase
NBC2815_01021	NBC2815_01021	2.08	2.67E-03	Amino acid-polyamine-organocation superfamily protein
ahyR	NBC2815_01022	2.32	2.02E-07	AhyR/AsaR family transcriptional regulator
NBC2815_01023	NBC2815_01023	4.42	1.10E-18	proline imino-peptidase
NBC2815_01024	NBC2815_01024	3.88	2.46E-06	methyl-accepting chemotaxis protein
NBC2815_01027	NBC2815_01027	-2.30	1.42E-08	Amino acid transporter family protein
NBC2815_01028	NBC2815_01028	-2.45	3.19E-08	peptidase
NBC2815_01030	NBC2815_01030	1.72	4.65E-03	relaxation protein
kefB2	NBC2815_01031	1.85	2.93E-05	glutathione-regulated potassium-efflux protein B
NBC2815_01034	NBC2815_01034	5.53	3.44E-16	transposase, fragment
NBC2815_01035	NBC2815_01035	2.04	3.74E-03	IS4 family transposase
NBC2815_01036	NBC2815_01036	4.49	1.32E-14	ferric enterobactin receptor
NBC2815_01039	NBC2815_01039	3.15	3.20E-12	lipoprotein
aspS	NBC2815_01040	-2.26	2.66E-07	aspartyl-tRNA synthetase
NBC2815_01043	NBC2815_01043	-3.45	4.91E-17	Hypothetical Protein
ruvC	NBC2815_01044	-2.43	3.60E-07	Holliday junction resolvase
ruvA	NBC2815_01045	-2.07	3.98E-07	Holliday junction DNA helicase RuvA
NBC2815_01046	NBC2815_01046	2.08	2.24E-05	potassium uptake protein
ompP6	NBC2815_01053	-3.05	6.29E-11	outer membrane protein P6
NBC2815_01054	NBC2815_01054	-2.14	3.58E-06	tol-pal system protein YbgF
NBC2815_01056	NBC2815_01056	2.28	1.69E-02	hypothetical protein
NBC2815_01063	NBC2815_01063	-1.78	9.13E-04	histone-like protein
lon	NBC2815_01064	2.57	2.30E-05	ATP-dependent serine proteinase La
clpP	NBC2815_01066	2.35	4.01E-05	ATP-dependent clp protease proteolytic subunit protein
NBC2815_01071	NBC2815_01071	2.13	2.35E-02	excinuclease ABC subunit A
NBC2815_01072	NBC2815_01072	2.23	6.97E-05	transducer protein car
NBC2815_01075	NBC2815_01075	3.20	7.65E-03	IS1404 transposase protein B
NBC2815_01077	NBC2815_01077	3.73	2.80E-11	dehydrogenase
NBC2815_01078	NBC2815_01078	2.13	4.01E-05	hydroxylase large subunit
NBC2815_01079	NBC2815_01079	2.04	3.22E-06	hydroxylase molybdopterin-containing subunit
NBC2815_01080	NBC2815_01080	2.46	4.40E-09	ferredoxin
NBC2815_01081	NBC2815_01081	-1.54	2.22E-02	Hypothetical Protein
NBC2815_01087	NBC2815_01087	1.56	2.17E-02	Hypothetical Protein
NBC2815_01089	NBC2815_01089	2.22	1.11E-04	Hypothetical Protein
NBC2815_01098	NBC2815_01098	2.09	9.08E-07	metal dependent phosphohydrolase superfamily protein
NBC2815_01101	NBC2815_01101	-3.74	0.00E+00	dipeptidyl peptidase

NBC2815_01102	NBC2815_01102	-2.35	4.25E-05	hypothetical protein
mutM	NBC2815_01103	2.03	2.72E-04	formamidopyrimidine-DNA glycosylase
NBC2815_01104	NBC2815_01104	2.52	8.02E-07	IS1478 transposase
dniR_1	NBC2815_01105	5.35	1.24E-20	murein hydrolase D
NBC2815_01106	NBC2815_01106	2.74	4.04E-09	hydroxyacylglutathione hydrolase /EC_number="3.1.2.6
rnhA	NBC2815_01108	-1.85	1.54E-04	ribonuclease H
NBC2815_01109	NBC2815_01109	-1.67	3.50E-04	DNA polymerase III subunit epsilon
NBC2815_01112	NBC2815_01112	-2.58	3.63E-07	Hypothetical Protein
moaA	NBC2815_01116	1.78	6.22E-05	molybdenum cofactor biosynthesis protein A
NBC2815_01117	NBC2815_01117	2.17	3.18E-02	hypothetical protein
moaC	NBC2815_01118	1.67	2.92E-03	molybdenum cofactor biosynthesis protein MoaC
moaE	NBC2815_01120	1.62	4.78E-03	molybdopterin converting factor subunit 2
NBC2815_01123	NBC2815_01123	-2.17	1.77E-07	Hypothetical Protein
rpmF	NBC2815_01135	-2.34	1.21E-05	50S ribosomal protein L32
NBC2815_01136	NBC2815_01136	-1.64	3.57E-03	3-oxoacyl-(acyl carrier protein) synthase III /EC_number="2.3.1.180
NBC2815_01137	NBC2815_01137	-1.83	6.68E-05	ACP S-malonyltransferase
fabG_2	NBC2815_01138	-1.74	3.67E-04	3-ketoacyl-ACP reductase
acpP	NBC2815_01139	-4.87	0.00E+00	acyl carrier protein
fabF_2	NBC2815_01140	-2.44	1.43E-07	3-oxoacyl-ACP synthase
NBC2815_01142	NBC2815_01142	-1.63	3.01E-03	periplasmic solute-binding protein
NBC2815_01143	NBC2815_01143	-1.70	8.67E-04	DNA polymerase III subunit delta' /EC_number="2.7.7.7
NBC2815_01144	NBC2815_01144	-2.05	5.26E-05	Tfp pilus assembly protein
NBC2815_01146	NBC2815_01146	-1.53	4.32E-03	Hypothetical Protein
NBC2815_01147	NBC2815_01147	1.53	2.67E-02	Hypothetical Protein
NBC2815_01149	NBC2815_01149	-5.38	0.00E+00	Hypothetical Protein
virB6_1	NBC2815_01150	-2.63	1.24E-10	type IV secretion system protein VirB6
NBC2815_01151	NBC2815_01151	-2.34	2.74E-10	Hypothetical Protein
NBC2815_01152	NBC2815_01152	-2.03	7.99E-05	Hypothetical Protein
NBC2815_01153	NBC2815_01153	-2.86	1.95E-12	Hypothetical Protein
NBC2815_01154	NBC2815_01154	-2.25	8.15E-08	Hypothetical Protein
NBC2815_01158	NBC2815_01158	-2.13	7.43E-04	Hypothetical Protein
NBC2815_01159	NBC2815_01159	-2.64	2.46E-04	Hypothetical Protein
NBC2815_01162	NBC2815_01162	2.73	3.28E-08	Hypothetical Protein
NBC2815_01165	NBC2815_01165	-1.79	7.43E-03	Hypothetical Protein
NBC2815_01170	NBC2815_01170	-2.16	1.89E-06	Putative membrane protein
NBC2815_01173	NBC2815_01173	-2.37	3.17E-04	hypothetical protein
NBC2815_01175	NBC2815_01175	-1.82	1.01E-02	hypothetical protein
NBC2815_01179	NBC2815_01179	3.58	1.48E-06	membrane-fusion protein
NBC2815_01180	NBC2815_01180	-1.65	3.22E-02	ABC-type bacteriocin/lantibiotic exporter
NBC2815_01181	NBC2815_01181	-2.55	1.40E-06	hydroxyproline-rich glycoprotein DZ-HRGP
NBC2815_01182	NBC2815_01182	-2.76	1.79E-04	hypothetical protein
NBC2815_01186	NBC2815_01186	-2.03	4.04E-02	hypothetical protein
NBC2815_01187	NBC2815_01187	-3.61	1.07E-05	hypothetical protein
NBC2815_01190	NBC2815_01190	1.98	2.54E-05	hypothetical protein
NBC2815_01192	NBC2815_01192	2.73	3.18E-03	MFS transporter permease
NBC2815_01195	NBC2815_01195	3.84	3.43E-07	Hypothetical Protein
NBC2815_01196	NBC2815_01196	2.31	1.57E-02	hypothetical protein
NBC2815_01197	NBC2815_01197	2.32	2.59E-04	outer protein Q
NBC2815_01198	NBC2815_01198	3.01	4.96E-10	Hypothetical Protein
NBC2815_01201	NBC2815_01201	2.15	1.87E-05	putative sugar kinase
NBC2815_01202	NBC2815_01202	1.94	4.22E-04	glycosyl hydrolase
NBC2815_01203	NBC2815_01203	1.72	1.46E-02	oxidoreductase
NBC2815_01204	NBC2815_01204	4.64	3.59E-15	short chain dehydrogenase
NBC2815_01205	NBC2815_01205	2.24	1.73E-06	hypothetical protein
NBC2815_01206	NBC2815_01206	2.27	1.31E-06	thioredoxin reductase /EC_number="1.8.1.9
NBC2815_01207	NBC2815_01207	2.35	5.85E-08	EAL domain-containing protein
NBC2815_01208	NBC2815_01208	1.70	1.14E-02	Guanosine polyphosphate pyrophosphohydrolase/synthetase
NBC2815_01209	NBC2815_01209	2.70	9.25E-03	Transposase
nudE	NBC2815_01213	-1.51	6.78E-03	ADP-ribose diphosphatase NudE
NBC2815_01214	NBC2815_01214	-1.80	6.73E-05	sulfite synthesis pathway protein
mazG	NBC2815_01215	-1.69	1.71E-04	nucleoside triphosphate pyrophosphohydrolase
NBC2815_01216	NBC2815_01216	-2.00	7.29E-03	membrane protein YnfA
gcvT	NBC2815_01217	-3.09	0.00E+00	glycine cleavage system aminomethyltransferase T
NBC2815_01218	NBC2815_01218	-4.17	8.86E-20	glycine cleavage system protein H
NBC2815_01219	NBC2815_01219	-2.91	2.17E-10	hypothetical protein
bla	NBC2815_01220	-1.84	2.16E-04	beta-lactamase
fadE	NBC2815_01223	3.16	3.28E-10	acyl-CoA dehydrogenase
NBC2815_01224	NBC2815_01224	7.05	9.38E-50	hydrolase, alpha/beta fold family
NBC2815_01227	NBC2815_01227	-1.82	1.04E-02	IS1478 transposase
xopC_2	NBC2815_01231	2.56	6.58E-05	outer protein C
NBC2815_01232	NBC2815_01232	2.65	2.23E-05	IS1480 transposase
NBC2815_01233	NBC2815_01233	4.82	8.42E-11	IS1404 transposase protein B
NBC2815_01234	NBC2815_01234	5.28	3.26E-16	IS1404 transposase
NBC2815_01235	NBC2815_01235	1.84	4.88E-03	Putative secreted protein
NBC2815_01236	NBC2815_01236	3.14	2.13E-09	heat shock protein
gtrB	NBC2815_01238	-2.36	1.78E-07	glucosyl transferase
NBC2815_01240	NBC2815_01240	2.00	4.66E-05	putative outer membrane AsmA family protein
nlpD	NBC2815_01246	3.55	1.61E-08	Membrane protein related to metalloendopeptidase
metA	NBC2815_01247	16.57	3.32E-16	homoserine trans-succinylase
MetA	NBC2815_01247	-5.30	2.25E-09	homoserine trans-succinylase
metB	NBC2815_01248	10.19	9.19E-21	cystathionine gamma-synthase
NBC2815_01249	NBC2815_01249	10.33	4.07E-38	homoserine dehydrogenase
NBC2815_01250	NBC2815_01250	2.44	1.34E-07	alpha/beta hydrolase
NBC2815_01251	NBC2815_01251	2.99	5.64E-05	Hypothetical Protein
sdaA_2	NBC2815_01253	-2.11	1.17E-02	L-serine ammonia-lyase
sdaA_3	NBC2815_01254	-1.67	6.94E-04	L-serine ammonia-lyase
NBC2815_01255	NBC2815_01255	2.29	5.09E-03	putative thiol-disulfide isomerase et thioredoxin; glutaredoxin protein
NBC2815_01259	NBC2815_01259	1.66	1.55E-03	histidine kinase-response regulator hybrid protein
NBC2815_01260	NBC2815_01260	3.81	1.21E-04	transposase
ffh	NBC2815_01265	-1.88	5.87E-05	signal recognition particle protein
rpsP	NBC2815_01270	2.37	2.19E-06	30S ribosomal protein S16
rimM	NBC2815_01271	1.75	1.40E-03	16S rRNA-processing protein RimM
trmD	NBC2815_01272	1.94	5.46E-05	tRNA (guanine-N(1)-)-methyltransferase
NBC2815_01276	NBC2815_01276	-1.53	2.54E-03	IS1478 transposase

NBC2815_01277	NBC2815_01277	3.05	1.01E-02	transposase
NBC2815_01279	NBC2815_01279	2.09	1.55E-03	hypothetical protein
NBC2815_01280	NBC2815_01280	2.07	2.02E-04	GGDEF domain-containing protein
NBC2815_01281	NBC2815_01281	4.08	1.55E-10	sensor histidine kinase
NBC2815_01282	NBC2815_01282	8.12	1.52E-06	sensor protein
pheT	NBC2815_01289	-1.65	1.02E-03	phenylalanyl-tRNA ligase subunit beta
ihfA	NBC2815_01290	-3.67	1.32E-15	integration host factor subunit alpha
NBC2815_01291	NBC2815_01291	-2.20	1.47E-04	MerR family transcriptional regulator
gumB	NBC2815_01293	5.62	2.08E-20	protein GumB
gumC	NBC2815_01294	3.42	1.22E-10	xanthan chain-length determinant
gumD	NBC2815_01295	3.59	2.27E-10	protein GumD
NBC2815_01296	NBC2815_01296	1.73	1.97E-03	GumE protein
NBC2815_01302	NBC2815_01302	-1.98	3.73E-05	GumK protein
NBC2815_01303	NBC2815_01303	-3.09	1.15E-14	GumL protein
gumM	NBC2815_01304	-3.24	1.38E-16	GumM protein
NBC2815_01305	NBC2815_01305	-2.14	3.86E-04	Hypothetical Protein
NBC2815_01315	NBC2815_01315	-1.76	4.97E-03	Hypothetical Protein
NBC2815_01317	NBC2815_01317	-2.88	1.50E-06	Fe(II) trafficking protein YggX
NBC2815_01320	NBC2815_01320	-2.19	1.05E-04	transcriptional regulator
NBC2815_01321	NBC2815_01321	-2.31	1.42E-08	proline racemase
NBC2815_01322	NBC2815_01322	-1.58	9.38E-03	D-amino acid oxidase
NBC2815_01325	NBC2815_01325	-2.69	6.05E-11	dihydrodipicolinate synthetase
NBC2815_01328	NBC2815_01328	-2.39	4.02E-09	lipoprotein
NBC2815_01329	NBC2815_01329	-2.82	3.96E-12	Hypothetical Protein
NBC2815_01330	NBC2815_01330	-1.98	6.21E-06	amino acid permease
NBC2815_01331	NBC2815_01331	-3.26	1.70E-14	peptidase
lacA_1	NBC2815_01332	-2.79	8.39E-07	Beta-galactosidase
NBC2815_01333	NBC2815_01333	-1.74	2.58E-04	ISXo1 transposase, IS5 family
NBC2815_01337	NBC2815_01337	-2.66	2.16E-11	peptidase
NBC2815_01338	NBC2815_01338	-1.97	1.88E-06	Hypothetical Protein
NBC2815_01339	NBC2815_01339	-1.75	7.30E-04	putative TonB-dependent transporter
xopAD_3	NBC2815_01341	2.00	5.04E-06	type III effector protein XopAD
xsa_2	NBC2815_01343	2.82	1.25E-04	xylosidase
NBC2815_01344	NBC2815_01344	2.02	3.63E-03	hypothetical protein
NBC2815_01347	NBC2815_01347	-2.90	2.21E-02	hypothetical protein
NBC2815_01353	NBC2815_01353	5.51	1.17E-23	SAM-dependent methyltransferase
NBC2815_01355	NBC2815_01355	-1.84	2.48E-05	acetyl-CoA carboxylase carboxyltransferase subunit alpha /EC_number="6.4.1.2
dnaE	NBC2815_01356	-1.55	6.34E-03	DNA polymerase III subunit alpha
fabZ	NBC2815_01361	-1.68	2.34E-03	(3R)-hydroxymyristoyl-ACP dehydratase
NBC2815_01363	NBC2815_01363	9.83	4.80E-08	Hypothetical Protein
NBC2815_01364	NBC2815_01364	-2.02	1.14E-04	outer membrane antigen
NBC2815_01367	NBC2815_01367	-2.44	2.69E-10	phosphatidate cytidylyltransferase
NBC2815_01368	NBC2815_01368	-2.05	6.81E-07	undecaprenyl pyrophosphate synthase
frr	NBC2815_01369	-2.55	9.18E-09	ribosome recycling factor
pyrH	NBC2815_01370	-2.71	4.57E-10	putative uridylate kinase
tsf	NBC2815_01372	1.64	1.01E-02	elongation factor ts (ef-ts) protein
rpsB	NBC2815_01373	1.66	5.81E-03	30S ribosomal protein S2
NBC2815_01374	NBC2815_01374	5.56	3.39E-24	pili assembly chaperone
NBC2815_01375	NBC2815_01375	1.82	4.83E-04	Hypothetical Protein
NBC2815_01376	NBC2815_01376	2.71	2.84E-08	spore coat U domain protein
NBC2815_01377	NBC2815_01377	4.53	4.52E-15	PapC family outer membrane usher protein
NBC2815_01378	NBC2815_01378	4.81	5.62E-10	pili assembly chaperone
pru	NBC2815_01379	5.88	2.44E-09	protein U
NBC2815_01380	NBC2815_01380	1.81	6.00E-04	methionine aminopeptidase
glnD	NBC2815_01381	2.45	3.31E-07	PII uridylyl-transferase
asnB	NBC2815_01385	2.24	2.29E-06	asparagine synthetase B
NBC2815_01386	NBC2815_01386	1.83	2.94E-02	Hypothetical Protein
NBC2815_01388	NBC2815_01388	1.68	2.35E-03	penicillin acylase II
bfr	NBC2815_01392	-2.98	1.03E-10	bacterioferritin
NBC2815_01394	NBC2815_01394	-3.55	0.00E+00	DNA topoisomerase IV subunit A /EC_number="5.99.1.-
yybA	NBC2815_01395	1.86	7.57E-03	MarR family transcriptional regulator
NBC2815_01398	NBC2815_01398	-2.62	4.56E-05	multidrug resistance membrane translocase
NBC2815_01405	NBC2815_01405	2.08	7.87E-04	Hypothetical Protein
NBC2815_01407	NBC2815_01407	2.24	2.52E-06	lipoprotein
NBC2815_01408	NBC2815_01408	1.82	2.20E-03	MarR family transcriptional regulator
dcp2_1	NBC2815_01409	1.99	1.87E-05	peptidyl-dipeptidase
NBC2815_01412	NBC2815_01412	3.55	4.76E-16	ABC transporter ATP-binding protein
fumB	NBC2815_01413	-1.60	1.20E-03	fumarate hydratase
NBC2815_01418	NBC2815_01418	2.08	5.37E-04	Hypothetical Protein
NBC2815_01420	NBC2815_01420	-1.54	1.68E-02	cold-shock protein
pcp	NBC2815_01421	2.26	6.37E-06	peptidoglycan-associated outer membrane lipoprotein
NBC2815_01422	NBC2815_01422	1.86	1.17E-02	Hypothetical Protein
NBC2815_01426	NBC2815_01426	5.25	5.90E-17	Hypothetical Protein
NBC2815_01427	NBC2815_01427	4.50	9.34E-12	transglutaminase
NBC2815_01430	NBC2815_01430	1.73	3.27E-04	flavoprotein
dnaB	NBC2815_01431	-2.88	5.28E-13	replicative DNA helicase
NBC2815_01433	NBC2815_01433	2.10	4.38E-04	OmpA family protein
NBC2815_01435	NBC2815_01435	5.60	8.03E-14	ISXac3 transposase
NBC2815_01436	NBC2815_01436	2.22	2.30E-02	ISxac3 transposase
NBC2815_01439	NBC2815_01439	2.51	5.47E-05	transcriptional regulator
NBC2815_01440	NBC2815_01440	2.68	8.77E-10	Hypothetical Protein
NBC2815_01441	NBC2815_01441	1.66	1.10E-03	putative DNA-binding protein
NBC2815_01442	NBC2815_01442	-7.59	8.49E-30	aldehyde dehydrogenase
NBC2815_01443	NBC2815_01443	-7.80	5.82E-24	acyl-CoA dehydrogenase
NBC2815_01444	NBC2815_01444	-7.25	4.85E-26	enoyl-CoA hydratase /EC_number="4.2.1.17
NBC2815_01445	NBC2815_01445	-6.27	3.35E-25	enoyl-CoA hydratase
NBC2815_01446	NBC2815_01446	-6.32	3.45E-23	3-hydroxyisobutyrate dehydrogenase
NBC2815_01450	NBC2815_01450	2.06	4.96E-05	regulatory protein
NBC2815_01452	NBC2815_01452	-10.42	4.40E-13	hypothetical protein
corA_1	NBC2815_01458	-1.80	9.17E-05	magnesium and cobalt transport protein
NBC2815_01461	NBC2815_01461	-1.78	4.47E-04	Hypothetical Protein
NBC2815_01462	NBC2815_01462	-2.98	1.73E-13	metal-binding heat shock protein
NBC2815_01465	NBC2815_01465	7.27	4.30E-03	hypothetical protein
NBC2815_01467	NBC2815_01467	-4.22	6.46E-22	ubiquinol cytochrome C oxidoreductase, iron-sulfur subunit

NBC2815_01468	NBC2815_01468	-4.25	9.05E-15	ubiquinol cytochrome C oxidoreductase cytochrome B subunit
petC	NBC2815_01469	-2.91	8.26E-12	ubiquinol cytochrome C oxidoreductase, cytochrome C1 subunit
sspA	NBC2815_01470	-3.12	4.68E-14	stringent starvation protein a
NBC2815_01471	NBC2815_01471	-1.60	4.09E-03	ClpXP protease specificity-enhancing factor
NBC2815_01472	NBC2815_01472	1.90	9.47E-04	Hypothetical Protein
lhr1	NBC2815_01473	3.39	1.51E-11	ATP-dependent DNA helicase
NBC2815_01474	NBC2815_01474	3.08	3.49E-13	3-methyladenine DNA glycosylase
NBC2815_01477	NBC2815_01477	-2.97	6.03E-11	virulence regulator
NBC2815_01485	NBC2815_01485	-2.44	7.36E-08	signal peptidase I
NBC2815_01486	NBC2815_01486	-3.94	6.27E-14	Hypothetical Protein
era	NBC2815_01488	-2.32	1.90E-08	GTP-binding protein Era
recO	NBC2815_01489	-1.54	3.71E-02	DNA repair protein RecO
rumA	NBC2815_01491	-3.00	3.09E-11	23S rRNA 5-methyluridine methyltransferase
NBC2815_01492	NBC2815_01492	-3.21	2.59E-08	adenylate cyclase /EC_number="4.6.1.1
deoD	NBC2815_01497	-1.73	1.48E-04	5'-methylthioadenosine phosphorylase
scoF	NBC2815_01498	-4.38	3.14E-17	cold shock protein
NBC2815_01500	NBC2815_01500	1.68	7.62E-04	helicase-like protein
NBC2815_01501	NBC2815_01501	2.61	1.33E-08	ATP-dependent DNA ligase /EC_number="6.5.1.1
NBC2815_01502	NBC2815_01502	3.60	6.98E-09	hypothetical protein
NBC2815_01503	NBC2815_01503	3.53	3.11E-13	mRNA 3'-end processing factor
NBC2815_01504	NBC2815_01504	5.44	1.12E-34	Hypothetical Protein
NBC2815_01505	NBC2815_01505	4.35	1.89E-14	Hypothetical Protein
NBC2815_01506	NBC2815_01506	4.46	4.09E-15	lipoprotein
NBC2815_01508	NBC2815_01508	-1.66	1.40E-03	acetoacetyl-CoA thiolase
NBC2815_01509	NBC2815_01509	-2.80	4.18E-06	ISXo1 transposase, IS5 family
NBC2815_01510	NBC2815_01510	1.80	1.19E-04	leucine dehydrogenase
NBC2815_01511	NBC2815_01511	2.71	6.86E-06	Hypothetical Protein
NBC2815_01513	NBC2815_01513	3.41	1.59E-11	Hypothetical Protein
NBC2815_01514	NBC2815_01514	-1.91	2.26E-04	Hypothetical Protein
NBC2815_01515	NBC2815_01515	-2.12	5.33E-03	transposase
NBC2815_01517	NBC2815_01517	-1.79	3.05E-05	ATPase
dniR_2	NBC2815_01518	-2.27	2.12E-09	membrane-bound lytic murein transglycosylase D
NBC2815_01519	NBC2815_01519	-2.03	1.65E-05	protein FimV
NBC2815_01520	NBC2815_01520	-1.56	8.95E-03	glyoxalase-bleomycin resistance protein-dioxygenase superfamily protein
truA	NBC2815_01521	1.89	3.61E-05	tRNA pseudouridine synthase A
NBC2815_01522	NBC2815_01522	2.33	2.73E-07	N-(5'-phosphoribosyl)anthranilate isomerase /EC_number="5.3.1.24
NBC2815_01525	NBC2815_01525	-1.59	4.02E-02	ISXo1 transposase, IS5 family
NBC2815_01527	NBC2815_01527	1.90	2.13E-03	transcriptional regulator
accD	NBC2815_01531	-1.64	3.38E-03	Acyl-CoA carboxyltransferase beta chain
glmM	NBC2815_01532	-1.88	4.13E-05	phosphoglucosamine mutase
NBC2815_01533	NBC2815_01533	-1.69	4.31E-04	oxidoreductase
tpiA	NBC2815_01539	-1.90	1.55E-05	triosephosphate isomerase
nuoA	NBC2815_01542	-1.90	6.10E-05	NADH dehydrogenase subunit A
nuoB	NBC2815_01543	-2.13	3.13E-06	NADH dehydrogenase subunit B
NBC2815_01544	NBC2815_01544	-2.28	2.19E-07	NADH dehydrogenase subunit C /EC_number="1.6.5.11
NBC2815_01545	NBC2815_01545	-3.01	7.10E-12	NADH dehydrogenase subunit D /EC_number="1.6.5.11
nuoE	NBC2815_01546	-2.98	1.52E-11	NADH-quinone oxidoreductase chain e (nadh dehydrogenaseI chain e) (ndh-1, chain e) (nuo5) oxidoreductase
nuoF	NBC2815_01547	-3.07	2.88E-12	NADH-quinone oxidoreductase chain f (nadh dehydrogenaseI chain f) (ndh-1, chain f) (nuo6) oxidoreductase
NBC2815_01548	NBC2815_01548	-2.87	1.08E-10	NADH dehydrogenase subunit G /EC_number="1.6.5.3
nuoH	NBC2815_01549	-3.25	1.04E-13	NADH-quinone oxidoreductase subunit h (nadh dehydrogenaseIsubunit h) (ndh-1 subunit h) (nuo8) oxidoreductase
NBC2815_01550	NBC2815_01550	-2.92	1.14E-12	NADH dehydrogenase subunit I /EC_number="1.6.5.3
nuoJ	NBC2815_01551	-2.64	2.66E-11	NADH dehydrogenase subunit J
nuoK	NBC2815_01552	-3.44	9.20E-18	NADH-quinone oxidoreductase chain k (nadh dehydrogenaseI chain k) (ndh-1, chain k) (nuo11) oxidoreductase
NBC2815_01553	NBC2815_01553	-3.04	4.13E-11	NADH dehydrogenase subunit L
NBC2815_01554	NBC2815_01554	-3.77	0.00E+00	NADH:ubiquinone oxidoreductase subunit M /EC_number="1.6.5.11
NBC2815_01555	NBC2815_01555	-5.06	0.00E+00	NADH-ubiquinone oxidoreductase Nqo14 subunit
NBC2815_01557	NBC2815_01557	-1.63	8.54E-04	ribosome maturation protein RimP
nusA	NBC2815_01558	-1.56	6.38E-03	transcription elongation protein nusa
infB	NBC2815_01559	-1.76	1.13E-03	translation initiation factor IF-2
rpsO	NBC2815_01562	-1.65	1.93E-03	30S ribosomal protein S15
NBC2815_01563	NBC2815_01563	-2.49	4.44E-08	polynucleotide phosphorylase /EC_number="2.7.7.8
NBC2815_01564	NBC2815_01564	37.34	1.25E-02	transposase
NBC2815_01566	NBC2815_01566	-2.89	2.03E-13	nicotinate-nucleotide pyrophosphorylase
NBC2815_01567	NBC2815_01567	-5.23	2.48E-16	Hypothetical Protein
purE	NBC2815_01568	-2.71	1.83E-13	N5-carboxyaminoimidazole ribonucleotide mutase
NBC2815_01569	NBC2815_01569	-2.52	6.95E-11	phosphoribosylaminoimidazole carboxylase ATPase subunit /EC_number="4.1.1.21
NBC2815_01570	NBC2815_01570	-1.85	4.59E-03	iron superoxide dismutase
NBC2815_01571	NBC2815_01571	-3.46	8.48E-10	glutaredoxin-like protein
NBC2815_01573	NBC2815_01573	-3.48	1.01E-06	Integral membrane protein of the MarC family protein
NBC2815_01575	NBC2815_01575	-2.34	2.26E-09	Rossmann fold nucleotide-binding protein
NBC2815_01576	NBC2815_01576	-3.35	3.76E-16	alginate biosynthesis protein
NBC2815_01577	NBC2815_01577	-6.68	4.62E-30	pre-pilin like leader sequence
pilW	NBC2815_01578	-30.62	1.15E-56	type IV pilus assembly protein PilW
NBC2815_01579	NBC2815_01579	-21.61	0.00E+00	PilY1 protein
pilE	NBC2815_01580	-12.95	8.30E-39	type IV pilin pile protein
NBC2815_01581	NBC2815_01581	-2.79	2.13E-13	IS1478 transposase
NBC2815_01586	NBC2815_01586	-2.45	2.12E-09	DNA helicase-like protein
NBC2815_01588	NBC2815_01588	2.18	2.85E-03	beta alanine--pyruvate transaminase /EC_number="2.6.1.18
NBC2815_01589	NBC2815_01589	-3.29	2.99E-16	integral membrane transporter
NBC2815_01590	NBC2815_01590	-1.55	1.50E-02	integral membrane transporter
yhjE	NBC2815_01594	-3.34	3.69E-03	metabolite transport protein
NBC2815_01598	NBC2815_01598	1.94	2.00E-05	oxidoreductase
NBC2815_01600	NBC2815_01600	-1.70	1.24E-03	glutamine synthetase
gabD	NBC2815_01610	2.08	3.55E-04	succinate-semialdehyde dehydrogenase
NBC2815_01614	NBC2815_01614	1.64	3.76E-03	multidrug resistance protein
NBC2815_01615	NBC2815_01615	3.20	1.06E-11	TetR/AcrR family transcriptional regulator
NBC2815_01616	NBC2815_01616	-2.17	3.18E-06	NAD-specific glutamate dehydrogenase /EC_number="1.4.1.2
NBC2815_01618	NBC2815_01618	-1.80	8.95E-04	transposase
NBC2815_01620	NBC2815_01620	2.47	1.20E-07	tis1421-transposase b
NBC2815_01621	NBC2815_01621	3.82	2.89E-07	HrpF protein
NBC2815_01622	NBC2815_01622	2.13	2.34E-07	IS1478 transposase
NBC2815_01626	NBC2815_01626	2.76	3.85E-07	putative replication protein
NBC2815_01627	NBC2815_01627	3.03	2.86E-04	hypothetical protein
NBC2815_01630	NBC2815_01630	4.94	1.82E-06	cytochrome D ubiquinol oxidase subunit I

NBC2815_01631	NBC2815_01631	-3.41	1.58E-04	ISXo1 transposase, IS5 family
NBC2815_01635	NBC2815_01635	4.02	7.94E-05	ISXac3 transposase, IS3 family
NBC2815_01639	NBC2815_01639	2.84	1.20E-08	Hypothetical Protein
NBC2815_01642	NBC2815_01642	2.17	1.27E-03	VirB8 protein
NBC2815_01646	NBC2815_01646	3.65	7.24E-12	VirB1 protein
NBC2815_01648	NBC2815_01648	1.67	5.50E-04	VirB3 protein
virB4	NBC2815_01649	2.92	1.02E-09	VirB4 protein
NBC2815_01651	NBC2815_01651	-2.21	2.59E-05	VirB6 protein
NBC2815_01652	NBC2815_01652	-4.31	2.76E-17	Hypothetical Protein
NBC2815_01653	NBC2815_01653	-3.46	5.08E-20	carboxypeptidase
NBC2815_01654	NBC2815_01654	-1.89	2.84E-03	ISXo1 transposase, IS5 family
NBC2815_01658	NBC2815_01658	3.07	1.56E-13	serine peptidase
NBC2815_01659	NBC2815_01659	2.03	4.18E-03	subtilase serine protease
NBC2815_01660	NBC2815_01660	3.30	6.01E-11	subtilase serine protease
NBC2815_01662	NBC2815_01662	-1.73	1.35E-04	Oligoketide cyclase/lipid transport protein
fur	NBC2815_01665	-1.58	3.24E-03	ferric uptake regulator Fur
hrcA	NBC2815_01667	2.23	1.99E-04	heat-inducible transcription repressor
NBC2815_01669	NBC2815_01669	2.35	5.46E-05	heat shock protein GrpE
dnaK	NBC2815_01670	1.73	1.25E-02	molecular chaperone DnaK
NBC2815_01671	NBC2815_01671	2.49	2.18E-05	molecular chaperone DnaJ
pdxY	NBC2815_01672	2.08	5.89E-05	pyridoxine kinase
NBC2815_01674	NBC2815_01674	1.69	1.46E-02	Hypothetical Protein
NBC2815_01676	NBC2815_01676	1.57	9.62E-03	RND efflux membrane fusion protein
NBC2815_01678	NBC2815_01678	1.94	4.91E-02	putative LysR family transcriptional regulator
NBC2815_01687	NBC2815_01687	-2.33	1.34E-07	cupin superfamily protein, putative
NBC2815_01688	NBC2815_01688	-2.07	7.86E-07	adenylosuccinate lyase /EC_number="4.3.2.2
NBC2815_01690	NBC2815_01690	-1.75	3.56E-03	IS1479 transposase-like protein
NBC2815_01693	NBC2815_01693	-1.77	2.21E-03	lipoprotein
NBC2815_01694	NBC2815_01694	3.12	9.81E-05	IS1480 transposase
NBC2815_01697	NBC2815_01697	-2.17	4.24E-03	hypothetical protein
NBC2815_01702	NBC2815_01702	-2.45	5.95E-10	ABC transporter vitamin B12 uptake permease
fkpA	NBC2815_01703	-1.80	3.04E-04	FKBP-type peptidylprolyl isomerase
slyX	NBC2815_01705	-1.91	4.90E-05	Hypothetical Protein
NBC2815_01706	NBC2815_01706	-1.98	3.03E-05	nucleoprotein/polynucleotide-associated enzyme
NBC2815_01707	NBC2815_01707	-2.04	1.01E-07	Hypothetical Protein
NBC2815_01709	NBC2815_01709	-3.13	1.97E-13	glucose-galactose transporter
NBC2815_01710	NBC2815_01710	-2.41	9.05E-09	fructokinase
NBC2815_01711	NBC2815_01711	-2.80	6.32E-10	N-acyl-D-glucosamine 2-epimerase
NBC2815_01712	NBC2815_01712	2.73	5.60E-08	5-methyltetrahydrofolate--homocysteine methyltransferase
NBC2815_01713	NBC2815_01713	3.18	5.30E-08	5-methyltetrahydrofolate--homocysteine methyltransferase
NBC2815_01714	NBC2815_01714	3.05	6.44E-07	transcriptional regulator
acdA	NBC2815_01715	1.87	4.94E-05	acyl-CoA dehydrogenase
NBC2815_01716	NBC2815_01716	-2.05	1.90E-06	acetylhydrolase
NBC2815_01718	NBC2815_01718	-1.72	8.55E-04	RNase R
NBC2815_01720	NBC2815_01720	-2.25	1.76E-06	tRNA/rRNA methyltransferase
NBC2815_01721	NBC2815_01721	8.05	1.76E-05	periplasmic ligand-binding sensor domain-containing protein
NBC2815_01730	NBC2815_01730	1.84	8.74E-05	Hypothetical Protein
NBC2815_01741	NBC2815_01741	-1.74	1.17E-02	N-acetylmuramyl-L-alanine amidase, negative regulator of AmpC, AmpD
NBC2815_01744	NBC2815_01744	1.71	2.65E-02	acetyltransferase
NBC2815_01749	NBC2815_01749	-2.47	1.01E-04	ISXo1 transposase, IS5 family
yadF	NBC2815_01757	-2.93	3.03E-14	carbonic anhydrase
NBC2815_01759	NBC2815_01759	-2.40	1.00E-05	Hypothetical Protein
NBC2815_01760	NBC2815_01760	-2.50	5.41E-05	Hypothetical Protein
asnC	NBC2815_01762	-2.67	2.82E-10	asparaginyl-tRNA ligase
rpsR	NBC2815_01765	2.02	2.70E-05	30S ribosomal protein S18
ligA	NBC2815_01769	-1.79	5.56E-05	NAD-dependent DNA ligase LigA
NBC2815_01770	NBC2815_01770	-1.96	7.67E-04	lysyl-tRNA synthetase
NBC2815_01771	NBC2815_01771	1.58	2.51E-03	Hypothetical Protein
NBC2815_01774	NBC2815_01774	1.84	1.54E-03	hypothetical protein
hutC	NBC2815_01780	2.84	1.27E-12	GntR family transcriptional regulator
phaF	NBC2815_01781	1.73	1.69E-03	poly(hydroxyalcanoate) granule associated protein
NBC2815_01782	NBC2815_01782	-2.21	1.50E-02	putative polyhydroxyalkanoic acid system protein
psd	NBC2815_01785	-2.26	1.13E-08	phosphatidylserine decarboxylase
NBC2815_01786	NBC2815_01786	-2.46	3.88E-09	SCO1/SenC family protein
NBC2815_01787	NBC2815_01787	-1.80	3.85E-05	N5-glutamine S-adenosyl-L-methionine-dependent methyltransferase
NBC2815_01788	NBC2815_01788	-2.04	2.74E-04	hypothetical protein
NBC2815_01789	NBC2815_01789	-1.59	1.32E-03	chorismate synthase /EC_number="4.2.3.5
NBC2815_01790	NBC2815_01790	-1.75	2.49E-04	2-hydroxyacid dehydrogenase
NBC2815_01791	NBC2815_01791	-2.70	7.42E-11	aspartate-semialdehyde dehydrogenase /EC_number="1.2.1.11
NBC2815_01792	NBC2815_01792	1.66	3.86E-02	hypothetical protein
NBC2815_01793	NBC2815_01793	4.34	3.37E-08	chemotaxis protein
NBC2815_01795	NBC2815_01795	2.52	2.11E-07	recombinase A
NBC2815_01796	NBC2815_01796	2.53	9.07E-09	histidine kinase-response regulator hybrid protein
NBC2815_01798	NBC2815_01798	2.88	4.82E-04	IS1404 transposase protein B
NBC2815_01799	NBC2815_01799	4.19	2.97E-05	IS1404 transposase
NBC2815_01800	NBC2815_01800	2.73	1.00E-04	cation efflux system protein
NBC2815_01801	NBC2815_01801	22.60	3.73E-35	autotransporter serine protease
NBC2815_01804	NBC2815_01804	1.61	6.00E-03	tis1421-transposase b
xopAD_4	NBC2815_01805	1.90	7.10E-04	type III effector protein XopAD
NBC2815_01808	NBC2815_01808	1.98	6.20E-06	L-sorbosone dehydrogenase
NBC2815_01809	NBC2815_01809	3.30	2.92E-06	IS1478 transposase
NBC2815_01811	NBC2815_01811	4.56	6.64E-03	Hypothetical Protein
NBC2815_01812	NBC2815_01812	4.45	2.04E-04	Hypothetical Protein
NBC2815_01814	NBC2815_01814	3.20	3.00E-07	Hypothetical Protein
NBC2815_01815	NBC2815_01815	29.02	4.74E-26	chemotaxis protein
NBC2815_01816	NBC2815_01816	21.42	6.80E-23	chemotaxis phosphatase CheZ
NBC2815_01817	NBC2815_01817	20.24	1.46E-20	chemotaxis response regulator chey protein
NBC2815_01818	NBC2815_01818	43.42	1.32E-30	RNA polymerase sigma factor
NBC2815_01819	NBC2815_01819	24.85	2.05E-28	flagellar biosynthesis switch protein
flhF	NBC2815_01820	62.36	1.37E-42	flagellar gtp-binding protein flhf
flhA	NBC2815_01821	13.80	1.47E-21	flagellar biosynthesis protein FlhA
flhB	NBC2815_01822	10.37	7.72E-14	flagellar biosynthesis protein FlhB
NBC2815_01823	NBC2815_01823	26.41	1.01E-24	GGDEF domain-containing protein
NBC2815_01824	NBC2815_01824	3.90	3.63E-14	diguanylate cyclase

NBC2815_01825	NBC2815_01825	13.56	1.37E-20	flagellar biosynthetic protein FliR
NBC2815_01826	NBC2815_01826	20.99	8.05E-20	flagellar biosynthesis
fliP	NBC2815_01827	4.65	3.48E-17	flagellar biosynthesis protein FliP
fliO	NBC2815_01828	7.53	9.43E-17	flagellar protein
NBC2815_01829	NBC2815_01829	6.76	3.44E-14	flagellar protein
fliM	NBC2815_01830	8.63	6.71E-17	flagellar motor switch protein FliM
fliL	NBC2815_01831	11.02	9.54E-17	flagellar basal body-associated protein FliL
NBC2815_01832	NBC2815_01832	7.93	2.22E-12	flagellar protein
NBC2815_01833	NBC2815_01833	6.59	1.42E-10	flagellar biosynthesis chaperone FliJ
NBC2815_01834	NBC2815_01834	8.23	3.40E-13	flagellar protein
NBC2815_01835	NBC2815_01835	7.22	3.25E-14	flagellar protein
fliG	NBC2815_01836	4.27	2.94E-09	flagellar protein
fliF	NBC2815_01837	5.40	7.24E-12	flagellar M-ring protein FliF
fliE	NBC2815_01838	5.55	3.08E-08	flagellar protein
NBC2815_01839	NBC2815_01839	1.61	1.24E-03	O-antigen biosynthesis protein
rpoN2	NBC2815_01844	2.95	4.21E-06	RNA polymerase sigma-54 factor
NBC2815_01845	NBC2815_01845	2.23	3.69E-06	LuxR family two-component response regulator
NBC2815_01846	NBC2815_01846	24.94	3.40E-23	Hypothetical Protein
NBC2815_01847	NBC2815_01847	26.61	1.45E-18	Hypothetical Protein
NBC2815_01848	NBC2815_01848	40.62	5.26E-25	flagellar protein
NBC2815_01849	NBC2815_01849	28.26	3.41E-21	flagellar protein
NBC2815_01850	NBC2815_01850	43.38	3.10E-22	flagellar protein
flgL	NBC2815_01851	8.56	1.27E-14	flagellar hook-associated protein FlgL
flgK	NBC2815_01852	5.96	9.47E-12	flagellar hook-associated protein FlgK
flgJ	NBC2815_01853	37.95	1.37E-34	flagellar rod assembly protein/muramidase FlgJ
flgI	NBC2815_01854	32.73	1.52E-43	flagellar basal body P-ring protein
flgH	NBC2815_01855	21.63	5.73E-24	flagellar basal body L-ring protein
flgG	NBC2815_01856	29.92	2.80E-23	flagellar basal body rod protein FlgG
flgF	NBC2815_01857	32.11	4.75E-25	flagellar basal body rod protein FlgF
flgE	NBC2815_01858	35.57	3.92E-25	flagellar hook protein FlgE
flgD	NBC2815_01859	22.01	5.30E-25	flagellar basal body rod modification protein
flgC	NBC2815_01860	20.42	7.19E-27	flagellar basal body rod protein FlgC
flgB	NBC2815_01861	23.53	9.87E-40	flagellar basal body rod protein FlgB
NBC2815_01862	NBC2815_01862	18.84	1.99E-18	chemotaxis signal transduction protein
flgA	NBC2815_01863	19.11	2.28E-25	flagellar basal body P-ring biosynthesis protein FlgA
NBC2815_01864	NBC2815_01864	28.59	6.86E-23	negative regulator of flagellin synthesis
NBC2815_01865	NBC2815_01865	29.02	1.47E-21	Hypothetical Protein
NBC2815_01869	NBC2815_01869	8.89	3.10E-21	signal transduction protein
NBC2815_01870	NBC2815_01870	4.48	6.89E-11	signal transduction protein
NBC2815_01871	NBC2815_01871	4.53	9.51E-04	hypothetical protein
NBC2815_01872	NBC2815_01872	5.90	3.52E-07	Hypothetical Protein
NBC2815_01873	NBC2815_01873	10.29	6.92E-13	methyl-accepting chemotaxis protein
clpA	NBC2815_01878	5.13	1.21E-15	ATP-dependent clp protease ATP-binding subunit Clpa protein
aat	NBC2815_01880	2.97	4.97E-08	leucyl/phenylalanyl-tRNA--protein transferase
NBC2815_01881	NBC2815_01881	1.56	9.61E-03	Hypothetical Protein
ftsK	NBC2815_01883	2.17	1.17E-05	DNA translocase FtsK
NBC2815_01884	NBC2815_01884	1.96	4.88E-03	hypothetical protein
NBC2815_01885	NBC2815_01885	3.19	6.14E-12	putative secreted protein
lolA	NBC2815_01886	1.91	1.21E-04	Outer membrane lipoprotein-sorting protein
NBC2815_01890	NBC2815_01890	1.76	2.21E-04	VWA containing CoxE-like protein
NBC2815_01892	NBC2815_01892	-1.71	1.13E-03	3-hydroxyacyl-CoA dehydrogenase
NBC2815_01893	NBC2815_01893	-1.82	1.35E-04	TetR family transcriptional regulator
NBC2815_01900	NBC2815_01900	-1.71	4.34E-04	GTP-binding protein Der
NBC2815_01901	NBC2815_01901	-1.63	5.14E-03	molybdopterin biosynthesis
NBC2815_01903	NBC2815_01903	-1.76	2.86E-04	TonB-dependent receptor
NBC2815_01908	NBC2815_01908	1.61	3.46E-02	siroheme synthase
NBC2815_01910	NBC2815_01910	-1.89	2.32E-02	ISXo1 transposase, IS5 family
NBC2815_01912	NBC2815_01912	1.56	2.82E-02	IS1404 transposase
NBC2815_01913	NBC2815_01913	2.35	7.54E-08	integrase
NBC2815_01915	NBC2815_01915	-2.19	4.65E-05	Hypothetical Protein
NBC2815_01916	NBC2815_01916	2.83	1.37E-05	hypothetical protein
NBC2815_01919	NBC2815_01919	1.98	5.42E-05	VGR-like protein
lysM	NBC2815_01921	-1.66	1.07E-03	LysM domain-containing protein
NBC2815_01922	NBC2815_01922	3.75	1.51E-16	VGR-like protein
NBC2815_01923	NBC2815_01923	2.04	1.57E-04	hypothetical protein
NBC2815_01924	NBC2815_01924	3.58	3.95E-10	EF hand domain-containing protein
NBC2815_01925	NBC2815_01925	1.69	4.88E-02	VGR-like protein
NBC2815_01926	NBC2815_01926	-2.75	5.46E-11	hypothetical protein
NBC2815_01927	NBC2815_01927	-1.64	1.54E-03	hypothetical protein
NBC2815_01928	NBC2815_01928	-1.57	9.21E-03	hypothetical protein
NBC2815_01931	NBC2815_01931	1.67	5.10E-03	VGR-like protein
impJ	NBC2815_01933	1.54	1.51E-02	protein ImpJ
NBC2815_01934	NBC2815_01934	2.93	2.26E-10	transmembrane protein
NBC2815_01941	NBC2815_01941	1.58	7.93E-03	putative EvpG protein, Type VI secretion system
NBC2815_01946	NBC2815_01946	-4.62	6.94E-22	hypothetical protein
NBC2815_01947	NBC2815_01947	-1.75	6.04E-04	hypothetical protein
NBC2815_01948	NBC2815_01948	1.96	4.36E-06	VGR-like protein
NBC2815_01950	NBC2815_01950	-2.04	6.63E-05	ISxac3 transposase
NBC2815_01952	NBC2815_01952	-3.14	1.27E-07	ISXo1 transposase, IS5 family
NBC2815_01956	NBC2815_01956	-2.33	4.27E-02	pyridine nucleotide-disulfide oxidoreductase
NBC2815_01957	NBC2815_01957	-1.89	1.27E-02	FKBP-type peptidylprolyl isomerase
NBC2815_01958	NBC2815_01958	-1.92	4.99E-02	Hypothetical Protein
NBC2815_01959	NBC2815_01959	-1.97	1.10E-02	rhodanese superfamily protein
NBC2815_01964	NBC2815_01964	-1.86	2.65E-02	hypothetical protein
NBC2815_01966	NBC2815_01966	-3.34	4.31E-02	IS1480 transposase
NBC2815_01967	NBC2815_01967	1.62	2.99E-02	zinc transporter ZupT
NBC2815_01968	NBC2815_01968	1.54	2.21E-02	zinc transporter ZupT
NBC2815_01969	NBC2815_01969	1.98	3.21E-02	ISXac3 transposase, IS3 family
NBC2815_01971	NBC2815_01971	2.54	2.17E-06	zinc transporter ZupT
NBC2815_01973	NBC2815_01973	-1.76	8.76E-04	pterin-4-alpha-carbinolamine dehydratase /EC_number="4.2.1.96
NBC2815_01974	NBC2815_01974	1.87	6.55E-05	protein GntY
NBC2815_01975	NBC2815_01975	1.51	6.76E-03	Hypothetical Protein
NBC2815_01985	NBC2815_01985	3.66	2.87E-13	hypothetical protein
NBC2815_01986	NBC2815_01986	-1.74	3.55E-02	hypothetical protein

NBC2815_01988	NBC2815_01988	2.38	2.31E-08	phage replication protein RstA
NBC2815_01989	NBC2815_01989	3.59	1.21E-02	hypothetical protein
NBC2815_01991	NBC2815_01991	2.17	4.33E-04	L-sorbosone dehydrogenase
NBC2815_01992	NBC2815_01992	3.67	1.09E-05	L-sorbosone dehydrogenase
motC	NBC2815_01995	17.60	2.04E-20	flagellar motor protein
NBC2815_01996	NBC2815_01996	51.07	5.51E-22	flagellar motor protein MotD
NBC2815_01997	NBC2815_01997	23.68	1.79E-17	ParA family ATPase
NBC2815_01998	NBC2815_01998	28.05	5.46E-19	chemotaxis protein
NBC2815_01999	NBC2815_01999	31.44	2.16E-20	anti-sigma factor antagonist-like protein
cheY1	NBC2815_02000	17.69	9.86E-17	two-component system regulatory protein
cheA	NBC2815_02001	20.29	1.28E-17	Chemotaxis protein histidine kinase
NBC2815_02002	NBC2815_02002	9.77	5.26E-08	Hypothetical Protein
NBC2815_02003	NBC2815_02003	58.87	6.60E-38	Hypothetical Protein
NBC2815_02004	NBC2815_02004	30.70	2.72E-23	chemotaxis protein
NBC2815_02005	NBC2815_02005	12.16	5.12E-17	methyl-accepting chemotaxis protein
tsr6	NBC2815_02006	11.70	2.31E-15	membrane-anchored chemotaxis sensory transducer
tsr	NBC2815_02007	17.87	4.57E-18	chemotaxis protein
NBC2815_02008	NBC2815_02008	2.84	2.62E-07	methyl-accepting chemotaxis protein
NBC2815_02009	NBC2815_02009	14.62	4.59E-17	methyl-accepting chemotaxis protein
pilZ	NBC2815_02010	17.32	6.76E-10	type IV pilus assembly protein PilZ
NBC2815_02011	NBC2815_02011	2.56	1.01E-02	type IV pilus assembly protein PilZ
NBC2815_02012	NBC2815_02012	24.10	2.40E-16	chemotaxis signal transduction protein
NBC2815_02013	NBC2815_02013	16.81	2.08E-10	Hypothetical Protein
NBC2815_02014	NBC2815_02014	39.02	1.96E-22	chemotaxis protein methyltransferase
NBC2815_02015	NBC2815_02015	6.85	4.37E-18	chemoreceptor glutamine deamidase CheD
NBC2815_02016	NBC2815_02016	7.72	2.80E-17	chemotaxis-specific methylesterase /EC_number="3.1.1.61
NBC2815_02017	NBC2815_02017	7.11	2.05E-14	c-di-GMP phosphodiesterase or signal transduction protein
acnA	NBC2815_02022	4.50	3.05E-15	Aconitase A
NBC2815_02023	NBC2815_02023	6.37	1.10E-18	Hypothetical Protein
rpfF	NBC2815_02025	-1.87	1.96E-04	enoyl-CoA hydratase
rpfG	NBC2815_02028	-1.70	1.32E-04	response regulator
lysS	NBC2815_02029	-1.76	1.34E-04	lysyl-tRNA synthetase
prfB	NBC2815_02030	-1.92	3.17E-05	peptide chain release factor 2
carB	NBC2815_02034	-1.76	2.42E-03	carbamoyl-phosphate synthetase large chain protein
NBC2815_02036	NBC2815_02036	-1.75	4.72E-03	hypothetical protein
NBC2815_02038	NBC2815_02038	-1.63	7.27E-04	dihydrodipicolinate reductase /EC_number="1.17.1.8
NBC2815_02039	NBC2815_02039	2.36	9.58E-05	valine-pyruvate aminotransferase
NBC2815_02040	NBC2815_02040	2.83	2.14E-05	Hypothetical Protein
feoB	NBC2815_02041	3.14	5.08E-12	ferrous iron transport protein B
feoA	NBC2815_02042	2.62	8.02E-10	ferrous iron uptake protein
NBC2815_02043	NBC2815_02043	-1.84	1.69E-04	enoyl-CoA hydratase
NBC2815_02044	NBC2815_02044	-1.75	1.43E-03	hydroxymethylglutaryl-CoA lyase
NBC2815_02045	NBC2815_02045	-1.72	1.88E-04	3-hydroxyacyl-CoA dehydrogenase
NBC2815_02046	NBC2815_02046	-2.58	1.42E-11	elongation factor P
NBC2815_02047	NBC2815_02047	1.56	3.67E-03	Hypothetical Protein
NBC2815_02048	NBC2815_02048	1.85	3.75E-04	Hypothetical Protein
NBC2815_02052	NBC2815_02052	-2.49	4.68E-10	cationic amino acid transporter
yhdG_2	NBC2815_02053	-2.66	3.13E-11	cationic amino acid transporter
NBC2815_02054	NBC2815_02054	-2.06	1.23E-07	methylthioribulose-1-phosphate dehydratase
NBC2815_02055	NBC2815_02055	-1.68	2.26E-04	dioxygenase
NBC2815_02056	NBC2815_02056	-1.80	3.04E-04	enolase
NBC2815_02057	NBC2815_02057	-1.57	4.55E-03	Hypothetical Protein
NBC2815_02058	NBC2815_02058	2.08	1.97E-02	IS1478 transposase
NBC2815_02059	NBC2815_02059	1.95	1.90E-04	hypothetical protein
NBC2815_02062	NBC2815_02062	3.07	3.02E-11	imidazole glycerol phosphate synthase subunit HisF /EC_number="4.1.3.-
NBC2815_02063	NBC2815_02063	3.71	1.34E-11	1-(5-phosphoribosyl)-5-[(5- phosphoribosylamino)methylideneamino] imidazole-4-carboxamide isomerase /EC_number="5.3.1.1
hisH	NBC2815_02064	5.75	1.34E-24	imidazole glycerol phosphate synthase subunit HisH
NBC2815_02065	NBC2815_02065	4.71	4.82E-21	imidazole glycerol-phosphate dehydratase/histidinol phosphatase
hisC	NBC2815_02066	6.08	5.11E-29	histidinol-phosphate aminotransferase
hisD	NBC2815_02067	7.70	3.93E-38	bifunctional histidinal dehydrogenase/ histidinol dehydrogenase
hisG	NBC2815_02068	5.76	2.63E-21	ATP phosphoribosyltransferase
NBC2815_02069	NBC2815_02069	7.75	1.70E-09	TrpR-like protein YerC/YecD
NBC2815_02078	NBC2815_02078	-2.21	6.39E-06	putative plasmid stability protein
NBC2815_02083	NBC2815_02083	-6.47	4.29E-14	hypothetical protein
guaA_2	NBC2815_02087	-1.63	1.11E-03	GMP synthase
guaB	NBC2815_02088	-1.64	4.30E-03	inosine-5-monophosphate dehydrogenase
NBC2815_02089	NBC2815_02089	-1.77	3.58E-04	bifunctional 5,10-methylene-tetrahydrofolate dehydrogenase/ 5,10-methylene-tetrahydrofolate cyclohydrolase
cmk	NBC2815_02104	1.65	3.69E-03	cytidylate kinase
NBC2815_02110	NBC2815_02110	-1.53	8.82E-03	pheromone shutdown protein
NBC2815_02113	NBC2815_02113	-1.58	2.50E-03	sugar ABC transporter permease
malE	NBC2815_02115	-1.88	5.26E-05	sugar ABC transporter substrate-binding protein
NBC2815_02118	NBC2815_02118	1.51	2.23E-02	LacI family transcriptional regulator
scpA	NBC2815_02121	2.83	2.21E-11	segregation and condensation protein A
NBC2815_02122	NBC2815_02122	1.76	1.83E-04	transcriptional regulator containing the HTH domain protein
NBC2815_02123	NBC2815_02123	1.66	4.24E-03	pseudouridylate synthase
NBC2815_02126	NBC2815_02126	-2.61	7.21E-07	hydrolase
ybdL	NBC2815_02127	-2.58	4.25E-11	aminotransferase
ccmA	NBC2815_02128	-2.44	1.06E-06	cytochrome c biogenesis protein CcmA
NBC2815_02129	NBC2815_02129	-2.27	4.92E-04	heme exporter protein CcmB
ccmD2	NBC2815_02131	-1.93	2.95E-02	heme exporter protein D (c-type cytochrome biogenesis protein CcmD)
NBC2815_02132	NBC2815_02132	-2.37	5.86E-06	cytochrome c-type biogenesis protein CcmE
NBC2815_02133	NBC2815_02133	-1.78	1.97E-04	C-type cytochrome biogenesis membrane protein
NBC2815_02134	NBC2815_02134	-4.00	1.34E-18	C-type cytochrome biogenesis protein/thioredoxin
NBC2815_02135	NBC2815_02135	-3.39	3.00E-12	C-type cytochrome biogenesis protein
cycH	NBC2815_02136	-1.58	4.52E-02	C-type cytochrome biogenesis protein
NBC2815_02139	NBC2815_02139	-2.55	3.82E-02	IS1478 transposase
NBC2815_02144	NBC2815_02144	5.58	1.36E-14	methyl-accepting chemotaxis protein
NBC2815_02145	NBC2815_02145	2.38	9.60E-07	Hypothetical Protein
NBC2815_02146	NBC2815_02146	2.82	1.32E-07	Hypothetical Protein
NBC2815_02148	NBC2815_02148	1.67	5.92E-04	lipoprotein
surE	NBC2815_02150	-1.56	2.76E-03	stationary phase survival protein SurE
truD	NBC2815_02152	-1.66	1.05E-02	tRNA pseudouridine synthase D
ispF	NBC2815_02153	-1.94	1.17E-02	2-C-methyl-D-erythritol 2,4-cyclodiphosphate synthase
ispD	NBC2815_02154	-2.43	1.90E-10	2-C-methyl-D-erythritol 4-phosphate cytidylyltransferase

ftsB	NBC2815_02155	-4.02	1.70E-22	cell division protein FtsB
eno	NBC2815_02156	-3.44	3.36E-14	phosphopyruvate hydratase
NBC2815_02159	NBC2815_02159	4.83	3.91E-17	Hypothetical Protein
NBC2815_02160	NBC2815_02160	-3.33	0.00E+00	DNA topoisomerase IV subunit B /EC_number="5.99.1.-
NBC2815_02164	NBC2815_02164	-1.72	1.34E-04	pit accessory protein
NBC2815_02165	NBC2815_02165	1.63	5.09E-03	hemolysin
NBC2815_02166	NBC2815_02166	10.76	1.45E-27	exopolysaccharide synthesis protein exoD
NBC2815_02167	NBC2815_02167	13.21	1.93E-36	monooxygenase
NBC2815_02172	NBC2815_02172	1.52	1.93E-02	cytochrome C
NBC2815_02174	NBC2815_02174	2.17	6.25E-05	Hypothetical Protein
rpoE4	NBC2815_02175	4.17	6.07E-12	RNA polymerase ECF-type sigma factor
NBC2815_02176	NBC2815_02176	2.59	7.96E-06	Hypothetical Protein
NBC2815_02177	NBC2815_02177	4.10	3.23E-16	serine protease
NBC2815_02178	NBC2815_02178	2.92	2.85E-05	cytochrome C-type biogenesis protein
NBC2815_02181	NBC2815_02181	1.80	3.60E-02	C-type cytochrome biogenesis protein
NBC2815_02182	NBC2815_02182	2.12	8.09E-07	IS1389 transposase
NBC2815_02184	NBC2815_02184	3.72	6.50E-05	IS1404 transposase
NBC2815_02185	NBC2815_02185	2.05	8.98E-04	IS1480 transposase
NBC2815_02186	NBC2815_02186	1.94	2.42E-02	transposase
NBC2815_02188	NBC2815_02188	1.86	3.50E-02	tis1421-transposase b
thrC	NBC2815_02190	-2.11	2.30E-06	threonine synthase
NBC2815_02191	NBC2815_02191	1.91	1.48E-03	tannase
thrB	NBC2815_02193	2.23	9.61E-07	homoserine kinase
NBC2815_02196	NBC2815_02196	2.29	2.48E-02	IS1404 transposase protein B
NBC2815_02201	NBC2815_02201	-3.11	1.36E-03	integrase/recombinase
NBC2815_02203	NBC2815_02203	-1.81	6.25E-04	DNA helicase-like protein
NBC2815_02204	NBC2815_02204	-1.50	1.44E-02	hypothetical protein
NBC2815_02208	NBC2815_02208	-2.13	2.22E-06	piperideine-6-carboxylate dehydrogenase
NBC2815_02210	NBC2815_02210	-1.95	2.58E-05	putative methyltransferase
NBC2815_02211	NBC2815_02211	-2.12	1.32E-06	dihydroorotate dehydrogenase 2 /EC_number="1.3.98.1
murB	NBC2815_02212	-1.72	3.25E-04	UDP-N-acetylenolpyruvoylglucosamine reductase
ispG	NBC2815_02216	-1.78	2.14E-03	4-hydroxy-3-methylbut-2-en-1-yl diphosphate synthase
NBC2815_02218	NBC2815_02218	-2.02	1.47E-03	two-component system regulatory protein
NBC2815_02219	NBC2815_02219	2.21	2.58E-04	putative LysR family transcriptional regulator
NBC2815_02220	NBC2815_02220	4.73	1.49E-13	phosphodiesterase
NBC2815_02224	NBC2815_02224	-2.22	4.66E-03	transposase
NBC2815_02225	NBC2815_02225	-3.38	9.13E-05	Hypothetical Protein
NBC2815_02227	NBC2815_02227	-1.99	1.54E-03	Hypothetical Protein
sglT	NBC2815_02228	-4.65	1.35E-30	sodium/glucose cotransport protein
celD	NBC2815_02229	-3.38	2.11E-13	glucan 1,4-beta-glucosidase precursor
NBC2815_02231	NBC2815_02231	-1.92	6.05E-04	type IV secretion system protein VirD4
NBC2815_02232	NBC2815_02232	-6.90	1.35E-15	hypothetical protein
NBC2815_02233	NBC2815_02233	-5.26	6.96E-06	hypothetical protein
panD	NBC2815_02237	-2.98	9.00E-13	aspartate alpha-decarboxylase
NBC2815_02244	NBC2815_02244	-7.62	1.66E-15	GTP cyclohydrolase
xylE	NBC2815_02245	-5.51	3.00E-22	MFS transporter
NBC2815_02246	NBC2815_02246	-7.47	0.00E+00	xylose isomerase /EC_number="5.3.1.5
NBC2815_02247	NBC2815_02247	-5.26	5.20E-31	D-xylulokinase
NBC2815_02248	NBC2815_02248	-4.30	0.00E+00	Hypothetical Protein
NBC2815_02251	NBC2815_02251	2.97	8.21E-06	sialic acid-specific 9-O-acetylesterase
NBC2815_02252	NBC2815_02252	2.63	9.24E-11	TonB-dependent outer membrane receptor
NBC2815_02253	NBC2815_02253	1.61	2.09E-03	IS1478 transposase
NBC2815_02257	NBC2815_02257	2.17	3.52E-04	relaxation protein
dgoA	NBC2815_02259	8.53	1.41E-17	2-dehydro-3-deoxy-6-phosphogalactonate aldolase
NBC2815_02260	NBC2815_02260	3.88	1.71E-12	galactonate dehydratase
NBC2815_02261	NBC2815_02261	1.99	6.17E-04	regucalcin
dgoK	NBC2815_02262	3.06	5.11E-09	2-oxo-3-deoxygalactonate kinase
NBC2815_02265	NBC2815_02265	2.09	1.22E-05	dihydrodipicolinate synthase /EC_number="4.3.3.7
gcvR	NBC2815_02266	-4.15	3.80E-20	glycine cleavage system transcriptional repressor
NBC2815_02267	NBC2815_02267	-3.60	1.55E-17	Hypothetical Protein
NBC2815_02268	NBC2815_02268	-2.29	4.13E-09	bacterioferritin comigratory protein
NBC2815_02269	NBC2815_02269	-6.44	1.04E-37	PhoH-like protein
NBC2815_02270	NBC2815_02270	-7.18	7.40E-29	Hypothetical Protein
NBC2815_02271	NBC2815_02271	-1.67	1.21E-02	phosphomethylpyrimidine kinase /EC_number="2.7.4.7
NBC2815_02276	NBC2815_02276	-2.05	9.07E-04	transcriptional regulator
NBC2815_02280	NBC2815_02280	3.46	2.62E-11	carbon storage regulator
tldD	NBC2815_02287	4.54	1.14E-07	modulator of DNA gyrase
NBC2815_02289	NBC2815_02289	1.78	2.37E-03	Pectate lyase precursor /EC_number="4.2.2.2
NBC2815_02290	NBC2815_02290	2.95	7.40E-06	secreted protein
NBC2815_02291	NBC2815_02291	1.98	1.07E-03	cointegrate resolution protein T (fragment)
NBC2815_02293	NBC2815_02293	-1.64	1.72E-02	IS1478 transposase
NBC2815_02298	NBC2815_02298	2.49	6.87E-04	hypothetical protein
NBC2815_02299	NBC2815_02299	3.04	7.02E-08	cointegrate resolution protein T (fragment)
tnpA_2	NBC2815_02303	1.70	7.87E-03	Tn5044 transposase
tnpA_3	NBC2815_02304	2.88	7.05E-12	Tn5044 transposase
NBC2815_02308	NBC2815_02308	-2.08	2.85E-04	competence-damaged protein
NBC2815_02309	NBC2815_02309	-2.17	2.07E-06	GTP-binding protein
hfq	NBC2815_02310	-4.76	6.03E-21	RNA-binding protein Hfq
hflB	NBC2815_02313	2.44	7.08E-06	cell division protein
ftsJ	NBC2815_02314	2.32	4.89E-06	cell division protein
NBC2815_02317	NBC2815_02317	1.56	1.62E-02	transposase
NBC2815_02321	NBC2815_02321	1.82	2.34E-03	IS1404 transposase protein B
NBC2815_02326	NBC2815_02326	2.77	2.68E-02	IS1478 transposase
NBC2815_02332	NBC2815_02332	3.36	2.90E-06	hypothetical protein
NBC2815_02333	NBC2815_02333	1.90	6.43E-03	VGR-like protein
NBC2815_02336	NBC2815_02336	1.89	8.34E-03	hypothetical protein
NBC2815_02337	NBC2815_02337	3.14	1.34E-05	Rhs element Vgr protein
NBC2815_02341	NBC2815_02341	3.24	1.27E-06	hypothetical protein
NBC2815_02342	NBC2815_02342	7.08	3.15E-10	VGR-like protein
NBC2815_02344	NBC2815_02344	-2.70	2.35E-04	hypothetical protein
NBC2815_02345	NBC2815_02345	1.85	2.91E-02	hypothetical protein
NBC2815_02346	NBC2815_02346	2.42	3.87E-06	Rhs element Vgr protein
NBC2815_02347	NBC2815_02347	-3.24	1.16E-13	sulfatase modifying factor 1 (C-alpha-formyglycine-generating enzyme 1)
NBC2815_02349	NBC2815_02349	11.84	3.97E-32	Rhs element Vgr protein

NBC2815_02351	NBC2815_02351	-2.70	6.12E-06	hypothetical protein
NBC2815_02352	NBC2815_02352	2.10	9.63E-03	hypothetical protein
NBC2815_02353	NBC2815_02353	4.53	1.23E-06	VGR-related protein
NBC2815_02355	NBC2815_02355	-3.08	1.42E-10	lipoprotein
NBC2815_02357	NBC2815_02357	1.79	2.29E-03	Rhs element Vgr protein
NBC2815_02358	NBC2815_02358	1.64	2.78E-03	membrane protein
NBC2815_02360	NBC2815_02360	-1.85	7.44E-05	OmpA family domain-containing protein
NBC2815_02361	NBC2815_02361	-1.57	3.64E-03	hypothetical protein
NBC2815_02363	NBC2815_02363	1.72	3.28E-03	transmembrane protein
NBC2815_02364	NBC2815_02364	-1.86	1.26E-05	paar motif family protein
NBC2815_02365	NBC2815_02365	1.76	9.58E-04	transmembrane protein
NBC2815_02367	NBC2815_02367	2.17	9.80E-06	RhsD protein
NBC2815_02368	NBC2815_02368	-4.27	3.26E-07	hypothetical protein
NBC2815_02369	NBC2815_02369	-1.60	9.70E-03	RhsD protein
NBC2815_02370	NBC2815_02370	-5.99	2.56E-29	hypothetical protein
NBC2815_02371	NBC2815_02371	-4.93	1.19E-23	hypothetical protein
NBC2815_02374	NBC2815_02374	2.53	7.40E-08	hypothetical protein
NBC2815_02375	NBC2815_02375	2.13	4.60E-05	Rhs element Vgr protein
NBC2815_02380	NBC2815_02380	17.92	6.26E-06	hypothetical protein
xerC_1	NBC2815_02381	1.87	2.52E-02	Integrase
NBC2815_02382	NBC2815_02382	1.52	6.43E-03	phage-related integrase
NBC2815_02383	NBC2815_02383	2.44	2.86E-09	IS1480 transposase
NBC2815_02393	NBC2815_02393	-1.58	2.08E-03	ABC transporter ATP-binding protein
NBC2815_02395	NBC2815_02395	-1.84	1.32E-05	biopolymer transport protein
NBC2815_02397	NBC2815_02397	1.73	5.92E-04	aldo-keto reductase family protein
lolC	NBC2815_02399	-1.72	8.10E-04	ABC transporter-type lipoprotein-releasing protein
NBC2815_02411	NBC2815_02411	-1.56	3.18E-03	phosphogluconate dehydratase /EC_number="4.2.1.12
NBC2815_02412	NBC2815_02412	-1.79	3.05E-05	keto-hydroxyglutarate-aldolase/keto-deoxy- phosphogluconate aldolase
NBC2815_02421	NBC2815_02421	1.63	5.68E-03	two-component system sensor protein
tex	NBC2815_02422	2.68	5.00E-08	transcription-like protein
NBC2815_02423	NBC2815_02423	4.14	4.21E-16	Hypothetical Protein
NBC2815_02424	NBC2815_02424	27.27	5.04E-06	FAD dependent oxidoreductase
NBC2815_02425	NBC2815_02425	6.42	2.18E-05	FAD dependent oxidoreductase
NBC2815_02426	NBC2815_02426	3.24	1.10E-08	Hypothetical Protein
NBC2815_02427	NBC2815_02427	2.40	1.02E-05	Hypothetical Protein
pssA_2	NBC2815_02430	1.55	2.43E-02	CDP-diacylglycerol-serine o-phosphatidyltransferase
NBC2815_02432	NBC2815_02432	1.54	1.50E-02	Hypothetical Protein
NBC2815_02433	NBC2815_02433	1.81	7.04E-04	GTP-binding protein
NBC2815_02435	NBC2815_02435	1.58	1.24E-03	small conductance mechanosensitive ion channel
NBC2815_02436	NBC2815_02436	-1.87	2.75E-05	oligoribonuclease /EC_number="3.1.-.-
tadA	NBC2815_02437	1.59	1.13E-02	putative tRNA-specific adenosine deaminase
NBC2815_02438	NBC2815_02438	-1.62	2.86E-02	manganese transport regulator MntR
smf2	NBC2815_02439	2.92	8.16E-06	manganese transport protein MntH
NBC2815_02443	NBC2815_02443	2.31	1.68E-05	Hypothetical Protein
NBC2815_02455	NBC2815_02455	-2.89	1.17E-11	DNA helicase-like protein
proA	NBC2815_02459	-2.96	1.56E-15	gamma-glutamyl phosphate reductase
NBC2815_02462	NBC2815_02462	-2.88	1.55E-03	cupin
argH	NBC2815_02463	-2.12	2.92E-06	argininosuccinate lyase
NBC2815_02470	NBC2815_02470	1.60	3.13E-03	Hypothetical Protein
cysS	NBC2815_02471	-1.58	2.78E-03	cysteinyl-tRNA synthetase
NBC2815_02472	NBC2815_02472	-3.21	1.26E-11	SufE protein involved in Fe-S center assembly
NBC2815_02473	NBC2815_02473	2.90	3.58E-10	drug:proton antiporter
dksA	NBC2815_02474	-1.54	1.38E-02	DnaK supressor
NBC2815_02475	NBC2815_02475	-1.54	6.06E-03	dihydroorotase /EC_number="3.5.2.3
NBC2815_02477	NBC2815_02477	-1.87	2.08E-02	ISXo1 transposase, IS5 family
NBC2815_02478	NBC2815_02478	1.93	4.97E-05	general stress protein
NBC2815_02480	NBC2815_02480	1.85	2.05E-04	Hypothetical Protein
NBC2815_02481	NBC2815_02481	2.00	2.98E-03	pectate lyase
NBC2815_02482	NBC2815_02482	2.94	2.00E-08	polygalacturonase
NBC2815_02483	NBC2815_02483	-2.18	1.21E-03	Phytoene/squalene synthetase
cbbZ	NBC2815_02484	-1.80	4.07E-03	phosphoglycolate phosphatase
NBC2815_02485	NBC2815_02485	-1.98	1.15E-06	bifunctional 3-demethylubiquinone-9 3-methyltransferase/ 2-octaprenyl-6-hydroxy phenol methylase
NBC2815_02486	NBC2815_02486	-1.57	9.87E-04	N-ethylammeline chlorohydrolase
NBC2815_02487	NBC2815_02487	-1.85	2.74E-05	Hypothetical Protein
efP	NBC2815_02488	-3.55	3.58E-16	elongation factor P
NBC2815_02490	NBC2815_02490	-2.24	3.09E-06	tRNA/rRNA methyltransferase
NBC2815_02492	NBC2815_02492	-2.85	4.75E-08	superoxide dismutase
NBC2815_02493	NBC2815_02493	2.52	1.72E-06	ribonuclease
NBC2815_02494	NBC2815_02494	1.92	3.78E-04	Hypothetical Protein
NBC2815_02495	NBC2815_02495	-1.64	1.22E-03	ABC transporter ATP-binding protein
NBC2815_02498	NBC2815_02498	3.24	3.62E-16	Hypothetical Protein
NBC2815_02506	NBC2815_02506	4.61	3.72E-12	heat shock protein HtpX
phbB	NBC2815_02508	-1.88	1.64E-04	acetoacetyl-CoA reductase
phaR	NBC2815_02509	-3.90	8.49E-23	polyhydroxyalkanoate synthesis repressor PhaR
NBC2815_02510	NBC2815_02510	-2.81	1.28E-12	GumN protein
NBC2815_02511	NBC2815_02511	-2.69	5.26E-11	lipoprotein
mutL	NBC2815_02512	-1.77	4.46E-05	DNA mismatch repair protein
NBC2815_02514	NBC2815_02514	-1.91	6.40E-05	ATPase or kinase
mmuP	NBC2815_02524	2.07	1.11E-05	putative S-methylmethionine transporter
NBC2815_02525	NBC2815_02525	-2.51	2.82E-10	hypothetical protein
metG	NBC2815_02526	-1.93	7.35E-06	methionyl-tRNA synthetase
serB	NBC2815_02527	-2.74	1.21E-11	Phosphoserine phosphatase
NBC2815_02530	NBC2815_02530	1.52	8.81E-03	Hypothetical Protein
NBC2815_02536	NBC2815_02536	-1.86	2.01E-05	cyclopropane-fatty-acyl-phospholipid synthase
NBC2815_02541	NBC2815_02541	-4.13	2.18E-19	endoribonuclease L-PSP
NBC2815_02544	NBC2815_02544	2.71	1.33E-03	hypothetical protein
NBC2815_02547	NBC2815_02547	1.72	2.82E-02	Hypothetical Protein
NBC2815_02548	NBC2815_02548	1.97	3.89E-05	Hypothetical Protein
NBC2815_02549	NBC2815_02549	11.92	4.56E-32	arabinose efflux porter
nerA	NBC2815_02550	6.56	8.87E-19	GTN reductase
NBC2815_02551	NBC2815_02551	6.42	1.55E-21	alkylphosphonate utilization operon protein PhnA
NBC2815_02552	NBC2815_02552	3.97	1.39E-14	cation diffusion facilitator family protein
NBC2815_02554	NBC2815_02554	2.08	1.21E-05	Hypothetical Protein
xopD_1	NBC2815_02558	1.52	1.20E-02	outer protein D

NBC2815_02560	NBC2815_02560	1.65	1.49E-03	diadenosine tetraphosphate hydrolase
NBC2815_02561	NBC2815_02561	-3.01	2.97E-08	Hypothetical Protein
NBC2815_02562	NBC2815_02562	-1.76	6.19E-04	Hypothetical Protein
dcd	NBC2815_02563	-2.03	9.23E-07	deoxycytidine triphosphate deaminase
NBC2815_02567	NBC2815_02567	-2.77	3.70E-09	metallopeptidase
NBC2815_02568	NBC2815_02568	-3.71	0.00E+00	metallopeptidase
NBC2815_02573	NBC2815_02573	-1.61	3.34E-03	peptidyl-prolyl cis-trans isomerase
NBC2815_02575	NBC2815_02575	-1.79	2.77E-03	outer membrane lipoprotein
NBC2815_02581	NBC2815_02581	-2.22	7.74E-04	IS1478 transposase
NBC2815_02585	NBC2815_02585	-2.76	2.21E-03	IS5 transposase
NBC2815_02586	NBC2815_02586	1.55	2.82E-02	transposase, fragment
NBC2815_02589	NBC2815_02589	2.10	3.12E-06	phage-related integrase
NBC2815_02591	NBC2815_02591	5.47	1.86E-18	transcriptional regulator
NBC2815_02592	NBC2815_02592	-1.62	1.20E-03	seryl-tRNA ligase /EC_number="6.1.1.11
NBC2815_02593	NBC2815_02593	1.90	9.43E-06	TonB-like protein
NBC2815_02594	NBC2815_02594	6.97	9.58E-26	TonB-like protein
NBC2815_02595	NBC2815_02595	1.93	1.47E-04	3-phosphoshikimate 1-carboxyvinyltransferase /EC_number="2.5.1.19
NBC2815_02596	NBC2815_02596	-1.58	4.25E-03	P-protein
NBC2815_02598	NBC2815_02598	1.71	4.23E-04	sulfite oxidase subunit YedY
rpfN	NBC2815_02604	-2.91	7.26E-08	regulator of pathogenicity factors
secF	NBC2815_02605	-1.79	6.22E-04	preprotein translocase subunit SecF
NBC2815_02610	NBC2815_02610	2.66	4.99E-09	AsnC family transcriptional regulator
NBC2815_02612	NBC2815_02612	2.43	2.86E-07	Hypothetical Protein
NBC2815_02617	NBC2815_02617	-2.39	3.32E-09	gamma-glutamyltranspeptidase
NBC2815_02618	NBC2815_02618	-4.29	1.87E-25	ferredoxin
NBC2815_02619	NBC2815_02619	-4.06	0.00E+00	Hypothetical Protein
coaD	NBC2815_02620	-1.83	1.61E-04	phosphopantetheine adenylyltransferase
NBC2815_02622	NBC2815_02622	-1.58	2.54E-02	heat shock protein 90
NBC2815_02623	NBC2815_02623	1.87	4.16E-03	IS1595 transposase
phaZ	NBC2815_02624	1.71	2.86E-03	Poly-beta-hydroxyalkanoate depolymerase
NBC2815_02626	NBC2815_02626	2.17	1.48E-04	Hypothetical Protein
NBC2815_02627	NBC2815_02627	-2.00	1.25E-02	ISXo1 transposase, IS5 family
NBC2815_02628	NBC2815_02628	1.74	4.20E-02	integrase, catalytic region
NBC2815_02629	NBC2815_02629	1.63	2.82E-02	small domain family methyltransferase
phoA	NBC2815_02631	-1.80	1.10E-05	alkaline phosphatase precursor
mesJ	NBC2815_02632	-2.03	3.76E-03	cell cycle protein
NBC2815_02633	NBC2815_02633	-1.89	7.43E-04	exodeoxyribonuclease VII small subunit /EC_number="3.1.11.6
NBC2815_02634	NBC2815_02634	-1.62	1.20E-03	geranyltranstransferase
NBC2815_02635	NBC2815_02635	1.83	2.16E-04	extracellular protease
NBC2815_02636	NBC2815_02636	1.69	1.17E-03	Hypothetical Protein
NBC2815_02637	NBC2815_02637	3.34	2.83E-11	modulator of DNA gyrase; peptidase U62 protein PmbA
NBC2815_02638	NBC2815_02638	1.75	7.67E-04	Hypothetical Protein
NBC2815_02639	NBC2815_02639	3.19	1.20E-08	TldD protein
NBC2815_02650	NBC2815_02650	-2.40	2.53E-08	Hypothetical Protein
NBC2815_02657	NBC2815_02657	5.50	2.39E-11	Hypothetical Protein
hpa2	NBC2815_02658	3.71	7.05E-14	type III secretion-system related transglycosylase
hpa1	NBC2815_02659	5.23	3.23E-07	Hpa1 protein
NBC2815_02660	NBC2815_02660	1.86	3.19E-04	HrcC protein
NBC2815_02661	NBC2815_02661	3.36	6.12E-12	HrpB8 protein
NBC2815_02662	NBC2815_02662	2.51	1.20E-07	HrpB7 protein
NBC2815_02663	NBC2815_02663	3.58	2.03E-11	type III secretion system ATPase
NBC2815_02664	NBC2815_02664	3.97	3.16E-13	type III secretion system protein HrpB
NBC2815_02665	NBC2815_02665	4.43	2.56E-14	HrpB4 protein
NBC2815_02666	NBC2815_02666	5.25	1.76E-14	HrcJ protein
hrpB2	NBC2815_02667	3.38	3.62E-07	protein HrpB2
NBC2815_02668	NBC2815_02668	3.30	2.14E-06	HrpB1 protein
NBC2815_02669	NBC2815_02669	6.25	1.18E-20	type III secretion system protein HrcU
NBC2815_02670	NBC2815_02670	3.59	2.27E-09	HrcV protein
hpaP	NBC2815_02671	4.19	3.92E-13	type III secretion control protein HpaP
NBC2815_02672	NBC2815_02672	3.75	3.61E-13	HrcQ protein
hrcR	NBC2815_02673	2.41	3.22E-07	type III secretion system protein
NBC2815_02674	NBC2815_02674	3.03	6.66E-10	HrcS protein
NBC2815_02675	NBC2815_02675	2.86	5.50E-08	HpaA protein
NBC2815_02676	NBC2815_02676	3.29	1.13E-07	HrpD5 protein
NBC2815_02678	NBC2815_02678	1.79	4.53E-02	hypothetical protein
hpaB	NBC2815_02679	2.72	8.91E-06	HpaB protein, type III secretion system
hrpW	NBC2815_02680	2.52	3.90E-05	HrpW protein
NBC2815_02681	NBC2815_02681	2.63	5.39E-08	Hypothetical Protein
hpa3_1	NBC2815_02683	2.42	1.27E-07	Hpa3 protein, type III secretion system
NBC2815_02684	NBC2815_02684	-2.84	2.00E-08	Hypothetical Protein
NBC2815_02689	NBC2815_02689	-1.69	1.12E-03	lipoprotein
NBC2815_02691	NBC2815_02691	2.21	1.71E-06	tis1421-transposase a
NBC2815_02692	NBC2815_02692	2.57	2.43E-02	transposase
NBC2815_02693	NBC2815_02693	1.87	3.83E-05	ABC transporter ATP-binding protein
yjl094C	NBC2815_02695	-1.66	1.37E-03	cation:proton antiporter
NBC2815_02699	NBC2815_02699	-3.06	3.78E-02	GAF domain-containing protein
NBC2815_02702	NBC2815_02702	2.31	1.10E-04	putative signal protein with PAS(PAC), GGDEF and EAL domains
rpoE5	NBC2815_02704	15.95	1.14E-03	RNA polymerase ECF-type sigma factor
NBC2815_02705	NBC2815_02705	-1.92	4.62E-04	Hypothetical Protein
NBC2815_02708	NBC2815_02708	1.52	5.66E-03	Hypothetical Protein
NBC2815_02709	NBC2815_02709	2.10	4.96E-04	Hypothetical Protein
NBC2815_02712	NBC2815_02712	-1.67	8.23E-04	phosphodiesterase-nucleotide pyrophosphatase
NBC2815_02713	NBC2815_02713	-4.53	1.72E-18	Hypothetical Protein
phuR_1	NBC2815_02715	-2.36	2.17E-06	Outer membrane hemin receptor
fhuA_1	NBC2815_02718	4.00	5.12E-08	TonB-dependent receptor
fhuA_2	NBC2815_02719	4.98	7.79E-10	TonB-dependent receptor
NBC2815_02720	NBC2815_02720	3.74	8.69E-08	extracellular serine protease
NBC2815_02721	NBC2815_02721	5.33	8.57E-15	lipoprotein
NBC2815_02722	NBC2815_02722	3.92	1.07E-18	serine protease
NBC2815_02723	NBC2815_02723	2.22	1.35E-06	serine protease
NBC2815_02724	NBC2815_02724	2.53	9.39E-06	Hypothetical Protein
NBC2815_02725	NBC2815_02725	2.15	5.05E-07	Hypothetical Protein
NBC2815_02726	NBC2815_02726	15.10	3.83E-13	cellulase
NBC2815_02728	NBC2815_02728	3.39	1.29E-12	FUR family transcriptional regulator

NBC2815_02732	NBC2815_02732	7.19	6.93E-41	cysteine protease
NBC2815_02734	NBC2815_02734	2.22	1.35E-06	N-acetyltransferase family protein
mutS	NBC2815_02736	-1.53	3.97E-03	DNA mismatch repair protein MutS
NBC2815_02737	NBC2815_02737	8.41	1.40E-23	catalase/hydroperoxidase HPI(I)
NBC2815_02739	NBC2815_02739	1.65	1.00E-02	tis1421-transposase b
NBC2815_02740	NBC2815_02740	2.27	2.46E-08	IS1404 transposase
NBC2815_02743	NBC2815_02743	-1.86	3.71E-02	Hypothetical Protein
gpmA	NBC2815_02745	-1.75	4.29E-04	phosphoglycerate mutase
NBC2815_02746	NBC2815_02746	4.74	1.88E-07	hypothetical protein
NBC2815_02748	NBC2815_02748	-5.11	8.84E-10	hypothetical protein
NBC2815_02750	NBC2815_02750	4.11	5.50E-17	tis1421-transposase b
NBC2815_02751	NBC2815_02751	11.12	1.83E-27	pirin
cstA_1	NBC2815_02753	2.17	7.18E-06	carbon starvation protein A
NBC2815_02760	NBC2815_02760	6.43	1.74E-17	Hypothetical Protein
NBC2815_02761	NBC2815_02761	4.41	8.72E-06	hypothetical protein
NBC2815_02762	NBC2815_02762	4.35	1.99E-19	putative xanthine dehydrogenase accessory factor XdhC
NBC2815_02763	NBC2815_02763	3.63	1.39E-14	oxidoreductase
NBC2815_02764	NBC2815_02764	1.66	1.07E-03	Response regulator
ssb	NBC2815_02765	-2.52	2.13E-10	single-stranded DNA-binding protein
NBC2815_02767	NBC2815_02767	1.89	7.54E-04	dienelactone hydrolase family protein
murD	NBC2815_02768	-1.88	1.87E-05	UDP-N-acetylmuramoyl-L-alanyl-D-glutamate synthetase
NBC2815_02769	NBC2815_02769	-1.75	3.41E-04	Hypothetical Protein
NBC2815_02771	NBC2815_02771	-1.76	4.98E-04	bifunctional aspartate kinase/diaminopimelate decarboxylase
NBC2815_02774	NBC2815_02774	3.73	1.24E-10	rickettsia 17 kDa surface antigen family protein
pyrB	NBC2815_02776	-1.91	4.40E-06	aspartate carbamoyltransferase catalytic subunit
NBC2815_02779	NBC2815_02779	2.00	1.09E-02	DNA-3-methyladenine glycosylase I
pilU	NBC2815_02782	-1.64	3.55E-04	twitching motility protein
pilT	NBC2815_02783	-1.93	6.55E-05	type IV pilus assembly protein PilT
NBC2815_02784	NBC2815_02784	-1.52	1.06E-02	TIM-barrel fold containing protein
NBC2815_02786	NBC2815_02786	-4.75	0.00E+00	histone-like protein
NBC2815_02788	NBC2815_02788	5.89	3.69E-23	Hypothetical Protein
NBC2815_02789	NBC2815_02789	2.34	8.33E-07	Hypothetical Protein
NBC2815_02790	NBC2815_02790	-2.96	4.49E-13	nuclear protein SET
NBC2815_02791	NBC2815_02791	5.13	5.75E-17	transcriptional regulator
NBC2815_02792	NBC2815_02792	4.31	4.10E-14	cysteine desulfurase activator complex subunit SufB
NBC2815_02793	NBC2815_02793	3.51	6.62E-10	ABC transporter ATP-binding protein
NBC2815_02794	NBC2815_02794	4.89	6.59E-14	ABC transporter permease
NBC2815_02795	NBC2815_02795	3.38	8.25E-09	cysteine desulfurase
fhuA_3	NBC2815_02799	5.34	1.17E-20	TonB-dependent receptor
NBC2815_02800	NBC2815_02800	7.05	3.51E-13	Fe(II)-dependent oxygenase superfamily protein
NBC2815_02801	NBC2815_02801	4.84	3.73E-13	TPR repeat protein
NBC2815_02805	NBC2815_02805	-1.93	9.76E-03	Hypothetical Protein
NBC2815_02806	NBC2815_02806	-19.21	0.00E+00	ComEA-related DNA uptake protein
NBC2815_02809	NBC2815_02809	-1.68	1.40E-03	phosphotransferase
NBC2815_02811	NBC2815_02811	1.59	2.05E-03	replication related protein
NBC2815_02814	NBC2815_02814	-2.00	1.11E-06	phosphoribosylaminoimidazole synthetase
NBC2815_02816	NBC2815_02816	1.53	2.37E-02	Hypothetical Protein
NBC2815_02820	NBC2815_02820	-1.81	1.95E-03	Hypothetical Protein
murA	NBC2815_02821	-1.90	2.61E-06	UDP-N-acetylglucosamine 1-carboxyvinyltransferase
NBC2815_02822	NBC2815_02822	-2.41	6.54E-05	transcription regulator BolA
NBC2815_02823	NBC2815_02823	1.61	3.02E-03	polysialic acid capsule expression protein
NBC2815_02824	NBC2815_02824	1.82	2.69E-05	phosphatase, YrbI family
NBC2815_02831	NBC2815_02831	-1.54	2.85E-03	hpr kinase/phosphorylase
NBC2815_02840	NBC2815_02840	-3.62	4.16E-19	hypothetical protein
NBC2815_02841	NBC2815_02841	-1.76	1.05E-03	Hypothetical Protein
NBC2815_02842	NBC2815_02842	-2.33	1.27E-07	type IV secretion system protein VirD4
glk_2	NBC2815_02843	1.58	4.57E-03	glucokinase
iroN_1	NBC2815_02845	-1.69	3.28E-03	TonB-dependent receptor
NBC2815_02847	NBC2815_02847	-2.53	2.46E-08	glycosyl hydrolase
NBC2815_02854	NBC2815_02854	-7.44	0.00E+00	putative secreted protein
NBC2815_02858	NBC2815_02858	-2.88	2.52E-11	secreted protein
NBC2815_02863	NBC2815_02863	-2.37	1.87E-07	aminopeptidase
purA	NBC2815_02864	-1.66	6.26E-04	Adenylosuccinate synthase
dnaJ2	NBC2815_02869	1.69	1.77E-02	chaperone protein
NBC2815_02871	NBC2815_02871	2.06	5.77E-06	peroxiredoxin
prpF	NBC2815_02877	-2.02	2.65E-06	AcnD-accessory protein PrpF
prpR	NBC2815_02881	-1.55	9.93E-03	propionate catabolism regulatory protein
NBC2815_02885	NBC2815_02885	1.70	2.14E-02	DNA helicase-like protein
NBC2815_02886	NBC2815_02886	-2.83	1.85E-03	hypothetical protein
NBC2815_02888	NBC2815_02888	-2.31	1.14E-04	Hypothetical Protein
NBC2815_02894	NBC2815_02894	-1.52	1.07E-02	Hypothetical Protein
NBC2815_02898	NBC2815_02898	-2.44	6.41E-09	esterase EstE
thiG	NBC2815_02900	-2.74	4.18E-12	thiazole synthase
trmB	NBC2815_02901	-3.01	2.62E-12	tRNA (guanine-N(7)-)-methyltransferase
NBC2815_02906	NBC2815_02906	3.47	4.88E-08	aminopeptidase
dsbB	NBC2815_02911	-3.63	2.97E-12	disulfide bond formation protein B
rplQ	NBC2815_02912	-2.47	7.04E-07	50S ribosomal protein L17
rpsD	NBC2815_02914	-1.91	1.73E-04	30S ribosomal protein S4
rpsK	NBC2815_02915	-2.42	3.26E-07	30S ribosomal protein S11
rpsM	NBC2815_02916	-2.26	1.37E-06	30S ribosomal protein S13
rplO	NBC2815_02918	-2.24	2.52E-06	50S ribosomal protein L15
rpmD	NBC2815_02919	-2.44	6.90E-07	50S ribosomal protein L30
rpsE	NBC2815_02920	-3.25	4.20E-09	30S ribosomal protein S5
rplF	NBC2815_02922	-1.51	1.94E-02	50S ribosomal protein L6
rpsH	NBC2815_02923	-1.67	3.79E-03	30S ribosomal protein S8
rpsQ	NBC2815_02928	-1.74	2.35E-03	30S ribosomal protein S17
rpmC	NBC2815_02929	-1.72	2.44E-03	50S ribosomal protein L29
rplP	NBC2815_02930	-1.54	2.20E-02	50S ribosomal protein L16
rplW	NBC2815_02935	-3.08	3.67E-06	50S ribosomal protein L23
rplD	NBC2815_02936	-1.68	2.30E-02	50S ribosomal protein L4
NBC2815_02940	NBC2815_02940	-2.05	4.12E-05	elongation factor G
rpsG	NBC2815_02941	-2.16	4.45E-06	30S ribosomal protein S7
rpsL	NBC2815_02942	-2.31	1.49E-06	30S ribosomal protein S12
rpoC	NBC2815_02943	-2.31	1.08E-06	DNA-directed RNA polymerase, subunit Beta-prime protein

rpoB	NBC2815_02944	-2.20	3.52E-06	DNA-directed RNA polymerase subunit beta
rplL	NBC2815_02945	-1.99	1.82E-04	50S ribosomal protein L7/L12
rplK	NBC2815_02948	-1.77	1.16E-03	50S ribosomal protein L11
nusG	NBC2815_02949	-1.55	7.85E-03	transcription antitermination protein NusG
NBC2815_02958	NBC2815_02958	-1.79	1.83E-03	50S ribosomal protein L25/general stress protein Ctc
NBC2815_02959	NBC2815_02959	1.50	1.90E-02	ribose-phosphate pyrophosphokinase /EC_number="2.7.6.1
NBC2815_02968	NBC2815_02968	2.29	3.53E-08	transcriptional regulator
NBC2815_02969	NBC2815_02969	7.75	1.54E-16	Hypothetical Protein
NBC2815_02975	NBC2815_02975	2.08	7.88E-03	transcriptional regulator
NBC2815_02976	NBC2815_02976	9.00	7.63E-13	extracellular protease
NBC2815_02977	NBC2815_02977	5.23	6.84E-05	extracellular protease
NBC2815_02982	NBC2815_02982	-1.68	1.17E-02	Hypothetical Protein
rpoE1	NBC2815_02985	2.86	1.26E-10	RNA polymerase factor sigma-70
NBC2815_02986	NBC2815_02986	1.96	2.79E-02	Hypothetical Protein
NBC2815_02987	NBC2815_02987	2.56	1.16E-04	Hypothetical Protein
NBC2815_02993	NBC2815_02993	-2.90	1.49E-06	hypothetical protein
hemK	NBC2815_02995	1.75	6.17E-03	protoporphyrinogen oxidase
NBC2815_02996	NBC2815_02996	13.19	1.37E-51	alkyl hydroperoxide reductase subunit C
ahpF	NBC2815_02997	45.66	1.33E-85	alkyl hydroperoxide reductase
NBC2815_02998	NBC2815_02998	16.29	1.88E-60	oxidative stress transcriptional regulator
greA_2	NBC2815_02999	5.94	2.75E-15	Transcription elongation factor
NBC2815_03000	NBC2815_03000	3.76	4.70E-10	transaldolase B /EC_number="2.2.1.2
pms	NBC2815_03001	5.22	5.54E-15	methionine sulfoxide reductase A
NBC2815_03002	NBC2815_03002	7.24	1.01E-05	hypothetical protein
NBC2815_03003	NBC2815_03003	2.44	7.28E-04	two-component system regulatory protein
NBC2815_03004	NBC2815_03004	2.33	6.20E-04	two-component system sensor protein
NBC2815_03005	NBC2815_03005	2.61	1.89E-02	Hypothetical Protein
NBC2815_03007	NBC2815_03007	-2.56	1.82E-12	glutaminyl-tRNA synthetase /EC_number="6.1.1.18
NBC2815_03008	NBC2815_03008	-1.56	2.75E-02	putative secreted protein
NBC2815_03013	NBC2815_03013	1.64	1.50E-03	Hypothetical Protein
NBC2815_03014	NBC2815_03014	2.15	3.63E-02	p-hydroxycinnamoyl CoA hydratase/lyase
pcaQ	NBC2815_03015	2.24	4.61E-05	transcriptional regulator
NBC2815_03016	NBC2815_03016	3.32	4.36E-03	protocatechuate 4,5-dioxygenase subunit alpha
ostA	NBC2815_03024	1.77	1.40E-03	organic solvent tolerance protein
pdxA	NBC2815_03026	1.64	6.38E-03	4-hydroxythreonine-4-phosphate dehydrogenase
apaG	NBC2815_03028	2.30	2.76E-08	CO2+/MG2+ efflux protein ApaG
apaH	NBC2815_03029	1.77	7.17E-04	diadenosine tetraphosphatase
NBC2815_03031	NBC2815_03031	-2.81	3.72E-10	Hypothetical Protein
thyA	NBC2815_03032	-2.17	3.26E-07	thymidylate synthase
NBC2815_03033	NBC2815_03033	-1.79	3.53E-04	prolipoprotein diacylglyceryl transferase /EC_number="2.4.99.-
rbsK	NBC2815_03036	-1.51	3.65E-03	ribokinase
yeiM	NBC2815_03037	-1.63	8.00E-03	nucleoside transporter
NBC2815_03038	NBC2815_03038	-1.52	2.99E-02	voltage-gated potassium channel subunit beta
NBC2815_03039	NBC2815_03039	8.18	5.78E-17	Hypothetical Protein
phuR_2	NBC2815_03040	6.68	1.05E-33	outer membrane hemin receptor
NBC2815_03046	NBC2815_03046	3.68	2.08E-04	hypothetical protein
NBC2815_03047	NBC2815_03047	2.00	6.28E-04	taurine dioxygenase
NBC2815_03048	NBC2815_03048	2.51	2.33E-03	hypothetical protein
NBC2815_03049	NBC2815_03049	3.19	3.25E-12	oxidoreductase
ftsE_2	NBC2815_03050	2.49	2.86E-08	ABC transporter ATP-binding protein
NBC2815_03051	NBC2815_03051	2.10	1.17E-06	acyl-CoA thioesterase
NBC2815_03054	NBC2815_03054	2.45	1.99E-02	Lysophospholipase L1
NBC2815_03056	NBC2815_03056	-2.11	2.98E-02	diacylglycerol kinase
lemA	NBC2815_03057	1.64	3.36E-03	Hypothetical Protein
NBC2815_03058	NBC2815_03058	2.04	1.17E-05	TIM-barrel protein; NifR3 family protein
NBC2815_03060	NBC2815_03060	2.00	4.11E-06	membrane-bound metal-dependent hydrolase
metK	NBC2815_03061	-2.55	2.46E-08	methionine adenosyltransferase
NBC2815_03067	NBC2815_03067	3.61	1.68E-03	peptide hydrolase
NBC2815_03069	NBC2815_03069	-1.70	3.74E-02	sulfotransferase
NBC2815_03070	NBC2815_03070	-1.61	3.45E-02	hypothetical protein
NBC2815_03072	NBC2815_03072	1.79	7.70E-03	hypothetical protein
NBC2815_03073	NBC2815_03073	-2.07	3.37E-06	Hypothetical Protein
NBC2815_03075	NBC2815_03075	3.72	3.80E-11	alpha-amylase (fragment)
NBC2815_03076	NBC2815_03076	-1.78	2.34E-03	D-galactose 1-dehydrogenase
NBC2815_03077	NBC2815_03077	-2.22	3.18E-06	ISXo1 transposase, IS5 family
NBC2815_03084	NBC2815_03084	-1.51	2.53E-02	acetyltransferase
nrdR	NBC2815_03085	-2.28	1.94E-08	transcriptional regulator NrdR
glyA	NBC2815_03086	-1.92	1.33E-04	glycine/serine hydroxymethyltransferase
NBC2815_03087	NBC2815_03087	3.59	7.29E-10	Hypothetical Protein
NBC2815_03091	NBC2815_03091	-1.57	2.58E-02	serine protease
NBC2815_03093	NBC2815_03093	-1.93	1.89E-02	is4 transposase (fragment) protein
NBC2815_03097	NBC2815_03097	1.51	1.05E-02	PnuC protein
xpsD	NBC2815_03100	-2.87	8.62E-10	general secretion pathway protein D
xpsK	NBC2815_03104	-2.17	5.52E-06	general secretion pathway protein K
NBC2815_03105	NBC2815_03105	-1.54	3.29E-02	general secretion pathway protein J
NBC2815_03106	NBC2815_03106	-1.65	2.51E-02	general secretion pathway protein I
NBC2815_03111	NBC2815_03111	-7.10	0.00E+00	Subtilisin-like serine protease
NBC2815_03112	NBC2815_03112	-8.12	0.00E+00	outer membrane protein XadA
purL	NBC2815_03115	-1.77	5.10E-04	phosphoribosylformylglycinamidine synthase
NBC2815_03118	NBC2815_03118	1.81	4.50E-02	RDD family protein
NBC2815_03119	NBC2815_03119	2.90	1.14E-12	permease
NBC2815_03120	NBC2815_03120	3.37	2.42E-13	permease
NBC2815_03121	NBC2815_03121	-1.72	8.64E-04	leucyl aminopeptidase /EC_number="3.4.11.1
NBC2815_03122	NBC2815_03122	-3.59	5.33E-13	DNA polymerase III subunit chi
valS	NBC2815_03123	-2.08	3.74E-06	valyl-tRNA synthetase
NBC2815_03125	NBC2815_03125	-2.22	2.12E-04	ISxac3 transposase
NBC2815_03126	NBC2815_03126	5.53	1.05E-02	hypothetical protein
NBC2815_03127	NBC2815_03127	1.61	5.68E-03	hypothetical protein
NBC2815_03129	NBC2815_03129	-2.21	1.18E-05	hypothetical protein
NBC2815_03131	NBC2815_03131	-1.84	2.01E-04	hypothetical protein
NBC2815_03133	NBC2815_03133	2.45	8.70E-08	IS1112 transposase
NBC2815_03138	NBC2815_03138	-2.41	2.39E-03	asparaginase
NBC2815_03143	NBC2815_03143	-2.10	5.09E-06	pilus biogenesis protein
pilI	NBC2815_03144	-2.03	1.14E-04	pilus biogenesis protein

gshB	NBC2815_03147	-2.70	7.57E-13	glutathione synthetase
NBC2815_03148	NBC2815_03148	-1.88	4.46E-05	TonB protein
NBC2815_03149	NBC2815_03149	-1.58	2.40E-03	putative glycoprotease
NBC2815_03154	NBC2815_03154	2.20	1.21E-07	Hypothetical Protein
NBC2815_03156	NBC2815_03156	1.87	1.76E-03	pyrroloquinoline quinone biosynthesis protein PqqB
NBC2815_03160	NBC2815_03160	2.51	7.38E-07	ATP-dependent RNA helicase
NBC2815_03163	NBC2815_03163	2.06	2.20E-05	Hypothetical Protein
NBC2815_03165	NBC2815_03165	1.68	6.07E-03	Hypothetical Protein
NBC2815_03166	NBC2815_03166	2.69	2.08E-10	pseudouridylate synthase
NBC2815_03167	NBC2815_03167	-1.58	8.54E-03	Hypothetical Protein
exsG	NBC2815_03168	-1.92	1.97E-02	two-component system sensor protein
NBC2815_03173	NBC2815_03173	1.89	4.57E-03	IS1404 transposase
NBC2815_03174	NBC2815_03174	1.93	1.69E-04	IS1404 transposase protein B
NBC2815_03176	NBC2815_03176	2.59	2.13E-09	glutathione transferase
NBC2815_03179	NBC2815_03179	2.94	2.35E-05	Hypothetical Protein
bfeA	NBC2815_03180	-1.77	6.40E-04	ferric enterobactin receptor
NBC2815_03181	NBC2815_03181	3.82	1.52E-14	trehalose-6-phosphate phosphatase
NBC2815_03182	NBC2815_03182	2.64	2.11E-08	glycosyl hydrolase family protein
NBC2815_03183	NBC2815_03183	2.22	1.02E-05	trehalose-6-phosphate synthase
NBC2815_03184	NBC2815_03184	-1.55	1.99E-03	IS1478 transposase
NBC2815_03185	NBC2815_03185	1.64	3.85E-03	glucose dehydrogenase
nadE	NBC2815_03189	-1.66	3.71E-04	NAD synthetase
NBC2815_03191	NBC2815_03191	-1.66	3.28E-03	succinyl-CoA synthetase subunit alpha /EC_number="6.2.1.5
sucC	NBC2815_03192	-2.24	1.20E-06	succinyl-CoA synthetase subunit beta
pilR	NBC2815_03194	-1.98	3.39E-06	two-component system regulatory protein
NBC2815_03195	NBC2815_03195	-2.38	9.82E-08	hypothetical protein
pilC	NBC2815_03200	-1.81	4.48E-04	fimbrial assembly protein
coaE	NBC2815_03202	-1.71	4.21E-04	dephospho-CoA kinase
NBC2815_03208	NBC2815_03208	2.25	1.30E-06	quinone reductase
metF1	NBC2815_03210	3.32	8.31E-10	methylenetetrahydrofolate reductase
NBC2815_03211	NBC2815_03211	1.83	3.11E-03	Hypothetical Protein
NBC2815_03212	NBC2815_03212	-2.28	1.90E-08	Thiamine monophosphate synthase ThiE
ftsZ	NBC2815_03218	-5.03	0.00E+00	cell division protein FtsZ
ftsA	NBC2815_03219	-5.06	2.45E-29	cell division protein ftsa
NBC2815_03220	NBC2815_03220	-4.19	1.50E-18	cell division protein
ddl_1	NBC2815_03221	-3.98	4.39E-25	D-alanine--D-alanine ligase
murC	NBC2815_03222	-4.48	0.00E+00	UDP-N-acetylmuramate--L-alanine ligase
murG	NBC2815_03223	-2.28	4.43E-09	undecaprenyldiphospho-muramoylpentapeptide beta-N-acetylglucosaminyltransferase
ftsW	NBC2815_03224	-3.85	2.66E-19	cell division protein FtsW
mraY	NBC2815_03225	-2.80	2.57E-14	phospho-N-acetylmuramoyl-pentapeptide- transferase
NBC2815_03226	NBC2815_03226	-2.23	2.00E-08	UDP-N-acetylmuramoylalanyl-D-glutamyl-2, 6-diaminopimelate/D-alanyl-D-alanyl ligase
murE	NBC2815_03227	-2.09	1.70E-07	UDP-N-acetylmuramoylalanyl-D-glutamate--2, 6-diaminopimelate ligase
ftsI	NBC2815_03228	-1.97	4.97E-06	penicillin-binding protein 3
NBC2815_03229	NBC2815_03229	-2.34	1.82E-04	cell division protein
mraW	NBC2815_03230	-1.82	3.14E-05	S-adenosyl-methyltransferase MraW
NBC2815_03231	NBC2815_03231	-2.10	7.28E-06	cell division protein MraZ
NBC2815_03234	NBC2815_03234	-4.85	1.51E-13	CcdB cytotoxin-like protein
NBC2815_03240	NBC2815_03240	-2.85	3.34E-04	IS1478 transposase
NBC2815_03241	NBC2815_03241	2.84	2.36E-05	IS1480 transposase
NBC2815_03242	NBC2815_03242	-3.45	2.82E-02	ISXo1 transposase, IS5 family
pilQ	NBC2815_03243	-4.22	0.00E+00	fimbrial assembly protein
NBC2815_03244	NBC2815_03244	-3.82	5.08E-20	fimbrial assembly protein
NBC2815_03245	NBC2815_03245	-3.92	2.66E-19	fimbrial assembly membrane protein
NBC2815_03246	NBC2815_03246	-2.68	5.52E-11	fimbrial assembly membrane protein
pilM	NBC2815_03247	-2.45	1.21E-08	fimbrial assembly membrane protein
NBC2815_03249	NBC2815_03249	1.87	2.36E-05	Hypothetical Protein
gltA	NBC2815_03250	-2.16	5.66E-06	type II citrate synthase
NBC2815_03252	NBC2815_03252	1.63	9.47E-04	inosine-uridine preferring nucleoside hydrolase
NBC2815_03257	NBC2815_03257	-1.60	1.08E-03	translation initiation inhibitor
spoT	NBC2815_03258	-1.79	1.69E-04	guanosine-3',5'-bis(diphosphate) 3'-pyrophosphohydrolase
rpoZ	NBC2815_03259	-1.99	1.49E-05	DNA-directed RNA polymerase subunit K
NBC2815_03261	NBC2815_03261	-2.56	2.63E-07	stress-induced protein
rph	NBC2815_03262	-1.54	4.95E-03	ribonuclease PH
NBC2815_03263	NBC2815_03263	-1.53	2.43E-02	deoxyribonucleotide triphosphate pyrophosphatase
NBC2815_03265	NBC2815_03265	3.88	3.67E-03	Hypothetical Protein
pepQ_2	NBC2815_03266	-1.98	4.36E-06	Xaa-Pro dipeptidase
pepP	NBC2815_03267	-2.07	3.00E-07	aminopeptidase
NBC2815_03269	NBC2815_03269	-3.31	1.26E-13	Hypothetical Protein
NBC2815_03270	NBC2815_03270	-1.60	6.93E-03	cell division protein ZapA
NBC2815_03273	NBC2815_03273	-2.70	5.60E-11	ribose-5-phosphate isomerase A /EC_number="5.3.1.6
NBC2815_03274	NBC2815_03274	-2.24	2.89E-04	Hypothetical Protein
NBC2815_03275	NBC2815_03275	-1.84	1.53E-05	Hypothetical Protein
NBC2815_03276	NBC2815_03276	-3.43	3.01E-09	Putative Rubredoxin
thiE	NBC2815_03277	-2.47	1.30E-04	thiamine-phosphate pyrophosphorylase
argD	NBC2815_03279	-1.84	2.89E-05	bifunctional acetylornithine aminotransferase, succinyldiaminopimelate aminotransferase
NBC2815_03281	NBC2815_03281	-2.74	1.68E-04	ISXo1 transposase, IS5 family
mpl	NBC2815_03287	-1.60	9.76E-04	UDP-N-acetylmuramate:L-alanyl-gamma-D-glutamyl- meso-diaminopimelate ligase
adk	NBC2815_03288	-2.68	4.80E-12	adenylate kinase
NBC2815_03289	NBC2815_03289	-1.80	3.99E-05	6-phosphofructokinase /EC_number="2.7.1.11
virB6_3	NBC2815_03290	2.52	1.62E-05	VirB6 protein
hppA	NBC2815_03292	-2.36	3.27E-08	membrane-bound proton-translocating pyrophosphatase
NBC2815_03294	NBC2815_03294	-2.39	7.34E-08	inorganic pyrophosphatase /EC_number="3.6.1.1
NBC2815_03296	NBC2815_03296	-2.28	5.54E-06	TonB-dependent receptor
NBC2815_03297	NBC2815_03297	-1.83	3.68E-03	TonB-dependent receptor
NBC2815_03300	NBC2815_03300	1.90	7.96E-06	regulatory protein
xopF1_2	NBC2815_03302	3.21	1.69E-08	type III effector protein XopF1
hpa3_2	NBC2815_03303	2.98	2.53E-04	Hpa3 protein, type III secretion system
NBC2815_03304	NBC2815_03304	3.36	1.02E-03	hypothetical protein
NBC2815_03306	NBC2815_03306	4.21	3.84E-04	methyl-accepting chemotaxis protein
NBC2815_03307	NBC2815_03307	5.91	1.16E-04	methyl-accepting chemotaxis protein
NBC2815_03308	NBC2815_03308	5.85	2.73E-06	methyl-accepting chemotaxis protein
NBC2815_03309	NBC2815_03309	4.97	1.05E-05	methyl-accepting chemotaxis protein
NBC2815_03311	NBC2815_03311	2.69	4.88E-08	acetolactate synthase 2 catalytic subunit /EC_number="2.2.1.6
NBC2815_03312	NBC2815_03312	1.69	2.20E-03	acetolactate synthase isozyme II small subunit

NBC2815_03313	NBC2815_03313	1.63	1.54E-03	threonine dehydratase /EC_number="4.3.1.19
leuB	NBC2815_03318	-1.58	7.82E-03	3-isopropylmalate dehydrogenase
leuD	NBC2815_03319	-1.95	2.63E-05	isopropylmalate isomerase small subunit
NBC2815_03324	NBC2815_03324	-1.66	3.47E-03	TolC protein
NBC2815_03328	NBC2815_03328	-1.61	4.76E-03	glycosyltransferase
NBC2815_03329	NBC2815_03329	-3.50	3.11E-13	hypothetical protein
oprO	NBC2815_03333	-4.40	0.00E+00	polyphosphate-selective porin O
NBC2815_03334	NBC2815_03334	-1.89	1.37E-05	sensor histidine kinase
NBC2815_03335	NBC2815_03335	-2.41	2.18E-03	Hypothetical Protein
NBC2815_03341	NBC2815_03341	-2.39	5.01E-09	hypothetical protein
NBC2815_03342	NBC2815_03342	-6.17	7.38E-18	hypothetical protein
NBC2815_03343	NBC2815_03343	-2.36	6.44E-07	hypothetical protein
NBC2815_03344	NBC2815_03344	-3.21	6.48E-11	hypothetical protein
raxB1	NBC2815_03345	-2.52	9.10E-10	peptide ABC transporter ATPase/permease
NBC2815_03347	NBC2815_03347	-1.59	2.51E-02	hypothetical protein
NBC2815_03349	NBC2815_03349	3.00	3.01E-02	ISxac3 transposase
NBC2815_03350	NBC2815_03350	1.61	5.34E-03	IS1112 transposase
tctD	NBC2815_03357	-1.96	8.61E-03	two-component system regulatory protein
NBC2815_03358	NBC2815_03358	2.54	1.68E-05	porin
cebR	NBC2815_03361	-1.76	5.95E-05	transcriptional regulator
suc1	NBC2815_03362	-2.58	6.12E-13	sugar transporter
fyuA	NBC2815_03363	-8.48	0.00E+00	TonB-dependent receptor
NBC2815_03364	NBC2815_03364	-5.16	0.00E+00	alpha-amlyase
NBC2815_03366	NBC2815_03366	2.00	4.17E-06	GTPase RsgA
NBC2815_03370	NBC2815_03370	-8.46	1.57E-08	hypothetical protein
NBC2815_03374	NBC2815_03374	-1.53	3.62E-03	Hypothetical Protein
NBC2815_03375	NBC2815_03375	-2.67	4.37E-05	methyltransferase
NBC2815_03376	NBC2815_03376	1.73	7.87E-03	polysaccharide deacetylase
NBC2815_03384	NBC2815_03384	-1.56	2.31E-03	IS1478 transposase
NBC2815_03385	NBC2815_03385	1.90	8.06E-05	Hypothetical Protein
NBC2815_03386	NBC2815_03386	2.12	3.81E-02	amidohydrolase family protein
NBC2815_03406	NBC2815_03406	2.60	1.34E-04	isxal1 transposase orf b protein
NBC2815_03410	NBC2815_03410	2.82	4.58E-04	type II secretion system protein M
NBC2815_03418	NBC2815_03418	-1.66	2.45E-03	two-component system regulatory protein
NBC2815_03421	NBC2815_03421	1.95	6.38E-05	putative oxidoreductase YvaA
NBC2815_03423	NBC2815_03423	2.75	7.46E-10	putative secreted protein
NBC2815_03424	NBC2815_03424	4.39	1.16E-13	Hypothetical Protein
NBC2815_03425	NBC2815_03425	1.87	5.43E-04	Hypothetical Protein
NBC2815_03426	NBC2815_03426	1.95	3.52E-02	Hypothetical Protein
NBC2815_03433	NBC2815_03433	-1.82	2.70E-05	hypothetical protein
NBC2815_03435	NBC2815_03435	1.81	8.69E-04	lipoyl synthase /EC_number="2.8.1.8
lipB	NBC2815_03436	2.52	6.60E-08	lipoate-protein ligase B
NBC2815_03437	NBC2815_03437	3.61	1.60E-11	Hypothetical Protein
NBC2815_03438	NBC2815_03438	2.25	1.21E-06	Hypothetical Protein
NBC2815_03439	NBC2815_03439	1.90	8.13E-03	hypothetical protein
NBC2815_03440	NBC2815_03440	-2.43	1.41E-08	penicillin-binding protein 6
mltB	NBC2815_03442	-1.87	2.53E-05	murein-degrading transglycosylase
NBC2815_03443	NBC2815_03443	1.88	1.13E-02	endopolygalacturonase
NBC2815_03447	NBC2815_03447	-1.57	2.32E-03	rod shape-determining protein MreC
mreB	NBC2815_03448	-1.51	1.27E-02	rod shape-determining protein MreB
NBC2815_03449	NBC2815_03449	-2.06	8.50E-07	sugar kinase
pepN	NBC2815_03452	-1.65	1.79E-03	aminopeptidase
ubiE	NBC2815_03454	-1.51	9.93E-03	ubiquinone/menaquinone biosynthesis methyltransferase
NBC2815_03455	NBC2815_03455	7.97	1.21E-36	major facilitator superfamily protein
NBC2815_03456	NBC2815_03456	5.97	2.59E-17	MFS transporter
NBC2815_03459	NBC2815_03459	1.95	9.94E-04	hypothetical protein
hslU	NBC2815_03460	1.59	3.65E-02	ATP-dependent protease ATP-binding subunit HslU
hslV	NBC2815_03461	2.94	4.01E-07	peptidase component of the hsluv protease
NBC2815_03463	NBC2815_03463	-2.05	1.08E-06	Hypothetical Protein
dapF	NBC2815_03464	-1.79	1.56E-04	diaminopimelate epimerase
ptrB	NBC2815_03466	-1.65	1.68E-02	oligopeptidase B
NBC2815_03467	NBC2815_03467	1.87	1.50E-03	aminotransferase
NBC2815_03469	NBC2815_03469	-4.71	1.25E-12	hypothetical protein
NBC2815_03470	NBC2815_03470	-4.19	0.00E+00	prolyl oligopeptidase
NBC2815_03474	NBC2815_03474	-1.69	2.46E-04	salt-induced outer membrane protein
hemC	NBC2815_03476	-1.59	1.69E-03	porphobilinogen deaminase
NBC2815_03485	NBC2815_03485	2.27	2.82E-02	methyl-accepting chemotaxis protein
NBC2815_03486	NBC2815_03486	3.67	1.43E-08	histidine kinase-response regulator hybrid protein
NBC2815_03487	NBC2815_03487	-2.79	1.58E-11	zinc protease
NBC2815_03491	NBC2815_03491	3.36	1.19E-08	Hypothetical Protein
NBC2815_03493	NBC2815_03493	-2.04	3.86E-05	Hypothetical Protein
corA_2	NBC2815_03495	-1.95	3.47E-06	magnesium/cobalt transporter CorA
NBC2815_03497	NBC2815_03497	1.57	1.35E-02	D-alanine/D-serine
NBC2815_03498	NBC2815_03498	2.34	2.45E-03	Hypothetical Protein
NBC2815_03500	NBC2815_03500	-3.35	1.30E-04	drug-metabolite transporter superfamily protein
NBC2815_03501	NBC2815_03501	-2.33	4.45E-09	archaeal methyltransferase
NBC2815_03503	NBC2815_03503	-4.75	2.14E-27	dipeptidyl peptidase IV /EC_number="3.4.14.11
NBC2815_03504	NBC2815_03504	-2.23	4.23E-08	oxidoreductase
NBC2815_03507	NBC2815_03507	1.52	3.60E-03	tis1421-transposase b
xopAD_5	NBC2815_03508	2.65	1.72E-11	type III effector protein XopAD
NBC2815_03509	NBC2815_03509	2.18	1.81E-02	hypothetical protein
NBC2815_03519	NBC2815_03519	-2.36	5.45E-08	3-dehydroquinate dehydratase /EC_number="4.2.1.10
NBC2815_03520	NBC2815_03520	-4.17	2.84E-18	acetyl-CoA carboxylase biotin carboxyl carrier protein subunit /EC_number="6.4.1.2
NBC2815_03521	NBC2815_03521	-4.89	4.03E-16	Hypothetical protein
accC	NBC2815_03522	-5.30	2.04E-28	acetyl-CoA carboxylase, biotin carboxylase subunit protein
NBC2815_03524	NBC2815_03524	2.98	4.63E-05	Hypothetical Protein
fis	NBC2815_03527	-2.59	3.18E-11	global DNA-binding transcriptional dual regulator Fis
NBC2815_03528	NBC2815_03528	4.73	7.60E-18	ice-nucleation proteins octamer repeat protein
NBC2815_03529	NBC2815_03529	6.09	1.83E-14	hypothetical protein
NBC2815_03531	NBC2815_03531	-1.60	1.46E-03	phosphoribosylamine--glycine ligase /EC_number="6.3.4.13
NBC2815_03537	NBC2815_03537	1.57	3.45E-02	2-acylglycerophosphoethanolamine acyltransferase
NBC2815_03538	NBC2815_03538	-2.53	9.49E-10	transmembrane protein
NBC2815_03543	NBC2815_03543	-2.42	1.20E-02	IS1479 transposase-like protein
NBC2815_03544	NBC2815_03544	-2.84	5.74E-05	transposase

NBC2815_03546	NBC2815_03546	-3.11	2.97E-04	ISxac3 transposase
NBC2815_03548	NBC2815_03548	3.39	5.51E-11	TetR family transcriptional regulator
NBC2815_03549	NBC2815_03549	2.57	1.37E-05	XopN effector
dcp2_2	NBC2815_03550	-2.70	8.15E-10	peptidyl-dipeptidase
NBC2815_03551	NBC2815_03551	-3.20	1.58E-04	IS1479 transposase
NBC2815_03552	NBC2815_03552	-1.97	8.97E-07	acyltransferase
NBC2815_03554	NBC2815_03554	-3.69	6.22E-11	Hypothetical Protein
NBC2815_03555	NBC2815_03555	-4.34	3.26E-07	pseudouridylate synthase
NBC2815_03556	NBC2815_03556	-4.17	3.83E-17	disulfide isomerase
mdmC	NBC2815_03557	-1.91	4.83E-02	O-methyltransferase
ubiB	NBC2815_03560	1.86	2.81E-04	ubiquinone biosynthesis protein UbiB
NBC2815_03561	NBC2815_03561	2.43	1.32E-07	sterol-binding domain-containing protein
ddl_2	NBC2815_03562	3.82	9.64E-16	D-alanyl-alanine synthetase A
ddl_3	NBC2815_03563	3.00	6.07E-09	D-alanyl-alanine synthetase A
NBC2815_03564	NBC2815_03564	3.03	5.82E-10	hypothetical protein
NBC2815_03565	NBC2815_03565	4.67	2.18E-14	Hypothetical Protein
NBC2815_03570	NBC2815_03570	-2.14	6.48E-07	ISXac3 transposase, IS3 family
NBC2815_03572	NBC2815_03572	7.07	5.84E-40	lipase
erpA	NBC2815_03573	3.07	2.21E-11	iron-sulfur cluster insertion protein
NBC2815_03575	NBC2815_03575	-1.54	1.29E-02	Hypothetical Protein
trpC	NBC2815_03590	-1.65	1.12E-03	indole-3-glycerol phosphate synthase
trpD	NBC2815_03591	-1.50	7.93E-03	anthranilate phosphoribosyltransferase
NBC2815_03594	NBC2815_03594	2.63	1.48E-07	integrase, catalytic region
NBC2815_03595	NBC2815_03595	2.59	6.06E-05	is4 transposase (fragment) protein
NBC2815_03598	NBC2815_03598	-2.67	6.44E-05	fatty acid desaturase
NBC2815_03599	NBC2815_03599	-2.75	1.52E-04	glutamine amidotransferases protein
RhtB	NBC2815_03600	-2.39	2.63E-04	threonine efflux protein
NBC2815_03602	NBC2815_03602	-3.12	2.88E-08	glutamine synthetase
trpE_2	NBC2815_03604	-1.51	1.06E-02	anthranilate synthase component I
NBC2815_03605	NBC2815_03605	2.33	1.34E-07	lipid kinase
NBC2815_03606	NBC2815_03606	1.93	2.10E-05	N-acetyltransferase
rpe	NBC2815_03607	-2.18	4.50E-08	ribulose-phosphate 3-epimerase
NBC2815_03609	NBC2815_03609	-1.97	4.91E-05	putative DnaJ-related chaperone
NBC2815_03610	NBC2815_03610	-4.66	3.22E-22	phosphoribosylaminoimidazole-succinocarboxamide synthase /EC_number="6.3.2.6
NBC2815_03611	NBC2815_03611	-2.59	5.50E-03	Zinc metalloprotease
NBC2815_03613	NBC2815_03613	-1.78	1.61E-02	membrane protein
NBC2815_03618	NBC2815_03618	2.83	1.18E-09	monovalent cation/H+ antiporter subunit A
phaC	NBC2815_03619	2.18	1.24E-03	monovalent cation/H+ antiporter subunit C
NBC2815_03620	NBC2815_03620	2.36	2.27E-06	monovalent cation/H+ antiporter subunit D
NBC2815_03621	NBC2815_03621	2.45	2.55E-03	monovalent cation/H+ antiporter subunit E
NBC2815_03625	NBC2815_03625	5.08	4.11E-17	HAD superfamily hydrolase
NBC2815_03626	NBC2815_03626	18.24	3.92E-33	Hypothetical Protein
NBC2815_03627	NBC2815_03627	4.15	2.60E-04	EF hand domain-containing protein
NBC2815_03631	NBC2815_03631	-1.89	6.16E-06	N-acetylmuramoyl-L-alanine amidase
NBC2815_03638	NBC2815_03638	2.11	2.76E-03	membrane protein
yliI	NBC2815_03639	2.42	5.37E-04	dehydrogenase
folB	NBC2815_03641	-2.21	1.82E-05	dihydroneopterin aldolase
rpsU	NBC2815_03643	-2.89	9.24E-11	30S ribosomal protein S21
NBC2815_03645	NBC2815_03645	2.80	1.67E-10	ribonuclease BN
NBC2815_03648	NBC2815_03648	-1.58	1.29E-02	Na+-dependent transporter
NBC2815_03650	NBC2815_03650	-2.12	4.98E-05	protoheme IX farnesyltransferase
NBC2815_03651	NBC2815_03651	-2.56	1.51E-09	cytochrome oxidase assembly protein
NBC2815_03653	NBC2815_03653	-1.54	6.66E-03	Hypothetical Protein
NBC2815_03654	NBC2815_03654	-3.00	5.79E-12	Hypothetical Protein
NBC2815_03655	NBC2815_03655	-2.35	4.80E-08	cytochrome C oxidase subunit III
NBC2815_03656	NBC2815_03656	-1.99	1.23E-05	cytochrome C oxidase assembly protein
NBC2815_03657	NBC2815_03657	-1.91	3.22E-04	Hypothetical Protein
NBC2815_03658	NBC2815_03658	-2.72	2.79E-09	cytochrome C oxidase, subunitItransmembrane protein /EC_number="1.9.3.1
NBC2815_03659	NBC2815_03659	-1.80	1.60E-04	cytochrome C oxidase subunit II
NBC2815_03660	NBC2815_03660	-3.45	0.00E+00	Hypothetical Protein
NBC2815_03661	NBC2815_03661	-18.00	0.00E+00	bifunctional proline dehydrogenase/pyrroline-5-carboxylate dehydrogenase
tyrS	NBC2815_03662	-3.13	2.01E-14	putative tyrosyl-tRNA synthetase
NBC2815_03666	NBC2815_03666	-1.89	3.52E-04	signal transducer
pyrE	NBC2815_03668	-3.14	1.26E-10	orotate phosphoribosyltransferase
NBC2815_03670	NBC2815_03670	-1.65	3.50E-04	chromosome partitioning protein
parB	NBC2815_03671	-1.57	3.30E-03	chromosome partitioning protein
NBC2815_03678	NBC2815_03678	2.43	2.19E-06	Hypothetical Protein
NBC2815_03691	NBC2815_03691	3.30	3.28E-02	hypothetical protein
NBC2815_03692	NBC2815_03692	1.85	4.72E-05	LysR family transcriptional regulator
NBC2815_03693	NBC2815_03693	9.07	1.21E-36	putative glucosyltransferase
entF	NBC2815_03694	7.55	9.47E-27	ATP-dependent serine activating enzyme
NBC2815_03696	NBC2815_03696	4.89	2.56E-03	IS1478 transposase
NBC2815_03697	NBC2815_03697	3.47	1.95E-12	two-component system sensor protein
phoP	NBC2815_03698	2.55	2.10E-06	two-component system regulatory protein phop
NBC2815_03699	NBC2815_03699	4.40	4.50E-18	Hypothetical Protein
NBC2815_03700	NBC2815_03700	3.45	5.03E-10	tRNA-dihydrouridine synthase A
NBC2815_03701	NBC2815_03701	56.86	7.57E-19	Hypothetical Protein
NBC2815_03703	NBC2815_03703	1.68	4.22E-02	putative secreted protein
NBC2815_03704	NBC2815_03704	-1.82	2.14E-02	IS1479 transposase-like protein
NBC2815_03708	NBC2815_03708	-1.55	3.18E-02	hypothetical protein
NBC2815_03710	NBC2815_03710	-1.96	5.29E-05	Hypothetical Protein
NBC2815_03712	NBC2815_03712	-2.10	9.58E-06	biotin acetyl-CoA-carboxylase synthetase
NBC2815_03716	NBC2815_03716	3.41	6.94E-04	IS1389 transposase
NBC2815_03717	NBC2815_03717	2.42	3.60E-05	cointegrate resolution protein T (fragment)
NBC2815_03720	NBC2815_03720	1.65	9.57E-03	Pectate lyase precursor /EC_number="4.2.2.2
NBC2815_03722	NBC2815_03722	3.76	8.20E-07	putative secreted protein
rpmB	NBC2815_03728	-1.54	3.28E-02	50S ribosomal protein L28
NBC2815_03729	NBC2815_03729	-2.14	9.10E-08	hypothetical protein
NBC2815_03730	NBC2815_03730	2.08	3.55E-04	cation efflux system protein
gidB	NBC2815_03731	-1.51	1.10E-02	methyltransferase GidB
NBC2815_03732	NBC2815_03732	2.32	3.00E-02	alkaline phosphatase
NBC2815_03734	NBC2815_03734	2.61	2.73E-07	transglycosylase associated protein
NBC2815_03735	NBC2815_03735	1.86	2.94E-04	Hypothetical Protein
NBC2815_03737	NBC2815_03737	-2.52	3.96E-03	hypothetical protein

actP	NBC2815_03738	-5.89	1.20E-34	acetate permease
NBC2815_03739	NBC2815_03739	-10.31	3.53E-28	membrane protein
NBC2815_03740	NBC2815_03740	-4.23	9.38E-26	Hypothetical Protein
acs	NBC2815_03741	-2.86	5.78E-11	acetyl-CoA synthetase
tcsR	NBC2815_03742	-2.03	8.41E-06	two-component system regulatory protein
pld	NBC2815_03743	-1.62	1.81E-03	pyridoxal 4-dehydrogenase
NBC2815_03744	NBC2815_03744	-1.50	1.35E-02	metal-dependent hydrolase
NBC2815_03745	NBC2815_03745	-3.79	2.97E-22	oxidoreductase
NBC2815_03746	NBC2815_03746	-5.43	1.87E-26	2-hydroxyhepta-2,4-diene-1,7-dioate isomerase
NBC2815_03747	NBC2815_03747	-4.92	1.57E-27	L-alanine-DL-glutamate epimerase of enolase superfamily protein
NBC2815_03748	NBC2815_03748	-8.01	1.73E-16	Hypothetical Protein
fucP	NBC2815_03749	-4.27	1.10E-25	fucose permease
yebN	NBC2815_03751	2.90	1.18E-03	membrane protein YebN
NBC2815_03752	NBC2815_03752	1.70	5.67E-04	two-component system sensor-response regulator hybrid protein
idnK	NBC2815_03755	1.96	3.85E-05	gluconokinase
NBC2815_03757	NBC2815_03757	3.30	1.09E-10	Hypothetical Protein
NBC2815_03758	NBC2815_03758	2.01	4.80E-05	pathogenicity protein
NBC2815_03764	NBC2815_03764	-1.61	3.81E-03	DNA topoisomerase I
NBC2815_03768	NBC2815_03768	-1.93	3.59E-05	hypothetical protein
NBC2815_03769	NBC2815_03769	-1.66	1.91E-03	protein smg
NBC2815_03771	NBC2815_03771	-2.81	4.83E-11	LysM domain-containing protein
def_2	NBC2815_03772	-2.27	2.39E-08	peptide deformylase
NBC2815_03781	NBC2815_03781	3.33	1.60E-10	GTP cyclohydrolase /EC_number="3.5.4.25
yncA	NBC2815_03782	-3.02	9.72E-08	phosphinothricin acetyltransferase
dtd	NBC2815_03784	1.71	4.50E-02	D-tyrosyl-tRNA(Tyr) deacylase
rpoD	NBC2815_03785	1.72	4.08E-03	RNA polymerase sigma factor RpoD
int_3	NBC2815_03788	1.69	7.83E-03	phage-related integrase
NBC2815_03799	NBC2815_03799	-1.94	1.98E-02	Hypothetical Protein
NBC2815_03813	NBC2815_03813	-2.23	1.13E-05	hypothetical protein
S	NBC2815_03814	2.02	1.79E-02	phage-related tail protein
NBC2815_03816	NBC2815_03816	2.63	3.61E-02	Hypothetical Protein
NBC2815_03825	NBC2815_03825	-44.97	8.47E-04	phage-related capsid scaffold protein
NBC2815_03829	NBC2815_03829	-2.90	1.53E-04	hypothetical protein
NBC2815_03830	NBC2815_03830	-3.78	4.16E-17	hypothetical protein
NBC2815_03831	NBC2815_03831	-3.59	8.67E-07	hypothetical protein
NBC2815_03832	NBC2815_03832	3.78	2.06E-10	VGR-like protein
NBC2815_03833	NBC2815_03833	2.68	3.18E-06	Rhs element Vgr protein
NBC2815_03837	NBC2815_03837	6.50	2.30E-27	VGR-like protein
NBC2815_03838	NBC2815_03838	5.83	6.31E-17	Rhs element Vgr protein
NBC2815_03839	NBC2815_03839	2.11	6.65E-03	hypothetical protein
iroN_2	NBC2815_03849	-12.12	0.00E+00	TonB-dependent receptor
NBC2815_03850	NBC2815_03850	-15.35	8.73E-77	tryptophan halogenase
prnA_2	NBC2815_03851	-6.55	2.09E-42	tryptophan halogenase
NBC2815_03853	NBC2815_03853	-2.34	8.77E-10	TonB-like protein
NBC2815_03854	NBC2815_03854	-2.60	7.75E-12	ABC transporter sodium permease
natA	NBC2815_03855	-2.58	2.03E-10	sodium ABC transporter ATP-binding protein
NBC2815_03856	NBC2815_03856	-2.21	4.32E-09	cysteine proteinase
NBC2815_03857	NBC2815_03857	-1.67	3.03E-04	phenol hydroxylase
NBC2815_03858	NBC2815_03858	-2.00	8.66E-07	ABC transporter ATP-binding protein
yadH	NBC2815_03859	-1.77	3.83E-05	permease
NBC2815_03860	NBC2815_03860	-2.93	6.21E-12	hypothetical protein
NBC2815_03861	NBC2815_03861	-2.54	2.89E-09	2-dehydropantoate 2-reductase /EC_number="1.1.1.169
NBC2815_03862	NBC2815_03862	-3.30	1.22E-04	lipoprotein
NBC2815_03864	NBC2815_03864	1.72	5.89E-03	IS1478 transposase
NBC2815_03866	NBC2815_03866	-9.35	4.49E-14	ISXoc2 transposase, IS3 family
hmgA	NBC2815_03867	-5.65	4.70E-33	homogentisate 1,2-dioxygenase
NBC2815_03868	NBC2815_03868	-6.02	0.00E+00	4-hydroxyphenylpyruvate dioxygenase
NBC2815_03869	NBC2815_03869	-2.28	1.79E-09	transcriptional regulator, MarR family
yhiP	NBC2815_03872	-2.35	5.21E-09	di-tripeptide transporter
NBC2815_03873	NBC2815_03873	-1.51	1.93E-02	tryptophan 2,3-dioxygenase
NBC2815_03875	NBC2815_03875	-2.26	2.54E-05	Pyruvate dehydrogenase e1 component subunit alpha
NBC2815_03876	NBC2815_03876	-2.40	3.53E-03	Pyruvate dehydrogenase e1 component subunit alpha
NBC2815_03877	NBC2815_03877	-4.00	7.77E-12	pyruvate dehydrogenase E1 component
NBC2815_03878	NBC2815_03878	-2.84	1.69E-03	variant SH3 domain protein
NBC2815_03879	NBC2815_03879	-2.63	5.92E-08	branched-chain alpha-keto acid dehydrogenase subunit E2
NBC2815_03880	NBC2815_03880	-1.76	1.52E-04	ATP-dependent RNA helicase
NBC2815_03881	NBC2815_03881	-1.90	9.62E-04	Hypothetical Protein
NBC2815_03887	NBC2815_03887	2.52	1.90E-06	tis1421-transposase a
NBC2815_03888	NBC2815_03888	5.22	7.83E-20	pyridine nucleotide-disulfide oxidoreductase, putative
NBC2815_03889	NBC2815_03889	3.11	1.44E-06	3-oxoacyl-ACP reductase
NBC2815_03890	NBC2815_03890	3.05	4.83E-04	3-oxoacyl-ACP reductase
benE	NBC2815_03895	2.44	1.66E-03	benzoate permease
NBC2815_03897	NBC2815_03897	2.44	9.46E-04	phytochrome-like protein
NBC2815_03898	NBC2815_03898	2.93	1.56E-03	phytochrome-like protein
NBC2815_03899	NBC2815_03899	2.27	1.92E-02	heme oxygenase
NBC2815_03900	NBC2815_03900	5.47	6.25E-06	drug:H+ antiporter-1 family protein
NBC2815_03901	NBC2815_03901	2.76	8.98E-04	tetracycline-efflux transporter
NBC2815_03902	NBC2815_03902	1.77	5.38E-04	epimerase
NBC2815_03903	NBC2815_03903	3.89	7.44E-17	Hypothetical Protein
NBC2815_03904	NBC2815_03904	-2.59	8.42E-06	Hypothetical Protein
NBC2815_03906	NBC2815_03906	4.46	5.41E-06	hypothetical protein
NBC2815_03908	NBC2815_03908	2.02	9.83E-04	ATP-binding protein
NBC2815_03911	NBC2815_03911	2.29	1.08E-03	Hypothetical Protein
NBC2815_03915	NBC2815_03915	4.77	1.04E-04	hypothetical protein
NBC2815_03917	NBC2815_03917	1.94	4.30E-04	hypothetical protein
NBC2815_03919	NBC2815_03919	-1.65	1.64E-02	plasmid stabilization system protein, RelE-ParE family
NBC2815_03924	NBC2815_03924	-1.57	1.06E-02	IS1478 transposase
NBC2815_03926	NBC2815_03926	1.55	5.20E-03	lipoprotein
NBC2815_03927	NBC2815_03927	-7.74	1.04E-13	Hypothetical Protein
yrbF	NBC2815_03933	1.66	3.16E-03	toluene tolerance protein
NBC2815_03936	NBC2815_03936	-1.74	2.44E-03	toluene tolerance protein
NBC2815_03939	NBC2815_03939	-4.99	3.44E-29	FRG domain protein
NBC2815_03940	NBC2815_03940	-3.69	9.29E-22	glutathione peroxidase
NBC2815_03941	NBC2815_03941	2.70	7.59E-06	lipoprotein

NBC2815_03942	NBC2815_03942	-1.64	3.04E-04	hypothetical protein
NBC2815_03943	NBC2815_03943	5.08	2.48E-02	Hypothetical Protein
NBC2815_03946	NBC2815_03946	2.85	5.82E-10	putative signal protein with GAF, PAS(PAC) and GGDEF domains
NBC2815_03947	NBC2815_03947	1.64	1.10E-03	glycerate kinase
NBC2815_03950	NBC2815_03950	-1.59	2.25E-03	oxidoreductase
NBC2815_03951	NBC2815_03951	1.68	3.99E-04	export protein
glpQ	NBC2815_03952	2.34	1.17E-03	periplasmic glycerophosphodiester phosphodiesterase
trmE	NBC2815_03957	1.90	4.65E-05	tRNA modification GTPase TrmE
rpmH	NBC2815_03960	1.69	5.21E-03	50S ribosomal protein L34
NBC2815_04001	NBC2815_04001	3.51	1.06E-15	Putative replication protein
NBC2815_04003	NBC2815_04003	1.95	2.13E-02	hypothetical protein
traG	NBC2815_04004	2.37	2.17E-07	Type IV secretion system protein VirD4
NBC2815_04005	NBC2815_04005	2.16	2.54E-07	hypothetical protein
NBC2815_04008	NBC2815_04008	6.75	2.43E-23	hypothetical protein
NBC2815_04009	NBC2815_04009	6.56	5.49E-28	Site-specific recombinase
NBC2815_04010	NBC2815_04010	2.18	1.36E-04	Plasmid partition protein
NBC2815_04011	NBC2815_04011	1.70	3.13E-02	hypothetical protein
NBC2815_04020	NBC2815_04020	-1.64	9.83E-04	Hypothetical Protein
NBC2815_04022	NBC2815_04022	3.19	5.26E-05	hypothetical protein
NBC2815_04023	NBC2815_04023	1.87	1.33E-04	hypothetical protein
NBC2815_04024	NBC2815_04024	2.30	1.97E-06	Hypothetical protein
NBC2815_04026	NBC2815_04026	3.20	6.94E-10	hypothetical protein
NBC2815_04027	NBC2815_04027	4.13	6.68E-15	Site-specific recombinase
NBC2815_04028	NBC2815_04028	4.42	1.91E-06	hypothetical protein
NBC2815_04031	NBC2815_04031	-2.59	2.88E-12	partition protein C

* Values below 0 mean that the gene has a lower expression *in planta* than in Wilbrink's medium; values over 0 mean that the gene has higher expression *in planta* than in Wilbrink's medium

Table S3. Enriched COG/eggNOG categories among commonly up- and down-regulated genes of *X. fragariae in planta* vs. in pure bacterial culture

**COG/eggNOG**	**Enrichment ratio***	**FDR-derived *p*-values**
Over-represented COGs in up-regulated genes		
N - cell motility	3.03	2.53E-19
T - signal transduction mechanisms	1.58	2.73E-05
P - inorganic ion transport and metabolism	1.29	0.021
K - transcription	1.27	0.029
G - carbohydrate transport and metabolism	1.22	0.0325
Over-represented COGs in down-regulated genes		
C - energy production and conversion	1.75	3.73E-06
I - lipid transport and metabolism	1.95	3.73E-06
F - nucleotide transport and metabolism	1.90	0.13E-03
E - amino acid transport and metabolism	1.50	0.15E-03
J - translation	1.51	0.42E-03
U - intracellular trafficking and secretion	1.48	0.75E-02
M - cell wall/membrane/envelope biogenesis	1.16	0.049

*Enrichment ratio = proportion of COG X in the group of analysed set of *X. fragariae* differentially expressed genes/ proportion of COG X in *X. fragariae* genome

Table S4. Enriched secondary KEGG pathways among commonly up- and down-regulated genes of *X. fragariae in planta* vs. in pure bacterial culture

**Secondary KEGG pathway**	**Enrichment ratio***	**FDR-derived *p*-values**
Over-represented KEGGs among up-regulated genes		
Flagellar assembly	4.45	9.86E-17
Two-component system	1.95	0.73E-03
Bacterial chemotaxis	4.45	0.97E-03
Sulfur metabolism	3.18	0.28E-02
Over-represented KEGGs among down- regulated genes		
Oxidative phosphorylation	2.65	3.35E-13
Purine metabolism	1.64	8.913E-03
Aminoacyl-tRNA biosynthesis	2.00	1.515E-02

*Enrichment ratio = proportion of secondary KEGG pathway X in the group of analysed set of *X. fragariae* differentially expressed genes/ proportion of secondary KEGG pathway X in *X. fragariae* genome

Table S5. Top 20 up-regulated *X. fragariae* genes *in planta*

**Gene**	**Fold change**	**FDR *p*-value correction**	**Product**
NBC2815_00636	105.9199	6.89E-65	NADPH-sulfite reductase flavoprotein subunit
flhF	62.35954	1.37E-42	flagellar gtp-binding protein flhf
NBC2815_02003	58.87432	6.6E-38	Hypothetical Protein
NBC2815_03701	56.85988	7.57E-19	Hypothetical Protein
NBC2815_01996	51.0737	5.51E-22	flagellar motor protein MotD
cysI	50.8429	3.4E-71	sulfite reductase (NADPH) hemoprotein subunit beta
ahpF	45.66229	1.33E-85	alkyl hydroperoxide reductase
NBC2815_01818	43.42204	1.32E-30	RNA polymerase sigma factor
NBC2815_01850	43.38369	3.1E-22	flagellar protein
NBC2815_00181	42.11746	1.39E-56	ankyrin-like protein
NBC2815_01848	40.61609	5.26E-25	flagellar protein
NBC2815_02014	39.01692	1.96E-22	chemotaxis protein methyltransferase
flgJ	37.95125	1.37E-34	flagellar rod assembly protein/muramidase FlgJ
NBC2815_01563	37.33584	0.012479	transposase
flgE	35.56915	3.92E-25	flagellar hook protein FlgE
flgI	32.73334	1.52E-43	flagellar basal body P-ring protein
flgF	32.11444	4.75E-25	flagellar basal body rod protein FlgF
NBC2815_01999	31.43577	2.16E-20	anti-sigma factor antagonist-like protein
NBC2815_00180	30.91575	4.57E-29	catalase

Table S6. Top 20 down-regulated *X. fragariae* genes *in planta*

**Gene**	**Fold change**	**FDR p-value correction**	**Product**
NBC2815_03825	-44.9739	0.000847	phage-related capsid scaffold protein
pilW	-30.6205	1.15E-56	type IV pilus assembly protein PilW
NBC2815_01578	-21.6124	0	PilY1 protein
NBC2815_02806	-19.2106	0	ComEA-related DNA uptake protein
NBC2815_03661	-18.0018	0	bifunctional proline dehydrogenase/pyrroline-5-carboxylate dehydrogenase
NBC2815_00894	-16.3023	0.102496	ISxac3 transposase
NBC2815_03850	-15.3451	8.73E-77	tryptophan halogenase
pilE	-12.9492	8.3E-39	type IV pilin pile protein
iroN_2	-12.1184	0	TonB-dependent receptor
NBC2815_01451	-10.4176	4.4E-13	hypothetical protein
NBC2815_03739	-10.3094	3.53E-28	membrane protein
NBC2815_03866	-9.34617	4.49E-14	ISXoc2 transposase, IS3 family
fyuA	-8.4841	0	TonB-dependent receptor
NBC2815_03370	-8.4602	1.57E-08	hypothetical protein
NBC2815_00985	-8.33924	6.18E-26	hypothetical protein
NBC2815_03112	-8.11521	0	outer membrane protein XadA
NBC2815_03748	-8.01224	1.73E-16	Hypothetical Protein
NBC2815_01442	-7.79861	5.82E-24	acyl-CoA dehydrogenase
NBC2815_03927	-7.74074	1.04E-13	Hypothetical Protein
NBC2815_02244	-7.62174	1.66E-15	GTP cyclohydrolase

Table S7. Genes of *X. fragariae* IPO 3485 coding for degradative enzymes and their expression fold change *in planta*

Gene	Locus tag in IPO 3485 genome	product	Fold change*	FDR p-value correction
NBC2815_00007	NBC2815_00007	putative metal-dependent membrane protease	1.16	3.93E-01
NBC2815_00008	NBC2815_00008	Zn-dependent protease with chaperone functionHtpX	1.14	5.75E-01
ctp	NBC2815_00025	carboxyl-terminal protease	-1.85	4.49E-04
NBC2815_00083	NBC2815_00083	glucan 1,4-beta-glucosidase	1.47	3.54E-02
NBC2815_00084	NBC2815_00084	glucan 1,4-beta-glucosidase	10.10	1.34E-03
NBC2815_00106	NBC2815_00106	cellulase	1.52	8.85E-03
NBC2815_00107	NBC2815_00107	cellulase	5.19	1.05E-20
NBC2815_00119	NBC2815_00119	alpha-amylase	1.82	2.53E-03
NBC2815_00211	NBC2815_00211	protease Do	1.97	4.79E-04
NBC2815_00320	NBC2815_00320	Pectate lyase precursor	-1.26	1.37E-01
NBC2815_00320	NBC2815_00320	Pectate lyase precursor	-1.26	1.37E-01
NBC2815_00328	NBC2815_00328	metalloprotease	-1.32	2.74E-01
NBC2815_00429	NBC2815_00429	lipase	1.06	7.74E-01
NBC2815_00514	NBC2815_00514	lipase	-1.63	4.32E-03
NBC2815_00659	NBC2815_00659	cell wall hydrolase superfamily protein	9.98	1.85E-21
NBC2815_00676	NBC2815_00676	non-hemolytic phospholipase C	1.46	8.21E-02
glgX2	NBC2815_00679	isoamylase	1.90	3.68E-04
gloA	NBC2815_00776	lactoylglutathione lyase	-1.18	3.30E-01
NBC2815_00847	NBC2815_00847	zinc metalloprotease	-1.24	4.22E-01
NBC2815_00879	NBC2815_00879	Zn-dependent protease	2.00	2.59E-05
NBC2815_00954	NBC2815_00954	integral membrane protease subunit	1.42	7.38E-02
NBC2815_00999	NBC2815_00999	phospholipase A1	1.42	4.71E-02
clpX	NBC2815_01065	ATP-dependent clp protease ATP-binding subunit protein	1.18	4.22E-01
clpP	NBC2815_01066	ATP-dependent clp protease proteolytic subunit protein	2.35	4.01E-05
sdaA_1	NBC2815_01252	L-serine ammonia-lyase	1.81	2.90E-01
sdaA_2	NBC2815_01253	L-serine ammonia-lyase	-2.11	1.17E-02
sdaA_3	NBC2815_01254	L-serine ammonia-lyase	-1.67	6.94E-04
xsa_1	NBC2815_01342	xylosidase	3.53	5.10E-02
xsa_2	NBC2815_01343	xylosidase	2.82	1.25E-04
NBC2815_01365	NBC2815_01365	membrane-associated zinc metalloprotease	1.50	1.29E-02
NBC2815_01417	NBC2815_01417	phospholipase	1.27	3.54E-01
NBC2815_01432	NBC2815_01432	photolyase	-1.24	2.23E-01
mucD	NBC2815_01451	periplasmic protease	1.28	2.41E-01
NBC2815_01471	NBC2815_01471	ClpXP protease specificity-enhancing factor	-1.60	4.09E-03
NBC2815_01494	NBC2815_01494	Deoxyribodipyrimidine photolyase-like protein	-1.03	9.24E-01
NBC2815_01659	NBC2815_01659	subtilase serine protease	2.03	4.18E-03
NBC2815_01660	NBC2815_01660	subtilase serine protease	3.30	6.01E-11
hutH	NBC2815_01777	histidine ammonia-lyase	1.22	2.28E-01
NBC2815_01801	NBC2815_01801	autotransporter serine protease	22.60	3.73E-35
clpS	NBC2815_01877	ATP-dependent Clp protease adaptor protein ClpS	1.25	1.51E-01
clpA	NBC2815_01878	ATP-dependent clp protease ATP-binding subunit Clpa protein	5.13	1.21E-15
NBC2815_02177	NBC2815_02177	serine protease	4.10	3.23E-16
celD	NBC2815_02229	glucan 1,4-beta-glucosidase precursor	-3.38	2.11E-13
NBC2815_02249	NBC2815_02249	alpha-xylosidase	1.21	2.33E-01
NBC2815_02289	NBC2815_02289	Pectate lyase precursor	1.78	2.37E-03
NBC2815_02289	NBC2815_02289	Pectate lyase precursor	1.78	2.37E-03
NBC2815_02481	NBC2815_02481	pectate lyase	2.00	2.98E-03
NBC2815_02481	NBC2815_02481	pectate lyase	2.00	2.98E-03
NBC2815_02482	NBC2815_02482	polygalacturonase	2.94	2.00E-08
NBC2815_02499	NBC2815_02499	esterase/lipase/thioesterase	-1.38	3.87E-02
NBC2815_02616	NBC2815_02616	cellulase	-1.26	1.82E-01
NBC2815_02635	NBC2815_02635	extracellular protease	1.83	2.16E-04
NBC2815_02635	NBC2815_02635	extracellular protease	1.83	2.16E-04
NBC2815_02720	NBC2815_02720	extracellular serine protease	3.74	8.69E-08
NBC2815_02720	NBC2815_02720	extracellular serine protease	3.74	8.69E-08
NBC2815_02722	NBC2815_02722	serine protease	3.92	1.07E-18
NBC2815_02722	NBC2815_02722	serine protease	3.92	1.07E-18
NBC2815_02723	NBC2815_02723	serine protease	2.22	1.35E-06
NBC2815_02723	NBC2815_02723	serine protease	2.22	1.35E-06
NBC2815_02726	NBC2815_02726	cellulase	15.10	3.83E-13
NBC2815_02732	NBC2815_02732	cysteine protease	7.19	6.93E-41
hflK	NBC2815_02867	integral membrane protease subunit	-1.11	5.90E-01
NBC2815_02976	NBC2815_02976	extracellular protease	9.00	7.63E-13
NBC2815_02976	NBC2815_02976	extracellular protease	9.00	7.63E-13
NBC2815_02977	NBC2815_02977	extracellular protease	5.23	6.84E-05
NBC2815_02977	NBC2815_02977	extracellular protease	5.23	6.84E-05
NBC2815_02978	NBC2815_02978	extracellular protease	2.39	5.34E-02
NBC2815_02978	NBC2815_02978	extracellular protease	2.39	5.34E-02
NBC2815_02979	NBC2815_02979	extracellular protease	1.03	1.00E+00
NBC2815_02979	NBC2815_02979	extracellular protease	1.03	1.00E+00
NBC2815_03054	NBC2815_03054	Lysophospholipase L1	2.45	1.99E-02
NBC2815_03055	NBC2815_03055	Lysophospholipase L1	1.60	2.56E-01
NBC2815_03075	NBC2815_03075	alpha-amylase (fragment)	3.72	3.80E-11

NBC2815_03091	NBC2815_03091	serine protease	-1.57	2.58E-02
NBC2815_03111	NBC2815_03111	Subtilisin-like serine protease	-7.10	0.00E+00
NBC2815_03114	NBC2815_03114	Subtilisin-like serine protease	-1.15	3.83E-01
NBC2815_03149	NBC2815_03149	putative glycoprotease	-1.58	2.40E-03
NBC2815_03286	NBC2815_03286	ATP-dependent protease La (LON) domain subfamily protein	1.03	9.56E-01
NBC2815_03364	NBC2815_03364	alpha-amlyase	-5.16	0.00E+00
NBC2815_03376	NBC2815_03376	polysaccharide deacetylase	1.73	7.87E-03
NBC2815_03389	NBC2815_03389	cellulase S	1.50	2.77E-01
celS	NBC2815_03391	cellulase S	1.53	1.55E-01
prc	NBC2815_03429	Periplasmic protease	1.29	2.31E-01
NBC2815_03443	NBC2815_03443	endopolygalacturonase	1.88	1.13E-02
hslU	NBC2815_03460	ATP-dependent protease ATP-binding subunit HslU	1.59	3.65E-02
hslV	NBC2815_03461	peptidase component of the hsluv protease	2.94	4.01E-07
engXCA	NBC2815_03482	cellulase	1.14	4.51E-01
NBC2815_03487	NBC2815_03487	zinc protease	-2.79	1.58E-11
NBC2815_03487	NBC2815_03487	zinc protease	-2.79	1.58E-11
NBC2815_03572	NBC2815_03572	lipase	7.07	5.84E-40
NBC2815_03611	NBC2815_03611	Zinc metalloprotease	-2.59	5.50E-03
NBC2815_03612	NBC2815_03612	Zinc metalloprotease	-1.46	6.61E-01
NBC2815_03640	NBC2815_03640	beta-glucosidase	1.14	4.82E-01
NBC2815_03663	NBC2815_03663	membrane-anchored metalloprotease	-1.10	5.83E-01
NBC2815_03720	NBC2815_03720	Pectate lyase precursor	1.65	9.57E-03
NBC2815_03720	NBC2815_03720	Pectate lyase precursor	1.65	9.57E-03

Table S8. KEGG orthology assignments for genes located on *X. fragariae* NBC 2815 genome	
**Locus tag in NBC 2815 genome**	**KEGG term**
NBC2815_00001	K02313
NBC2815_00002	K02338
NBC2815_00005	K03629
NBC2815_00006	K02470
NBC2815_00007	K07052
NBC2815_00010	K03832
NBC2815_00011	K03561
NBC2815_00012	K03559
NBC2815_00013	K03559
NBC2815_00015	K07481
NBC2815_00017	K06131
NBC2815_00019	K03474
NBC2815_00020	K07481
NBC2815_00021	K06076
NBC2815_00024	K00058
NBC2815_00025	K03797
NBC2815_00026	K22719
NBC2815_00031	K03777
NBC2815_00033	K08081
NBC2815_00035	K04773
NBC2815_00036	K03327
NBC2815_00039	K04066
NBC2815_00041	K03089
NBC2815_00042	K03648
NBC2815_00044	K09811
NBC2815_00045	K09812
NBC2815_00046	K03732
NBC2815_00047	K03671
NBC2815_00048	K03628
NBC2815_00050	K00031
NBC2815_00054	K06879
NBC2815_00055	K07799
NBC2815_00056	K07788
NBC2815_00058	K07789
NBC2815_00065	K03670
NBC2815_00066	K00857
NBC2815_00067	K03656
NBC2815_00069	K09915
NBC2815_00071	K01591
NBC2815_00076	K00631
NBC2815_00080	K07504
NBC2815_00083	K05349
NBC2815_00086	K01235
NBC2815_00087	K02529
NBC2815_00088	K14058
NBC2815_00089	K03554
NBC2815_00090	K07043
NBC2815_00092	K01772

NBC2815_00094	K03116
NBC2815_00095	K03117
NBC2815_00096	K03118
NBC2815_00098	K01951
NBC2815_00099	K02454
NBC2815_00100	K01878
NBC2815_00101	K01879
NBC2815_00103	K00265
NBC2815_00104	K00266
NBC2815_00105	K13979
NBC2815_00106	K01179
NBC2815_00107	K01179
NBC2815_00108	K07481
NBC2815_00119	K16147
NBC2815_00120	K05343
NBC2815_00121	K00700
NBC2815_00122	K00064
NBC2815_00123	K03929
NBC2815_00125	K02554
NBC2815_00129	K02529
NBC2815_00132	K01815
NBC2815_00133	K00065
NBC2815_00135	K00500
NBC2815_00136	K03719
NBC2815_00137	K02013
NBC2815_00139	K05847
NBC2815_00140	K05846
NBC2815_00145	K04090
NBC2815_00147	K07088
NBC2815_00148	K03496
NBC2815_00152	K19222
NBC2815_00153	K00655
NBC2815_00154	K07019
NBC2815_00158	K04565
NBC2815_00159	K04565
NBC2815_00160	K07712
NBC2815_00161	K07708
NBC2815_00162	K03320
NBC2815_00163	K04751
NBC2815_00164	K01915
NBC2815_00165	K06153
NBC2815_00167	K07481
NBC2815_00171	K07481
NBC2815_00174	K01698
NBC2815_00175	K00380
NBC2815_00177	K00014
NBC2815_00179	K03722
NBC2815_00180	K03781
NBC2815_00181	K06867
NBC2815_00182	K07481

NBC2815_00184	K07481
NBC2815_00186	K02004
NBC2815_00188	K05786
NBC2815_00192	K01867
NBC2815_00196	K01476
NBC2815_00199	K07481
NBC2815_00200	K00943
NBC2815_00202	K12262
NBC2815_00203	K03781
NBC2815_00204	K01255
NBC2815_00205	K20862
NBC2815_00211	K04771
NBC2815_00216	K02483
NBC2815_00219	K07071
NBC2815_00224	K06889
NBC2815_00228	K14415
NBC2815_00229	K22105
NBC2815_00231	K00508
NBC2815_00234	K03098
NBC2815_00235	K07391
NBC2815_00236	K09806
NBC2815_00237	K04751
NBC2815_00238	K01197
NBC2815_00241	K00797
NBC2815_00243	K01585
NBC2815_00244	K07481
NBC2815_00246	K11902
NBC2815_00248	K02335
NBC2815_00249	K00228
NBC2815_00255	K18240
NBC2815_00257	K02078
NBC2815_00259	K00655
NBC2815_00266	K00059
NBC2815_00270	K00647
NBC2815_00271	K09902
NBC2815_00272	K06867
NBC2815_00275	K00036
NBC2815_00276	K11745
NBC2815_00278	K01674
NBC2815_00279	K06213
NBC2815_00280	K09181
NBC2815_00281	K09799
NBC2815_00282	K00525
NBC2815_00283	K00526
NBC2815_00285	K03671
NBC2815_00286	K11068
NBC2815_00288	K06877
NBC2815_00289	K07502
NBC2815_00301	K19048
NBC2815_00306	K07497

NBC2815_00307	K07483
NBC2815_00308	K00209
NBC2815_00310	K00464
NBC2815_00314	K00101
NBC2815_00315	K01971
NBC2815_00316	K01971
NBC2815_00318	K00832
NBC2815_00319	K03841
NBC2815_00335	K00626
NBC2815_00337	K02498
NBC2815_00338	K02496
NBC2815_00339	K01719
NBC2815_00343	K03071
NBC2815_00344	K00057
NBC2815_00346	K00156
NBC2815_00349	K03216
NBC2815_00351	K22317
NBC2815_00354	K07483
NBC2815_00355	K07497
NBC2815_00356	K19130
NBC2815_00357	K19129
NBC2815_00358	K19128
NBC2815_00359	K19127
NBC2815_00360	K07012
NBC2815_00361	K15342
NBC2815_00363	K01968
NBC2815_00364	K01969
NBC2815_00365	K00253
NBC2815_00370	K07004
NBC2815_00375	K07018
NBC2815_00376	K06916
NBC2815_00377	K04063
NBC2815_00379	K06222
NBC2815_00380	K23256
NBC2815_00383	K06181
NBC2815_00387	K03579
NBC2815_00391	K03424
NBC2815_00392	K08224
NBC2815_00395	K01433
NBC2815_00396	K07664
NBC2815_00400	K07481
NBC2815_00401	K07497
NBC2815_00402	K07483
NBC2815_00409	K01687
NBC2815_00411	K19267
NBC2815_00413	K00549
NBC2815_00420	K07497
NBC2815_00421	K07483
NBC2815_00423	K00864
NBC2815_00424	K02440

NBC2815_00425	K00111
NBC2815_00426	K02444
NBC2815_00428	K07178
NBC2815_00429	K01066
NBC2815_00430	K03495
NBC2815_00432	K01754
NBC2815_00435	K12979
NBC2815_00436	K02169
NBC2815_00438	K00059
NBC2815_00439	K02170
NBC2815_00441	K00652
NBC2815_00442	K01012
NBC2815_00444	K03179
NBC2815_00447	K05802
NBC2815_00449	K09781
NBC2815_00452	K00703
NBC2815_00453	K00700
NBC2815_00454	K01236
NBC2815_00455	K00705
NBC2815_00456	K06044
NBC2815_00458	K01214
NBC2815_00460	K07483
NBC2815_00461	K07497
NBC2815_00467	K07497
NBC2815_00469	K04084
NBC2815_00470	K03926
NBC2815_00473	K04078
NBC2815_00474	K04077
NBC2815_00478	K01626
NBC2815_00479	K18118
NBC2815_00481	K01053
NBC2815_00483	K00982
NBC2815_00484	K09125
NBC2815_00485	K07276
NBC2815_00486	K09019
NBC2815_00488	K07481
NBC2815_00497	K07481
NBC2815_00499	K13929
NBC2815_00500	K13931
NBC2815_00502	K13932
NBC2815_00503	K13933
NBC2815_00504	K13934
NBC2815_00505	K13930
NBC2815_00507	K00645
NBC2815_00512	K07315
NBC2815_00516	K00163
NBC2815_00522	K07497
NBC2815_00527	K03281
NBC2815_00529	K03321
NBC2815_00533	K01990

NBC2815_00534	K01992
NBC2815_00535	K01992
NBC2815_00536	K02014
NBC2815_00550	K06147
NBC2815_00552	K01854
NBC2815_00554	K01784
NBC2815_00561	K13924
NBC2815_00563	K07217
NBC2815_00569	K07386
NBC2815_00573	K21929
NBC2815_00578	K03316
NBC2815_00580	K07481
NBC2815_00588	K03924
NBC2815_00591	K07114
NBC2815_00592	K07114
NBC2815_00594	K03309
NBC2815_00595	K00615
NBC2815_00596	K16088
NBC2815_00597	K21472
NBC2815_00603	K02017
NBC2815_00604	K02018
NBC2815_00605	K02020
NBC2815_00607	K05986
NBC2815_00609	K00134
NBC2815_00615	K04487
NBC2815_00622	K00927
NBC2815_00623	K01091
NBC2815_00624	K00873
NBC2815_00625	K01623
NBC2815_00626	K23518
NBC2815_00627	K01738
NBC2815_00628	K02302
NBC2815_00633	K02014
NBC2815_00635	K00390
NBC2815_00636	K00381
NBC2815_00637	K00380
NBC2815_00638	K00957
NBC2815_00639	K00955
NBC2815_00640	K03585
NBC2815_00641	K18138
NBC2815_00642	K07483
NBC2815_00643	K07497
NBC2815_00648	K03214
NBC2815_00650	K01626
NBC2815_00653	K06207
NBC2815_00654	K03768
NBC2815_00655	K00024
NBC2815_00658	K00799
NBC2815_00660	K07481
NBC2815_00662	K00219

NBC2815_00663	K00344
NBC2815_00664	K03286
NBC2815_00666	K06178
NBC2815_00668	K00639
NBC2815_00669	K23163
NBC2815_00670	K02046
NBC2815_00671	K02047
NBC2815_00672	K02045
NBC2815_00674	K00060
NBC2815_00675	K02014
NBC2815_00676	K01114
NBC2815_00677	K22305
NBC2815_00679	K01214
NBC2815_00680	K05844
NBC2815_00684	K07497
NBC2815_00687	K00793
NBC2815_00688	K14652
NBC2815_00689	K00794
NBC2815_00690	K03625
NBC2815_00691	K00946
NBC2815_00693	K01546
NBC2815_00696	K02483
NBC2815_00697	K02484
NBC2815_00698	K01919
NBC2815_00699	K03978
NBC2815_00701	K03673
NBC2815_00702	K03673
NBC2815_00704	K00001
NBC2815_00706	K08309
NBC2815_00707	K00974
NBC2815_00714	K07481
NBC2815_00719	K02557
NBC2815_00720	K02556
NBC2815_00721	K07305
NBC2815_00723	K03719
NBC2815_00724	K00285
NBC2815_00725	K01775
NBC2815_00726	K07481
NBC2815_00735	K00564
NBC2815_00736	K06183
NBC2815_00741	K09941
NBC2815_00744	K09767
NBC2815_00747	K02071
NBC2815_00748	K02072
NBC2815_00749	K02073
NBC2815_00751	K07275
NBC2815_00753	K00627
NBC2815_00755	K00382
NBC2815_00759	K02108
NBC2815_00760	K02110

NBC2815_00761	K02109
NBC2815_00762	K02113
NBC2815_00763	K02111
NBC2815_00764	K02115
NBC2815_00765	K02112
NBC2815_00766	K02114
NBC2815_00767	K04093
NBC2815_00769	K04042
NBC2815_00773	K00820
NBC2815_00776	K01759
NBC2815_00777	K07233
NBC2815_00779	K01738
NBC2815_00780	K01414
NBC2815_00782	K00647
NBC2815_00783	K01716
NBC2815_00784	K02346
NBC2815_00785	K01091
NBC2815_00786	K07481
NBC2815_00791	K08968
NBC2815_00792	K01935
NBC2815_00794	K01737
NBC2815_00795	K11927
NBC2815_00796	K16171
NBC2815_00797	K01800
NBC2815_00798	K02670
NBC2815_00802	K01697
NBC2815_00803	K01758
NBC2815_00805	K09690
NBC2815_00806	K09691
NBC2815_00807	K18827
NBC2815_00811	K01711
NBC2815_00812	K22252
NBC2815_00816	K10012
NBC2815_00818	K03522
NBC2815_00819	K03521
NBC2815_00820	K01710
NBC2815_00821	K00973
NBC2815_00822	K01790
NBC2815_00823	K00067
NBC2815_00824	K16011
NBC2815_00825	K15778
NBC2815_00826	K01028
NBC2815_00827	K01029
NBC2815_00828	K07272
NBC2815_00829	K00311
NBC2815_00831	K18480
NBC2815_00832	K02067
NBC2815_00833	K02065
NBC2815_00834	K02066
NBC2815_00836	K03746

NBC2815_00837	K01881
NBC2815_00839	K17103
NBC2815_00841	K03789
NBC2815_00843	K07497
NBC2815_00847	K07043
NBC2815_00848	K01153
NBC2815_00849	K01154
NBC2815_00851	K14623
NBC2815_00852	K03427
NBC2815_00859	K12308
NBC2815_00861	K09704
NBC2815_00862	K07481
NBC2815_00864	K11754
NBC2815_00866	K03558
NBC2815_00867	K00764
NBC2815_00870	K23743
NBC2815_00872	K03269
NBC2815_00873	K19302
NBC2815_00875	K01524
NBC2815_00876	K00937
NBC2815_00877	K07636
NBC2815_00878	K07657
NBC2815_00879	K01423
NBC2815_00880	K03676
NBC2815_00882	K00030
NBC2815_00883	K07481
NBC2815_00895	K07483
NBC2815_00896	K07497
NBC2815_00907	K03695
NBC2815_00910	K16092
NBC2815_00911	K01586
NBC2815_00916	K16091
NBC2815_00917	K02510
NBC2815_00919	K07481
NBC2815_00925	K03781
NBC2815_00926	K00281
NBC2815_00927	K07552
NBC2815_00928	K07552
NBC2815_00930	K01283
NBC2815_00931	K03760
NBC2815_00939	K03608
NBC2815_00940	K03609
NBC2815_00941	K03610
NBC2815_00942	K06977
NBC2815_00948	K01247
NBC2815_00949	K07260
NBC2815_00955	K08289
NBC2815_00956	K07497
NBC2815_00963	K21420
NBC2815_00967	K10805

NBC2815_00969	K09796
NBC2815_00970	K07107
NBC2815_00971	K03701
NBC2815_00972	K02888
NBC2815_00973	K02899
NBC2815_00974	K03979
NBC2815_00975	K02968
NBC2815_00976	K03980
NBC2815_00977	K11753
NBC2815_00978	K01870
NBC2815_00979	K03101
NBC2815_00980	K03527
NBC2815_00982	K02297
NBC2815_00983	K02298
NBC2815_00984	K02299
NBC2815_00985	K02300
NBC2815_00987	K04485
NBC2815_01002	K07481
NBC2815_01005	K01599
NBC2815_01007	K01735
NBC2815_01008	K00891
NBC2815_01009	K00275
NBC2815_01010	K09980
NBC2815_01013	K08989
NBC2815_01020	K01337
NBC2815_01022	K07782
NBC2815_01023	K18457
NBC2815_01024	K03406
NBC2815_01027	K03293
NBC2815_01028	K01273
NBC2815_01029	K07481
NBC2815_01031	K03455
NBC2815_01032	K02014
NBC2815_01033	K02014
NBC2815_01036	K02014
NBC2815_01037	K06158
NBC2815_01040	K01876
NBC2815_01044	K01159
NBC2815_01045	K03550
NBC2815_01046	K03549
NBC2815_01047	K03551
NBC2815_01048	K07107
NBC2815_01049	K03562
NBC2815_01050	K03560
NBC2815_01051	K03646
NBC2815_01052	K03641
NBC2815_01053	K03640
NBC2815_01055	K10026
NBC2815_01057	K07481
NBC2815_01059	K03770

NBC2815_01063	K03530
NBC2815_01064	K01338
NBC2815_01065	K03544
NBC2815_01066	K01358
NBC2815_01067	K03545
NBC2815_01070	K07481
NBC2815_01073	K07481
NBC2815_01078	K11177
NBC2815_01079	K11178
NBC2815_01080	K13483
NBC2815_01081	K09003
NBC2815_01088	K01356
NBC2815_01089	K13053
NBC2815_01090	K14161
NBC2815_01091	K14162
NBC2815_01103	K10563
NBC2815_01105	K08307
NBC2815_01106	K01069
NBC2815_01108	K03469
NBC2815_01109	K02342
NBC2815_01110	K20074
NBC2815_01113	K12982
NBC2815_01114	K11211
NBC2815_01116	K03639
NBC2815_01118	K03637
NBC2815_01119	K03636
NBC2815_01120	K03635
NBC2815_01122	K02343
NBC2815_01123	K09747
NBC2815_01124	K06187
NBC2815_01125	K02503
NBC2815_01126	K07285
NBC2815_01129	K03924
NBC2815_01133	K06287
NBC2815_01134	K07040
NBC2815_01135	K02911
NBC2815_01136	K00648
NBC2815_01137	K00645
NBC2815_01138	K00059
NBC2815_01139	K02078
NBC2815_01140	K09458
NBC2815_01141	K01657
NBC2815_01142	K07082
NBC2815_01143	K02341
NBC2815_01144	K02676
NBC2815_01150	K03201
NBC2815_01155	K03201
NBC2815_01179	K13408
NBC2815_01180	K13409
NBC2815_01193	K07483

NBC2815_01194	K07497
NBC2815_01201	K17758
NBC2815_01206	K00384
NBC2815_01211	K09761
NBC2815_01212	K00833
NBC2815_01213	K08312
NBC2815_01214	K01082
NBC2815_01215	K04765
NBC2815_01216	K09771
NBC2815_01217	K00605
NBC2815_01218	K02437
NBC2815_01220	K01467
NBC2815_01221	K03301
NBC2815_01222	K08987
NBC2815_01223	K06445
NBC2815_01229	K07481
NBC2815_01234	K07497
NBC2815_01236	K04083
NBC2815_01237	K03814
NBC2815_01238	K20534
NBC2815_01240	K07290
NBC2815_01241	K11068
NBC2815_01242	K02837
NBC2815_01247	K00641
NBC2815_01248	K01739
NBC2815_01249	K00003
NBC2815_01252	K01752
NBC2815_01254	K01752
NBC2815_01257	K12262
NBC2815_01263	K01785
NBC2815_01265	K03106
NBC2815_01268	K00459
NBC2815_01270	K02959
NBC2815_01271	K02860
NBC2815_01272	K00554
NBC2815_01273	K02884
NBC2815_01275	K04762
NBC2815_01276	K07481
NBC2815_01284	K01868
NBC2815_01285	K02520
NBC2815_01286	K02916
NBC2815_01287	K02887
NBC2815_01288	K01889
NBC2815_01289	K01890
NBC2815_01290	K04764
NBC2815_01293	K01991
NBC2815_01294	K13661
NBC2815_01295	K13656
NBC2815_01296	K13662
NBC2815_01298	K13664

NBC2815_01299	K13657
NBC2815_01300	K13658
NBC2815_01301	K03328
NBC2815_01302	K13659
NBC2815_01303	K13665
NBC2815_01304	K13660
NBC2815_01305	K09705
NBC2815_01306	K01662
NBC2815_01312	K03098
NBC2815_01315	K06884
NBC2815_01318	K03575
NBC2815_01319	K03110
NBC2815_01321	K12658
NBC2815_01322	K21061
NBC2815_01323	K22550
NBC2815_01324	K22549
NBC2815_01325	K21062
NBC2815_01326	K13877
NBC2815_01327	K01271
NBC2815_01333	K07481
NBC2815_01335	K07481
NBC2815_01337	K08677
NBC2815_01346	K09955
NBC2815_01350	K07497
NBC2815_01354	K08973
NBC2815_01355	K01962
NBC2815_01356	K02337
NBC2815_01358	K03470
NBC2815_01359	K00748
NBC2815_01360	K00677
NBC2815_01361	K02372
NBC2815_01362	K02536
NBC2815_01364	K07277
NBC2815_01365	K11749
NBC2815_01366	K00099
NBC2815_01367	K00981
NBC2815_01368	K00806
NBC2815_01369	K02838
NBC2815_01370	K09903
NBC2815_01372	K02357
NBC2815_01373	K02967
NBC2815_01374	K07346
NBC2815_01377	K07347
NBC2815_01378	K07346
NBC2815_01380	K01265
NBC2815_01381	K00990
NBC2815_01382	K00674
NBC2815_01384	K01439
NBC2815_01385	K01953
NBC2815_01387	K02014

NBC2815_01388	K01434
NBC2815_01392	K03594
NBC2815_01394	K02621
NBC2815_01397	K03543
NBC2815_01398	K03446
NBC2815_01403	K03305
NBC2815_01406	K06975
NBC2815_01408	K15974
NBC2815_01409	K01284
NBC2815_01410	K00432
NBC2815_01411	K00528
NBC2815_01412	K06147
NBC2815_01413	K01676
NBC2815_01415	K01673
NBC2815_01417	K01058
NBC2815_01418	K09793
NBC2815_01420	K03704
NBC2815_01421	K06077
NBC2815_01423	K22132
NBC2815_01424	K03424
NBC2815_01428	K00799
NBC2815_01429	K01953
NBC2815_01430	K19784
NBC2815_01431	K02314
NBC2815_01432	K01669
NBC2815_01437	K07483
NBC2815_01441	K21686
NBC2815_01442	K00140
NBC2815_01446	K00020
NBC2815_01447	K16264
NBC2815_01448	K01782
NBC2815_01449	K03088
NBC2815_01451	K04771
NBC2815_01453	K07481
NBC2815_01455	K07483
NBC2815_01456	K07497
NBC2815_01458	K03284
NBC2815_01460	K06189
NBC2815_01462	K07042
NBC2815_01463	K06217
NBC2815_01464	K06168
NBC2815_01467	K00411
NBC2815_01468	K00412
NBC2815_01469	K00413
NBC2815_01470	K03599
NBC2815_01471	K03600
NBC2815_01473	K03724
NBC2815_01474	K03652
NBC2815_01481	K03684
NBC2815_01483	K07481

NBC2815_01484	K03596
NBC2815_01485	K03100
NBC2815_01487	K03685
NBC2815_01488	K03595
NBC2815_01489	K03584
NBC2815_01491	K03215
NBC2815_01492	K01768
NBC2815_01493	K18208
NBC2815_01494	K06876
NBC2815_01495	K01207
NBC2815_01496	K00760
NBC2815_01497	K19696
NBC2815_01498	K03704
NBC2815_01499	K06953
NBC2815_01500	K03724
NBC2815_01501	K10747
NBC2815_01503	K07577
NBC2815_01508	K00626
NBC2815_01509	K07481
NBC2815_01510	K00263
NBC2815_01512	K07043
NBC2815_01517	K06915
NBC2815_01518	K08307
NBC2815_01519	K08086
NBC2815_01520	K06996
NBC2815_01521	K06173
NBC2815_01522	K01817
NBC2815_01525	K07481
NBC2815_01528	K01696
NBC2815_01530	K01695
NBC2815_01531	K01963
NBC2815_01532	K03431
NBC2815_01537	K06886
NBC2815_01538	K07124
NBC2815_01539	K01803
NBC2815_01540	K03075
NBC2815_01542	K00330
NBC2815_01543	K00331
NBC2815_01544	K00332
NBC2815_01545	K00333
NBC2815_01546	K00334
NBC2815_01547	K00335
NBC2815_01548	K00336
NBC2815_01549	K00337
NBC2815_01550	K00338
NBC2815_01551	K00339
NBC2815_01552	K00340
NBC2815_01553	K00341
NBC2815_01554	K00342
NBC2815_01555	K00343

NBC2815_01557	K09748
NBC2815_01558	K02600
NBC2815_01559	K02519
NBC2815_01560	K02834
NBC2815_01561	K03177
NBC2815_01562	K02956
NBC2815_01563	K00962
NBC2815_01566	K00767
NBC2815_01568	K01588
NBC2815_01569	K01589
NBC2815_01570	K04564
NBC2815_01571	K07390
NBC2815_01575	K06966
NBC2815_01576	K08082
NBC2815_01577	K08084
NBC2815_01578	K02672
NBC2815_01579	K02674
NBC2815_01580	K02655
NBC2815_01582	K07814
NBC2815_01587	K00822
NBC2815_01591	K02379
NBC2815_01595	K07483
NBC2815_01596	K07497
NBC2815_01598	K09471
NBC2815_01599	K01915
NBC2815_01600	K01915
NBC2815_01601	K11073
NBC2815_01603	K03543
NBC2815_01604	K03446
NBC2815_01606	K11076
NBC2815_01607	K11075
NBC2815_01608	K11074
NBC2815_01610	K00135
NBC2815_01613	K18138
NBC2815_01614	K03585
NBC2815_01616	K15371
NBC2815_01621	K18376
NBC2815_01622	K07481
NBC2815_01630	K00425
NBC2815_01631	K07481
NBC2815_01633	K07483
NBC2815_01637	K03702
NBC2815_01642	K03203
NBC2815_01643	K03204
NBC2815_01644	K03195
NBC2815_01645	K03196
NBC2815_01646	K03194
NBC2815_01647	K03197
NBC2815_01648	K03198
NBC2815_01649	K03199

NBC2815_01650	K03200
NBC2815_01651	K03201
NBC2815_01654	K07481
NBC2815_01661	K03664
NBC2815_01663	K09801
NBC2815_01664	K06186
NBC2815_01665	K03711
NBC2815_01666	K03631
NBC2815_01667	K03705
NBC2815_01669	K03687
NBC2815_01670	K04043
NBC2815_01671	K03686
NBC2815_01672	K00868
NBC2815_01673	K04517
NBC2815_01677	K02021
NBC2815_01679	K04761
NBC2815_01680	K06147
NBC2815_01683	K00382
NBC2815_01684	K00658
NBC2815_01685	K00164
NBC2815_01687	K18850
NBC2815_01688	K01756
NBC2815_01689	K01679
NBC2815_01691	K07497
NBC2815_01698	K01992
NBC2815_01699	K01990
NBC2815_01700	K07979
NBC2815_01702	K00432
NBC2815_01703	K03772
NBC2815_01704	K00012
NBC2815_01705	K03745
NBC2815_01708	K02529
NBC2815_01709	K02429
NBC2815_01710	K00847
NBC2815_01712	K00548
NBC2815_01713	K00548
NBC2815_01718	K12573
NBC2815_01720	K03218
NBC2815_01722	K03683
NBC2815_01723	K02039
NBC2815_01724	K02036
NBC2815_01725	K02038
NBC2815_01726	K02037
NBC2815_01727	K02040
NBC2815_01729	K02040
NBC2815_01731	K01673
NBC2815_01733	K10773
NBC2815_01737	K01802
NBC2815_01739	K01011
NBC2815_01740	K09164

NBC2815_01741	K01447
NBC2815_01744	K03824
NBC2815_01746	K12297
NBC2815_01747	K00858
NBC2815_01748	K01081
NBC2815_01749	K07481
NBC2815_01752	K01141
NBC2815_01753	K00486
NBC2815_01754	K01556
NBC2815_01756	K00452
NBC2815_01757	K01673
NBC2815_01758	K07734
NBC2815_01762	K01893
NBC2815_01763	K13628
NBC2815_01764	K02990
NBC2815_01765	K02963
NBC2815_01766	K02939
NBC2815_01767	K03529
NBC2815_01768	K03528
NBC2815_01769	K01972
NBC2815_01770	K04568
NBC2815_01772	K08963
NBC2815_01773	K02469
NBC2815_01775	K01712
NBC2815_01776	K01458
NBC2815_01777	K01745
NBC2815_01778	K01468
NBC2815_01779	K05603
NBC2815_01780	K05836
NBC2815_01784	K17247
NBC2815_01785	K01613
NBC2815_01786	K07152
NBC2815_01787	K07320
NBC2815_01789	K01736
NBC2815_01790	K00090
NBC2815_01791	K00133
NBC2815_01793	K05874
NBC2815_01795	K08482
NBC2815_01797	K07497
NBC2815_01809	K07481
NBC2815_01815	K03407
NBC2815_01816	K03414
NBC2815_01817	K03413
NBC2815_01818	K02405
NBC2815_01819	K04562
NBC2815_01820	K02404
NBC2815_01821	K02400
NBC2815_01822	K02401
NBC2815_01825	K02421
NBC2815_01826	K02420

NBC2815_01827	K02419
NBC2815_01828	K02418
NBC2815_01829	K02417
NBC2815_01830	K02416
NBC2815_01831	K02415
NBC2815_01832	K02414
NBC2815_01833	K02413
NBC2815_01834	K02412
NBC2815_01835	K02411
NBC2815_01836	K02410
NBC2815_01837	K02409
NBC2815_01838	K02408
NBC2815_01839	K20444
NBC2815_01842	K10941
NBC2815_01844	K03092
NBC2815_01848	K02422
NBC2815_01849	K02407
NBC2815_01850	K02406
NBC2815_01851	K02397
NBC2815_01852	K02396
NBC2815_01853	K02395
NBC2815_01854	K02394
NBC2815_01855	K02393
NBC2815_01856	K02392
NBC2815_01857	K02391
NBC2815_01858	K02390
NBC2815_01859	K02389
NBC2815_01860	K02388
NBC2815_01861	K02387
NBC2815_01862	K03415
NBC2815_01863	K02386
NBC2815_01864	K02398
NBC2815_01873	K03406
NBC2815_01874	K07153
NBC2815_01875	K00566
NBC2815_01877	K06891
NBC2815_01878	K03694
NBC2815_01879	K02518
NBC2815_01880	K00684
NBC2815_01881	K09919
NBC2815_01882	K00384
NBC2815_01883	K03466
NBC2815_01886	K03634
NBC2815_01887	K07478
NBC2815_01888	K07481
NBC2815_01891	K00632
NBC2815_01892	K07516
NBC2815_01894	K00940
NBC2815_01895	K06941
NBC2815_01896	K02656

NBC2815_01897	K15539
NBC2815_01899	K17713
NBC2815_01900	K03977
NBC2815_01901	K03750
NBC2815_01905	K03750
NBC2815_01906	K03752
NBC2815_01908	K02304
NBC2815_01910	K07481
NBC2815_01915	K06884
NBC2815_01932	K11892
NBC2815_01933	K11893
NBC2815_01934	K11906
NBC2815_01936	K11901
NBC2815_01937	K11900
NBC2815_01938	K11903
NBC2815_01939	K11897
NBC2815_01940	K11896
NBC2815_01941	K11895
NBC2815_01942	K11907
NBC2815_01949	K07497
NBC2815_01950	K07483
NBC2815_01952	K07481
NBC2815_01954	K02474
NBC2815_01957	K03772
NBC2815_01959	K07146
NBC2815_01961	K08300
NBC2815_01962	K06179
NBC2815_01968	K07238
NBC2815_01969	K07497
NBC2815_01970	K07483
NBC2815_01972	K03832
NBC2815_01973	K01724
NBC2815_01974	K07400
NBC2815_01995	K02556
NBC2815_01996	K02557
NBC2815_01997	K03496
NBC2815_01998	K03408
NBC2815_02000	K03413
NBC2815_02001	K03407
NBC2815_02004	K05874
NBC2815_02005	K05874
NBC2815_02006	K05874
NBC2815_02008	K05874
NBC2815_02009	K05874
NBC2815_02012	K03408
NBC2815_02014	K00575
NBC2815_02015	K03411
NBC2815_02016	K03412
NBC2815_02019	K01682
NBC2815_02022	K01681

NBC2815_02024	K01897
NBC2815_02025	K13816
NBC2815_02026	K10715
NBC2815_02028	K13815
NBC2815_02029	K04567
NBC2815_02030	K02836
NBC2815_02031	K07462
NBC2815_02033	K03624
NBC2815_02034	K01955
NBC2815_02037	K01956
NBC2815_02038	K00215
NBC2815_02039	K05825
NBC2815_02041	K04759
NBC2815_02042	K04758
NBC2815_02043	K13766
NBC2815_02044	K01640
NBC2815_02046	K02356
NBC2815_02050	K00058
NBC2815_02052	K03294
NBC2815_02053	K03294
NBC2815_02054	K08964
NBC2815_02055	K08967
NBC2815_02056	K09880
NBC2815_02061	K11755
NBC2815_02062	K02500
NBC2815_02063	K01814
NBC2815_02064	K02501
NBC2815_02065	K01089
NBC2815_02066	K00817
NBC2815_02067	K00013
NBC2815_02068	K00765
NBC2815_02070	K01892
NBC2815_02071	K07481
NBC2815_02073	K07481
NBC2815_02074	K07497
NBC2815_02075	K07483
NBC2815_02076	K20266
NBC2815_02077	K07062
NBC2815_02078	K21495
NBC2815_02082	K07505
NBC2815_02085	K07733
NBC2815_02087	K01951
NBC2815_02088	K00088
NBC2815_02089	K01491
NBC2815_02090	K09948
NBC2815_02091	K07481
NBC2815_02092	K07481
NBC2815_02093	K07497
NBC2815_02095	K07497
NBC2815_02097	K00963

NBC2815_02100	K19804
NBC2815_02101	K08992
NBC2815_02102	K05788
NBC2815_02103	K02945
NBC2815_02104	K00945
NBC2815_02105	K02919
NBC2815_02108	K12251
NBC2815_02109	K03823
NBC2815_02113	K02026
NBC2815_02114	K02025
NBC2815_02115	K02027
NBC2815_02118	K02529
NBC2815_02119	K05527
NBC2815_02120	K09780
NBC2815_02121	K05896
NBC2815_02122	K06024
NBC2815_02123	K06178
NBC2815_02125	K22757
NBC2815_02126	K13566
NBC2815_02127	K14287
NBC2815_02128	K02193
NBC2815_02129	K02194
NBC2815_02130	K02195
NBC2815_02131	K02196
NBC2815_02132	K02197
NBC2815_02133	K02198
NBC2815_02134	K02199
NBC2815_02135	K02200
NBC2815_02136	K02200
NBC2815_02137	K07481
NBC2815_02138	K00641
NBC2815_02139	K07481
NBC2815_02144	K03406
NBC2815_02147	K06194
NBC2815_02149	K00573
NBC2815_02150	K03787
NBC2815_02152	K06176
NBC2815_02153	K01770
NBC2815_02154	K00991
NBC2815_02155	K05589
NBC2815_02156	K01689
NBC2815_02157	K01627
NBC2815_02158	K01937
NBC2815_02160	K02622
NBC2815_02163	K03306
NBC2815_02164	K07220
NBC2815_02165	K03699
NBC2815_02168	K08369
NBC2815_02175	K03088
NBC2815_02180	K02200

NBC2815_02184	K07497
NBC2815_02190	K01733
NBC2815_02192	K07481
NBC2815_02193	K00872
NBC2815_02194	K12524
NBC2815_02195	K07497
NBC2815_02197	K07481
NBC2815_02205	K07497
NBC2815_02206	K07483
NBC2815_02208	K00128
NBC2815_02211	K00254
NBC2815_02212	K00075
NBC2815_02215	K19302
NBC2815_02216	K03526
NBC2815_02217	K15011
NBC2815_02218	K15012
NBC2815_02228	K03307
NBC2815_02229	K05349
NBC2815_02230	K07093
NBC2815_02231	K03205
NBC2815_02236	K01810
NBC2815_02237	K01579
NBC2815_02238	K01918
NBC2815_02239	K00606
NBC2815_02240	K00950
NBC2815_02241	K00970
NBC2815_02244	K09007
NBC2815_02245	K08139
NBC2815_02246	K01805
NBC2815_02247	K00854
NBC2815_02248	K15923
NBC2815_02249	K01811
NBC2815_02251	K05970
NBC2815_02259	K01631
NBC2815_02260	K01684
NBC2815_02261	K13874
NBC2815_02262	K00883
NBC2815_02263	K05524
NBC2815_02265	K01714
NBC2815_02266	K03567
NBC2815_02268	K03564
NBC2815_02269	K07175
NBC2815_02271	K00941
NBC2815_02277	K06911
NBC2815_02280	K03563
NBC2815_02281	K01872
NBC2815_02282	K03565
NBC2815_02283	K03553
NBC2815_02284	K01356
NBC2815_02285	K03688

NBC2815_02287	K03568
NBC2815_02288	K07481
NBC2815_02289	K01728
NBC2815_02293	K07481
NBC2815_02294	K07497
NBC2815_02295	K07497
NBC2815_02306	K07481
NBC2815_02308	K03743
NBC2815_02309	K03665
NBC2815_02310	K03666
NBC2815_02311	K00791
NBC2815_02312	K00796
NBC2815_02313	K03798
NBC2815_02314	K02427
NBC2815_02315	K07574
NBC2815_02320	K07497
NBC2815_02326	K07481
NBC2815_02355	K13444
NBC2815_02359	K11902
NBC2815_02360	K03286
NBC2815_02361	K11890
NBC2815_02362	K11891
NBC2815_02365	K17733
NBC2815_02378	K07481
NBC2815_02387	K00995
NBC2815_02388	K03703
NBC2815_02390	K01104
NBC2815_02391	K00979
NBC2815_02392	K00912
NBC2815_02393	K11085
NBC2815_02394	K03559
NBC2815_02395	K03561
NBC2815_02396	K02238
NBC2815_02398	K09810
NBC2815_02399	K09808
NBC2815_02400	K19168
NBC2815_02401	K09159
NBC2815_02402	K00240
NBC2815_02403	K00239
NBC2815_02404	K00242
NBC2815_02405	K00241
NBC2815_02406	K06980
NBC2815_02407	K10112
NBC2815_02408	K00036
NBC2815_02409	K00845
NBC2815_02410	K01057
NBC2815_02411	K01690
NBC2815_02412	K01625
NBC2815_02417	K07481
NBC2815_02418	K07481

NBC2815_02422	K06959
NBC2815_02428	K03821
NBC2815_02429	K22881
NBC2815_02430	K17103
NBC2815_02431	K03574
NBC2815_02432	K09920
NBC2815_02433	K09773
NBC2815_02434	K01007
NBC2815_02435	K03442
NBC2815_02436	K13288
NBC2815_02437	K11991
NBC2815_02438	K11924
NBC2815_02439	K03322
NBC2815_02440	K00433
NBC2815_02442	K07481
NBC2815_02449	K07497
NBC2815_02453	K07483
NBC2815_02454	K07497
NBC2815_02456	K07481
NBC2815_02459	K00147
NBC2815_02460	K00931
NBC2815_02463	K01755
NBC2815_02464	K00145
NBC2815_02465	K22479
NBC2815_02466	K22478
NBC2815_02467	K01438
NBC2815_02468	K01940
NBC2815_02469	K09065
NBC2815_02471	K01883
NBC2815_02472	K02426
NBC2815_02474	K06204
NBC2815_02475	K01465
NBC2815_02477	K07481
NBC2815_02479	K01051
NBC2815_02481	K01728
NBC2815_02482	K01184
NBC2815_02484	K22292
NBC2815_02485	K00568
NBC2815_02486	K12960
NBC2815_02488	K02356
NBC2815_02490	K15396
NBC2815_02491	K01092
NBC2815_02492	K04564
NBC2815_02495	K15738
NBC2815_02496	K05591
NBC2815_02497	K00759
NBC2815_02500	K00799
NBC2815_02501	K03704
NBC2815_02505	K21025
NBC2815_02506	K03799

NBC2815_02507	K01894
NBC2815_02508	K00023
NBC2815_02510	K09973
NBC2815_02511	K09164
NBC2815_02512	K03572
NBC2815_02513	K01448
NBC2815_02514	K06925
NBC2815_02515	K17758
NBC2815_02516	K18979
NBC2815_02517	K03601
NBC2815_02520	K09125
NBC2815_02523	K00547
NBC2815_02524	K16235
NBC2815_02526	K01874
NBC2815_02528	K03616
NBC2815_02531	K03088
NBC2815_02533	K00507
NBC2815_02534	K06954
NBC2815_02535	K09701
NBC2815_02536	K00574
NBC2815_02539	K00574
NBC2815_02542	K14205
NBC2815_02545	K07481
NBC2815_02546	K01975
NBC2815_02549	K19577
NBC2815_02550	K10680
NBC2815_02551	K06193
NBC2815_02553	K04046
NBC2815_02556	K04760
NBC2815_02557	K14441
NBC2815_02559	K01061
NBC2815_02563	K01494
NBC2815_02564	K03593
NBC2815_02567	K07386
NBC2815_02568	K07386
NBC2815_02570	K00383
NBC2815_02571	K00285
NBC2815_02572	K07148
NBC2815_02573	K03775
NBC2815_02578	K07483
NBC2815_02592	K01875
NBC2815_02594	K03832
NBC2815_02595	K00800
NBC2815_02596	K14170
NBC2815_02597	K00831
NBC2815_02598	K07147
NBC2815_02600	K03435
NBC2815_02601	K08483
NBC2815_02602	K00882
NBC2815_02603	K02770

NBC2815_02604	K07267
NBC2815_02605	K03074
NBC2815_02606	K03072
NBC2815_02607	K03210
NBC2815_02608	K00773
NBC2815_02609	K07568
NBC2815_02613	K00761
NBC2815_02616	K01179
NBC2815_02617	K00681
NBC2815_02620	K00954
NBC2815_02621	K08316
NBC2815_02622	K04079
NBC2815_02624	K05973
NBC2815_02627	K07481
NBC2815_02631	K01077
NBC2815_02632	K04075
NBC2815_02633	K03602
NBC2815_02634	K00795
NBC2815_02635	K08646
NBC2815_02636	K09940
NBC2815_02637	K03592
NBC2815_02638	K09889
NBC2815_02639	K03568
NBC2815_02642	K08301
NBC2815_02643	K06287
NBC2815_02644	K09807
NBC2815_02645	K00783
NBC2815_02646	K07497
NBC2815_02650	K09710
NBC2815_02651	K00969
NBC2815_02652	K02340
NBC2815_02653	K03643
NBC2815_02654	K01869
NBC2815_02656	K05838
NBC2815_02658	K18377
NBC2815_02659	K18378
NBC2815_02660	K03219
NBC2815_02661	K03228
NBC2815_02663	K03224
NBC2815_02664	K03223
NBC2815_02666	K03222
NBC2815_02667	K18374
NBC2815_02668	K18373
NBC2815_02669	K03229
NBC2815_02670	K03230
NBC2815_02671	K18381
NBC2815_02672	K03225
NBC2815_02673	K03226
NBC2815_02674	K03227
NBC2815_02675	K18379

NBC2815_02676	K03220
NBC2815_02679	K18380
NBC2815_02686	K09779
NBC2815_02687	K01802
NBC2815_02693	K18893
NBC2815_02701	K06182
NBC2815_02703	K05592
NBC2815_02704	K03088
NBC2815_02705	K14761
NBC2815_02711	K00567
NBC2815_02715	K02014
NBC2815_02717	K00123
NBC2815_02726	K01179
NBC2815_02727	K07481
NBC2815_02728	K09823
NBC2815_02729	K01885
NBC2815_02733	K07115
NBC2815_02735	K03723
NBC2815_02736	K03555
NBC2815_02737	K03782
NBC2815_02745	K01834
NBC2815_02751	K06911
NBC2815_02756	K23676
NBC2815_02760	K07141
NBC2815_02762	K07402
NBC2815_02765	K03111
NBC2815_02766	K02523
NBC2815_02768	K23169
NBC2815_02769	K23170
NBC2815_02771	K12526
NBC2815_02775	K04063
NBC2815_02776	K00609
NBC2815_02777	K07447
NBC2815_02778	K07735
NBC2815_02779	K01246
NBC2815_02782	K02670
NBC2815_02783	K02669
NBC2815_02784	K06997
NBC2815_02785	K00286
NBC2815_02787	K21472
NBC2815_02790	K07117
NBC2815_02792	K09014
NBC2815_02793	K09013
NBC2815_02794	K09015
NBC2815_02795	K11717
NBC2815_02798	K05710
NBC2815_02799	K16090
NBC2815_02800	K07336
NBC2815_02801	K07126
NBC2815_02802	K07497

NBC2815_02804	K03734
NBC2815_02805	K09975
NBC2815_02806	K02237
NBC2815_02809	K07102
NBC2815_02810	K00992
NBC2815_02811	K10763
NBC2815_02813	K09938
NBC2815_02814	K01933
NBC2815_02815	K11175
NBC2815_02821	K00790
NBC2815_02823	K06041
NBC2815_02824	K03270
NBC2815_02825	K11719
NBC2815_02826	K09774
NBC2815_02827	K06861
NBC2815_02828	K03092
NBC2815_02829	K05808
NBC2815_02830	K02806
NBC2815_02831	K06023
NBC2815_02832	K06958
NBC2815_02833	K02821
NBC2815_02834	K11189
NBC2815_02835	K08483
NBC2815_02836	K06213
NBC2815_02838	K07497
NBC2815_02842	K03205
NBC2815_02843	K00845
NBC2815_02845	K02014
NBC2815_02846	K01206
NBC2815_02848	K12373
NBC2815_02849	K01192
NBC2815_02850	K05349
NBC2815_02852	K07497
NBC2815_02857	K07481
NBC2815_02859	K07497
NBC2815_02860	K07483
NBC2815_02863	K01256
NBC2815_02864	K01939
NBC2815_02865	K09937
NBC2815_02866	K04087
NBC2815_02867	K04088
NBC2815_02868	K02658
NBC2815_02869	K05516
NBC2815_02870	K13993
NBC2815_02872	K03594
NBC2815_02876	K02014
NBC2815_02877	K09788
NBC2815_02878	K20455
NBC2815_02879	K01659
NBC2815_02880	K03417

NBC2815_02881	K02688
NBC2815_02883	K07481
NBC2815_02898	K12686
NBC2815_02899	K03154
NBC2815_02900	K03149
NBC2815_02901	K03439
NBC2815_02905	K03282
NBC2815_02911	K03611
NBC2815_02912	K02879
NBC2815_02913	K03040
NBC2815_02914	K02986
NBC2815_02915	K02948
NBC2815_02916	K02952
NBC2815_02917	K03076
NBC2815_02918	K02876
NBC2815_02919	K02907
NBC2815_02920	K02988
NBC2815_02921	K02881
NBC2815_02922	K02933
NBC2815_02923	K02994
NBC2815_02924	K02954
NBC2815_02925	K02931
NBC2815_02926	K02895
NBC2815_02927	K02874
NBC2815_02928	K02961
NBC2815_02929	K02904
NBC2815_02930	K02878
NBC2815_02931	K02982
NBC2815_02932	K02890
NBC2815_02933	K02965
NBC2815_02934	K02886
NBC2815_02935	K02892
NBC2815_02936	K02926
NBC2815_02937	K02906
NBC2815_02938	K02946
NBC2815_02939	K02358
NBC2815_02940	K02355
NBC2815_02941	K02992
NBC2815_02942	K02950
NBC2815_02943	K03046
NBC2815_02944	K03043
NBC2815_02945	K02935
NBC2815_02946	K02864
NBC2815_02947	K02863
NBC2815_02948	K02867
NBC2815_02949	K02601
NBC2815_02950	K03073
NBC2815_02952	K02358
NBC2815_02956	K06942
NBC2815_02957	K01056

NBC2815_02958	K02897
NBC2815_02959	K00948
NBC2815_02961	K00919
NBC2815_02962	K02494
NBC2815_02964	K02492
NBC2815_02965	K02835
NBC2815_02967	K03638
NBC2815_02971	K07058
NBC2815_02972	K03809
NBC2815_02974	K01424
NBC2815_02975	K03976
NBC2815_02976	K14645
NBC2815_02980	K00826
NBC2815_02984	K00325
NBC2815_02985	K03088
NBC2815_02991	K01515
NBC2815_02994	K01259
NBC2815_02995	K02493
NBC2815_02996	K03386
NBC2815_02997	K03387
NBC2815_02998	K04761
NBC2815_02999	K06140
NBC2815_03000	K00616
NBC2815_03001	K07304
NBC2815_03006	K00799
NBC2815_03007	K01886
NBC2815_03010	K06968
NBC2815_03011	K00118
NBC2815_03015	K02623
NBC2815_03016	K04100
NBC2815_03017	K11475
NBC2815_03018	K03184
NBC2815_03019	K03185
NBC2815_03022	K00798
NBC2815_03024	K04744
NBC2815_03025	K03771
NBC2815_03026	K00097
NBC2815_03027	K02528
NBC2815_03028	K06195
NBC2815_03029	K01525
NBC2815_03030	K00287
NBC2815_03032	K00560
NBC2815_03033	K13292
NBC2815_03035	K06872
NBC2815_03036	K00852
NBC2815_03037	K03317
NBC2815_03040	K16087
NBC2815_03041	K09922
NBC2815_03042	K07481
NBC2815_03043	K07481

NBC2815_03047	K03119
NBC2815_03049	K02004
NBC2815_03050	K02003
NBC2815_03051	K10804
NBC2815_03056	K00901
NBC2815_03057	K03744
NBC2815_03058	K05540
NBC2815_03060	K07038
NBC2815_03061	K00789
NBC2815_03066	K01595
NBC2815_03068	K01251
NBC2815_03069	K01014
NBC2815_03074	K09912
NBC2815_03075	K01176
NBC2815_03076	K00035
NBC2815_03077	K07481
NBC2815_03083	K11752
NBC2815_03085	K07738
NBC2815_03086	K00600
NBC2815_03088	K06020
NBC2815_03089	K01462
NBC2815_03092	K00763
NBC2815_03096	K02014
NBC2815_03097	K03811
NBC2815_03100	K02453
NBC2815_03101	K02463
NBC2815_03102	K02462
NBC2815_03103	K02461
NBC2815_03104	K02460
NBC2815_03105	K02459
NBC2815_03106	K02458
NBC2815_03107	K02457
NBC2815_03108	K02456
NBC2815_03109	K02455
NBC2815_03110	K02454
NBC2815_03111	K14645
NBC2815_03112	K21449
NBC2815_03114	K14645
NBC2815_03115	K01952
NBC2815_03116	K03981
NBC2815_03117	K04763
NBC2815_03119	K11720
NBC2815_03120	K07091
NBC2815_03121	K01255
NBC2815_03122	K02339
NBC2815_03123	K01873
NBC2815_03124	K07497
NBC2815_03125	K07483
NBC2815_03132	K11904
NBC2815_03134	K01206

NBC2815_03137	K06201
NBC2815_03138	K01444
NBC2815_03139	K03790
NBC2815_03140	K06598
NBC2815_03141	K06597
NBC2815_03142	K06596
NBC2815_03143	K02660
NBC2815_03144	K02659
NBC2815_03145	K02658
NBC2815_03146	K02657
NBC2815_03147	K01920
NBC2815_03148	K03832
NBC2815_03149	K14742
NBC2815_03150	K03722
NBC2815_03152	K05365
NBC2815_03153	K20444
NBC2815_03155	K00951
NBC2815_03156	K06136
NBC2815_03157	K06137
NBC2815_03158	K06138
NBC2815_03159	K06139
NBC2815_03160	K03578
NBC2815_03161	K04047
NBC2815_03162	K03654
NBC2815_03166	K06182
NBC2815_03170	K06920
NBC2815_03173	K07497
NBC2815_03175	K07461
NBC2815_03176	K00799
NBC2815_03177	K06901
NBC2815_03180	K02014
NBC2815_03181	K01087
NBC2815_03183	K00697
NBC2815_03185	K00117
NBC2815_03186	K05810
NBC2815_03187	K06180
NBC2815_03188	K05807
NBC2815_03189	K01916
NBC2815_03191	K01902
NBC2815_03192	K01903
NBC2815_03193	K02668
NBC2815_03194	K02667
NBC2815_03196	K04095
NBC2815_03197	K07481
NBC2815_03198	K02652
NBC2815_03199	K02650
NBC2815_03200	K02653
NBC2815_03201	K02654
NBC2815_03202	K00859
NBC2815_03204	K07497

NBC2815_03205	K07481
NBC2815_03206	K18879
NBC2815_03210	K00297
NBC2815_03212	K03574
NBC2815_03214	K03070
NBC2815_03217	K02535
NBC2815_03218	K03531
NBC2815_03219	K03590
NBC2815_03220	K03589
NBC2815_03221	K01921
NBC2815_03222	K01924
NBC2815_03223	K02563
NBC2815_03224	K03588
NBC2815_03225	K01000
NBC2815_03226	K01929
NBC2815_03227	K01928
NBC2815_03228	K03587
NBC2815_03229	K03586
NBC2815_03230	K03438
NBC2815_03231	K03925
NBC2815_03233	K19164
NBC2815_03234	K19163
NBC2815_03235	K07056
NBC2815_03236	K07121
NBC2815_03237	K07460
NBC2815_03238	K07481
NBC2815_03242	K07481
NBC2815_03243	K02666
NBC2815_03244	K02665
NBC2815_03245	K02664
NBC2815_03246	K02663
NBC2815_03247	K02662
NBC2815_03248	K05366
NBC2815_03250	K01647
NBC2815_03251	K02909
NBC2815_03252	K01239
NBC2815_03256	K03655
NBC2815_03257	K09022
NBC2815_03258	K01139
NBC2815_03259	K03060
NBC2815_03260	K00942
NBC2815_03262	K00989
NBC2815_03263	K02428
NBC2815_03266	K01271
NBC2815_03267	K01262
NBC2815_03268	K09895
NBC2815_03269	K09892
NBC2815_03270	K09888
NBC2815_03271	K01934
NBC2815_03273	K01807

NBC2815_03274	K09005
NBC2815_03277	K00788
NBC2815_03278	K01845
NBC2815_03279	K00821
NBC2815_03280	K10716
NBC2815_03281	K07481
NBC2815_03283	K13775
NBC2815_03286	K07157
NBC2815_03287	K02558
NBC2815_03288	K00939
NBC2815_03289	K21071
NBC2815_03292	K15987
NBC2815_03294	K01507
NBC2815_03296	K02014
NBC2815_03298	K07506
NBC2815_03299	K07481
NBC2815_03301	K03147
NBC2815_03308	K05875
NBC2815_03310	K00053
NBC2815_03311	K01652
NBC2815_03312	K11258
NBC2815_03313	K01754
NBC2815_03314	K01649
NBC2815_03315	K07481
NBC2815_03318	K00052
NBC2815_03319	K01704
NBC2815_03320	K01703
NBC2815_03323	K00573
NBC2815_03324	K12340
NBC2815_03325	K02527
NBC2815_03326	K02517
NBC2815_03331	K00029
NBC2815_03332	K11103
NBC2815_03333	K07221
NBC2815_03334	K10819
NBC2815_03336	K07497
NBC2815_03337	K07497
NBC2815_03339	K07481
NBC2815_03340	K07483
NBC2815_03345	K13409
NBC2815_03346	K13408
NBC2815_03351	K07483
NBC2815_03355	K02012
NBC2815_03356	K07649
NBC2815_03357	K07774
NBC2815_03358	K07221
NBC2815_03359	K03300
NBC2815_03360	K00023
NBC2815_03362	K16211
NBC2815_03364	K05341

NBC2815_03366	K06949
NBC2815_03371	K11003
NBC2815_03372	K11004
NBC2815_03373	K14260
NBC2815_03377	K07390
NBC2815_03379	K07497
NBC2815_03394	K01070
NBC2815_03395	K03396
NBC2815_03396	K00121
NBC2815_03397	K23239
NBC2815_03400	K01443
NBC2815_03401	K00820
NBC2815_03402	K02529
NBC2815_03404	K03710
NBC2815_03407	K07483
NBC2815_03409	K02463
NBC2815_03410	K02462
NBC2815_03415	K03793
NBC2815_03416	K00950
NBC2815_03418	K02485
NBC2815_03420	K04065
NBC2815_03422	K00033
NBC2815_03427	K03462
NBC2815_03428	K13522
NBC2815_03429	K03797
NBC2815_03435	K03644
NBC2815_03436	K03801
NBC2815_03437	K09158
NBC2815_03440	K07258
NBC2815_03441	K03642
NBC2815_03442	K08305
NBC2815_03443	K01184
NBC2815_03444	K05837
NBC2815_03445	K05515
NBC2815_03446	K03571
NBC2815_03447	K03570
NBC2815_03448	K03569
NBC2815_03449	K00856
NBC2815_03454	K03183
NBC2815_03457	K02348
NBC2815_03458	K03365
NBC2815_03460	K03667
NBC2815_03461	K01419
NBC2815_03462	K03733
NBC2815_03463	K09921
NBC2815_03464	K01778
NBC2815_03466	K01354
NBC2815_03470	K01322
NBC2815_03472	K03098
NBC2815_03474	K07283

NBC2815_03476	K01749
NBC2815_03477	K08083
NBC2815_03479	K06999
NBC2815_03480	K03669
NBC2815_03481	K03321
NBC2815_03482	K01179
NBC2815_03489	K05522
NBC2815_03492	K01194
NBC2815_03495	K03284
NBC2815_03497	K11737
NBC2815_03500	K15270
NBC2815_03501	K06983
NBC2815_03510	K07481
NBC2815_03515	K07481
NBC2815_03519	K03786
NBC2815_03520	K02160
NBC2815_03522	K01961
NBC2815_03523	K02687
NBC2815_03527	K03557
NBC2815_03530	K00602
NBC2815_03531	K01945
NBC2815_03540	K03807
NBC2815_03541	K03426
NBC2815_03546	K07483
NBC2815_03547	K07497
NBC2815_03548	K19736
NBC2815_03550	K01284
NBC2815_03553	K15034
NBC2815_03554	K09924
NBC2815_03555	K06175
NBC2815_03560	K03688
NBC2815_03561	K03690
NBC2815_03563	K01921
NBC2815_03566	K22320
NBC2815_03567	K22319
NBC2815_03568	K06940
NBC2815_03569	K22318
NBC2815_03570	K07497
NBC2815_03573	K15724
NBC2815_03579	K03594
NBC2815_03580	K08311
NBC2815_03583	K02996
NBC2815_03584	K02871
NBC2815_03585	K06134
NBC2815_03586	K11741
NBC2815_03587	K01611
NBC2815_03588	K10914
NBC2815_03589	K18697
NBC2815_03590	K01609
NBC2815_03591	K00766

NBC2815_03593	K01658
NBC2815_03596	K03430
NBC2815_03597	K01740
NBC2815_03599	K07010
NBC2815_03601	K00651
NBC2815_03602	K01915
NBC2815_03604	K01657
NBC2815_03607	K01783
NBC2815_03610	K01923
NBC2815_03618	K05559
NBC2815_03619	K05560
NBC2815_03620	K05561
NBC2815_03621	K05562
NBC2815_03622	K05563
NBC2815_03623	K05564
NBC2815_03624	K07481
NBC2815_03625	K07025
NBC2815_03630	K08641
NBC2815_03631	K01447
NBC2815_03633	K19802
NBC2815_03640	K05349
NBC2815_03641	K01633
NBC2815_03642	K01409
NBC2815_03643	K02970
NBC2815_03644	K09117
NBC2815_03645	K07058
NBC2815_03646	K02316
NBC2815_03648	K14347
NBC2815_03649	K01829
NBC2815_03650	K02257
NBC2815_03651	K02259
NBC2815_03655	K02276
NBC2815_03656	K02258
NBC2815_03658	K02274
NBC2815_03659	K02275
NBC2815_03661	K13821
NBC2815_03662	K01866
NBC2815_03664	K09001
NBC2815_03666	K08218
NBC2815_03667	K01142
NBC2815_03668	K00762
NBC2815_03670	K03496
NBC2815_03671	K03497
NBC2815_03675	K08679
NBC2815_03676	K00721
NBC2815_03679	K15778
NBC2815_03680	K01520
NBC2815_03681	K13038
NBC2815_03682	K03630
NBC2815_03683	K01887

NBC2815_03687	K05886
NBC2815_03694	K02364
NBC2815_03697	K07637
NBC2815_03698	K07660
NBC2815_03700	K05539
NBC2815_03702	K07481
NBC2815_03705	K07483
NBC2815_03707	K07497
NBC2815_03711	K03525
NBC2815_03712	K03524
NBC2815_03720	K01728
NBC2815_03721	K07481
NBC2815_03723	K03657
NBC2815_03725	K06131
NBC2815_03727	K02913
NBC2815_03728	K02902
NBC2815_03731	K03501
NBC2815_03732	K01113
NBC2815_03733	K06133
NBC2815_03736	K01142
NBC2815_03738	K14393
NBC2815_03741	K01895
NBC2815_03743	K00064
NBC2815_03744	K07046
NBC2815_03745	K18335
NBC2815_03746	K18336
NBC2815_03747	K18334
NBC2815_03748	K03534
NBC2815_03749	K02429
NBC2815_03753	K18786
NBC2815_03754	K03292
NBC2815_03755	K00851
NBC2815_03756	K05889
NBC2815_03758	K09800
NBC2815_03759	K07278
NBC2815_03763	K07566
NBC2815_03764	K03168
NBC2815_03769	K03747
NBC2815_03770	K04096
NBC2815_03772	K01462
NBC2815_03773	K00604
NBC2815_03774	K03500
NBC2815_03776	K02847
NBC2815_03779	K08981
NBC2815_03780	K09167
NBC2815_03781	K01497
NBC2815_03782	K03823
NBC2815_03783	K02517
NBC2815_03784	K07560
NBC2815_03785	K03086

NBC2815_03805	K06908
NBC2815_03806	K06907
NBC2815_03807	K06903
NBC2815_03817	K03791
NBC2815_03828	K00571
NBC2815_03848	K11209
NBC2815_03851	K14266
NBC2815_03853	K03832
NBC2815_03854	K09696
NBC2815_03855	K09697
NBC2815_03858	K01990
NBC2815_03859	K01992
NBC2815_03861	K00077
NBC2815_03867	K00451
NBC2815_03868	K00457
NBC2815_03872	K03305
NBC2815_03873	K00453
NBC2815_03874	K07004
NBC2815_03875	K00161
NBC2815_03876	K00161
NBC2815_03877	K00162
NBC2815_03879	K00627
NBC2815_03890	K00059
NBC2815_03891	K07497
NBC2815_03900	K08151
NBC2815_03901	K08151
NBC2815_03902	K01792
NBC2815_03904	K09858
NBC2815_03905	K07090
NBC2815_03913	K01495
NBC2815_03914	K03761
NBC2815_03920	K07497
NBC2815_03922	K07481
NBC2815_03924	K07481
NBC2815_03928	K21498
NBC2815_03929	K07334
NBC2815_03930	K03581
NBC2815_03931	K03582
NBC2815_03932	K03583
NBC2815_03933	K02065
NBC2815_03934	K02066
NBC2815_03935	K02067
NBC2815_03936	K07323
NBC2815_03937	K07122
NBC2815_03938	K04754
NBC2815_03940	K00432
NBC2815_03942	K09760
NBC2815_03944	K03294
NBC2815_03947	K00865
NBC2815_03950	K06969

NBC2815_03951	K05794
NBC2815_03952	K01126
NBC2815_03953	K07497
NBC2815_03957	K03650
NBC2815_03958	K03217
NBC2815_03959	K03536
NBC2815_03960	K02914

Table S9. Primers designed and used for validation of RNA-seq data by RT-qPCR

Gene ID or locus tag	Name/Putative function	Forward primer 5'-3'	Reverse primer 5'-3'	Amplicon length (bp)*
ahpF	alkyl hydroperoxide reductase	TCATCACCAGCGCACAGACTACAG	AACGCCACCGATCCCTTGAG	169
cysI	sulfite reductase (NADPH) hemoprotein subunit beta	CGGGCAGCGAAGAAGAACC	CGCAGGATCGCAATGAAACC	130
dapE	succinyl-diaminopimelate desuccinylase	GCGCGTTGCCGAGTTGTTTC	CATGCCCCTGTACGCCCTTCAC	155
flgJ	flagellar rod assembly protein	CCGCAAAGATCGACAAGGTG	CGTACATCTCGCGGAACATC	132
flhF	flagellar basal body rod protein FlgF	CTCTGGAGCTAATGGACGACTACG	GACAGCAGGCCCAGCATCAACC	118
glyA	glycine/serine hydroxymethyltransferase	AGCCGGGCGACACCATTCTG	AGGCCCTGCGCGTTGACAC	124
gyrA	DNA gyrase subunit A	TGGCCGACATCGACAAGGAGAC	GGCCATACCCACCGCAATACC	134
*gyrB***	DNA gyrase subunit B	TCCACTACGAAGGCGGCATCC	GGTAGGCGTCGGTCCATTG	141
ffh **	signal recognition particle protein	ATCAACCCGACCGAGACACTT TTC	GCCGTCGGTCTTGGTCAGCACTAC	123
hfg	RNA-binding protein Hfq	GGGTCCCGGTTTCTGTGTATCTG	GGCGTGCTTGTAAACCATCTGAC	119
iroN2	dependent receptor	GCTTGACGTCTGCGCTGCTTTTC	TGAGGCTGCCACGGATACC	153
oar	Oar protein	CGGCCGATTATGGATATGAAGATG	CGCCAGCCACGAATAGGAATAGAC	144
pilE	type IV pilin pile protein	ATCAATCATCCGTTATCAAGTCTC	ATGCTGGCTCCAACGTAAGTAGTG	115
pilW	type IV pilus assembly protein PilW	TCGCTTCGGATAACACCTCTG	GCAACCGAACTGACCTGAAAAATC	133
prnA_2	tryptophan halogenase	CCACCCAGGCCACCATCAAG	CGCGCAGCTCCAGACAGTAATC	186
proC	pyrroline-5-carboxylate reductase	GCGCGATGCCCAACACG	GTCCTCGATCCAAACGGTCAC	137

pykA **	pyruvate kinase	TGACCGAGCGCGACAAGGAG	AGAACGACACTGCGATGAAG	72
raxA	membrane fusion transmembrane protein	TATTAAAGCCCCGACTGATG	AACAAAGCCACCTGACTCTG	151
rluD	ribosomal large subunit pseudouridine synthase D	CGATGCGCTGTTGGATGG	CACGTTCAGCGGGATGTC	127
rpoB	DNA-directed RNA polymerase subunit beta	GAGCGTCTGCGTGGTGAAAC	TGACGTGACGCGCAGTGATG	94
NBC2815_00637	ISXo1 transposase, IS5 family	CAACGCCTGACGGTGGTGT	CTCGCGGGTGGGGTAGC	132

*Annealing temperature: 59°C
**Kałużna et al., 2019
